# Anticancer pan-ErbB inhibitors reduce inflammation and tissue injury and exert broad-spectrum antiviral effects

**DOI:** 10.1172/JCI169510

**Published:** 2023-10-02

**Authors:** Sirle Saul, Marwah Karim, Luca Ghita, Pei-Tzu Huang, Winston Chiu, Verónica Durán, Chieh-Wen Lo, Sathish Kumar, Nishank Bhalla, Pieter Leyssen, Farhang Alem, Niloufar A. Boghdeh, Do H.N. Tran, Courtney A. Cohen, Jacquelyn A. Brown, Kathleen E. Huie, Courtney Tindle, Mamdouh Sibai, Chengjin Ye, Ahmed Magdy Khalil, Kevin Chiem, Luis Martinez-Sobrido, John M. Dye, Benjamin A. Pinsky, Pradipta Ghosh, Soumita Das, David E. Solow-Cordero, Jing Jin, John P. Wikswo, Dirk Jochmans, Johan Neyts, Steven De Jonghe, Aarthi Narayanan, Shirit Einav

**Affiliations:** 1 Department of Medicine, Division of Infectious Diseases and Geographic Medicine, Stanford University, Stanford, California, USA.; 2 KU Leuven, Department of Microbiology, Immunology and Transplantation, Rega Institute for Medical Research, Laboratory of Virology and Chemotherapy, Leuven, Belgium.; 3 Chan Zuckerberg Biohub, San Francisco, California, USA.; 4 National Center for Biodefense and Infectious Disease, Biomedical Research Laboratory, and; 5 Institute for Biohealth Innovation, George Mason University, Manassas, Virginia, USA.; 6 US Army Medical Research Institute of Infectious Diseases, Viral Immunology Branch, Frederick, Maryland, USA.; 7 Department of Physics and Astronomy, Vanderbilt Institute for Integrative Biosystems Research and Education, Vanderbilt University, Nashville, Tennessee, USA.; 8 Department of Cellular and Molecular Medicine and; 9 HUMANOID Center of Research Excellence, UCSD, San Diego, California, USA.; 10 Department of Pathology, Stanford University School of Medicine, Stanford, California, USA.; 11 Disease Prevention and Intervention, Texas Biomedical Research Institute, San Antonio, Texas, USA.; 12 Department of Medicine and; 13 Department of Pathology, UCSD, San Diego, California, USA.; 14 Department of Sarafan ChEM-H, Stanford University, Stanford, California, USA.; 15 Vitalant Research Institute, San Francisco, California, USA.; 16 Department of Biomedical Engineering, Department of Molecular Physiology and Biophysics, and Department of Physics and Astronomy, Vanderbilt Institute for Integrative Biosystems Research and Education, Vanderbilt University, Nashville, Tennessee, USA.; 17 School of Systems Biology, George Mason University, Manassas, Virginia, USA.; 18 Department of Microbiology and Immunology, Stanford University, Stanford, California, USA.

**Keywords:** Therapeutics, Virology, Drug screens, Protein kinases

## Abstract

Targeting host factors exploited by multiple viruses could offer broad-spectrum solutions for pandemic preparedness. Seventeen candidates targeting diverse functions emerged in a screen of 4,413 compounds for SARS-CoV-2 inhibitors. We demonstrated that lapatinib and other approved inhibitors of the ErbB family of receptor tyrosine kinases suppress replication of SARS-CoV-2, Venezuelan equine encephalitis virus (VEEV), and other emerging viruses with a high barrier to resistance. Lapatinib suppressed SARS-CoV-2 entry and later stages of the viral life cycle and showed synergistic effect with the direct-acting antiviral nirmatrelvir. We discovered that ErbB1, ErbB2, and ErbB4 bind SARS-CoV-2 S1 protein and regulate viral and ACE2 internalization, and they are required for VEEV infection. In human lung organoids, lapatinib protected from SARS-CoV-2–induced activation of ErbB-regulated pathways implicated in non-infectious lung injury, proinflammatory cytokine production, and epithelial barrier injury. Lapatinib suppressed VEEV replication, cytokine production, and disruption of blood-brain barrier integrity in microfluidics-based human neurovascular units, and reduced mortality in a lethal infection murine model. We validated lapatinib-mediated inhibition of ErbB activity as an important mechanism of antiviral action. These findings reveal regulation of viral replication, inflammation, and tissue injury via ErbBs and establish a proof of principle for a repurposed, ErbB-targeted approach to combat emerging viruses.

## Graphical abstract

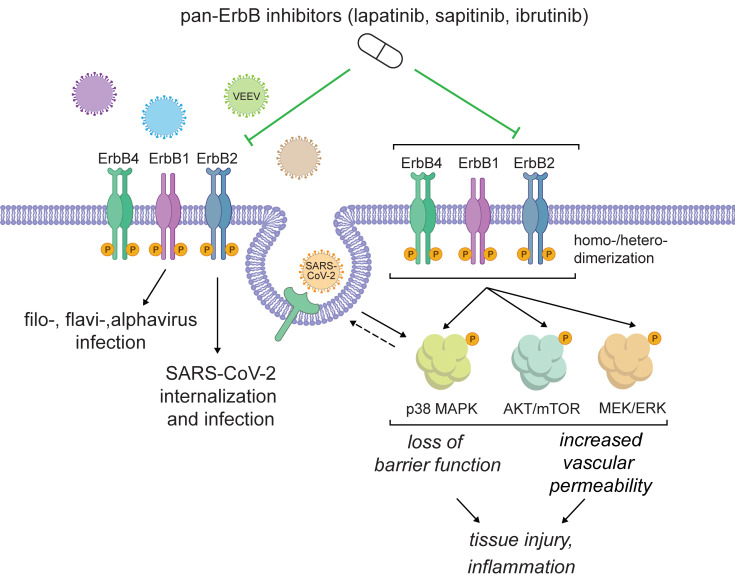

## Introduction

Acute emerging viral infections can cause epidemics and pandemics and thus pose major threats to human health. Severe acute respiratory syndrome coronavirus 2 (SARS-CoV-2) has spread globally, causing largely asymptomatic or mild infections, yet progressing in some patients to severe coronavirus disease 2019 (COVID-19) manifesting with acute lung injury, acute respiratory distress syndrome, and lung fibrosis (1). Venezuelan equine encephalitis virus (VEEV), an alphavirus naturally transmitted by mosquitoes, causes encephalitis associated with neurological deficits in up to 14% of infected individuals (2). The widespread mosquito-borne flavivirus dengue (DENV) and the filoviruses Ebola (EBOV) and Marburg (MARV) are causative agents of outbreaks of hemorrhagic fever. Monkeypox (MPOXV) is the causative agent of a current outbreak (3). Retaining infectivity as aerosols and/or droplets, VEEV, filoviruses, and MPOXV are considered bioterrorism threats (2, 4). The current lack of effective intervention strategies against the majority of emerging viruses leaves the global and military populations unprepared for future pandemics.

By targeting viral enzymes, most approved antivirals to date provide narrow-spectrum coverage. This approach has shown utility in treating chronic viral infections, such as hepatitis C virus, and, more recently, COVID-19. Nevertheless, the rapid rollout of direct-acting antivirals (DAAs) for COVID-19 treatment was enabled either via repurposing — remdesivir and molnupiravir, developed as anti-EBOV and anti-influenza agents, respectively — or via derivatization of existing SARS-CoV-1 main protease (Mpro) inhibitor in the case of nirmatrelvir. No such DAAs are available, however, for most viral families (5). Moreover, targeting viral factors by monotherapy often results in rapid emergence of drug resistance (5). Indeed, escape mutations conferring resistance to remdesivir and nirmatrelvir have already been selected in vitro and identified in circulating SARS-CoV-2 strains (6).

There is thus an unmet need for additional approaches, ideally targeting distinct mechanisms, to be used individually or in combination drug treatment for preventing the acute and long-term complications associated with viral infections and providing readiness for future outbreaks. Targeting host factors commonly required by multiple viruses is an alternative antiviral approach that could provide broad-spectrum coverage while increasing the barrier to resistance (5, 7). The opportunity to repurpose existing drugs known to modulate specific host functions with favorable toxicity profiles is attractive, particularly for the treatment of emerging viral infections lacking any treatment.

To address these gaps, we conducted a high-throughput screen of existing compounds for agents that rescue mammalian cells from SARS-CoV-2–induced lethality. Among the hits were inhibitors of members of the epidermal growth factor receptor (ErbB) family of receptor tyrosine kinases, including lapatinib, an approved anticancer drug. Here, we reveal that ErbBs regulate both the life cycle and pathogenesis of SARS-CoV-2 and VEEV infections. Moreover, we provide support for the feasibility of repurposing pan-ErbB inhibitors as a candidate broad-spectrum antiviral, antiinflammatory, and tissue-protective approach using in vitro and unique ex vivo models of multiple unrelated viral infections and a murine model of VEEV. Lastly, we characterize the mechanism of action of lapatinib and validate ErbBs as critical mediators of the antiviral effect.

## Results

### Pan-ErbB inhibitors emerge in a high-throughput screening for compounds that counteract SARS-CoV-2–induced lethality.

**Figure 1:**
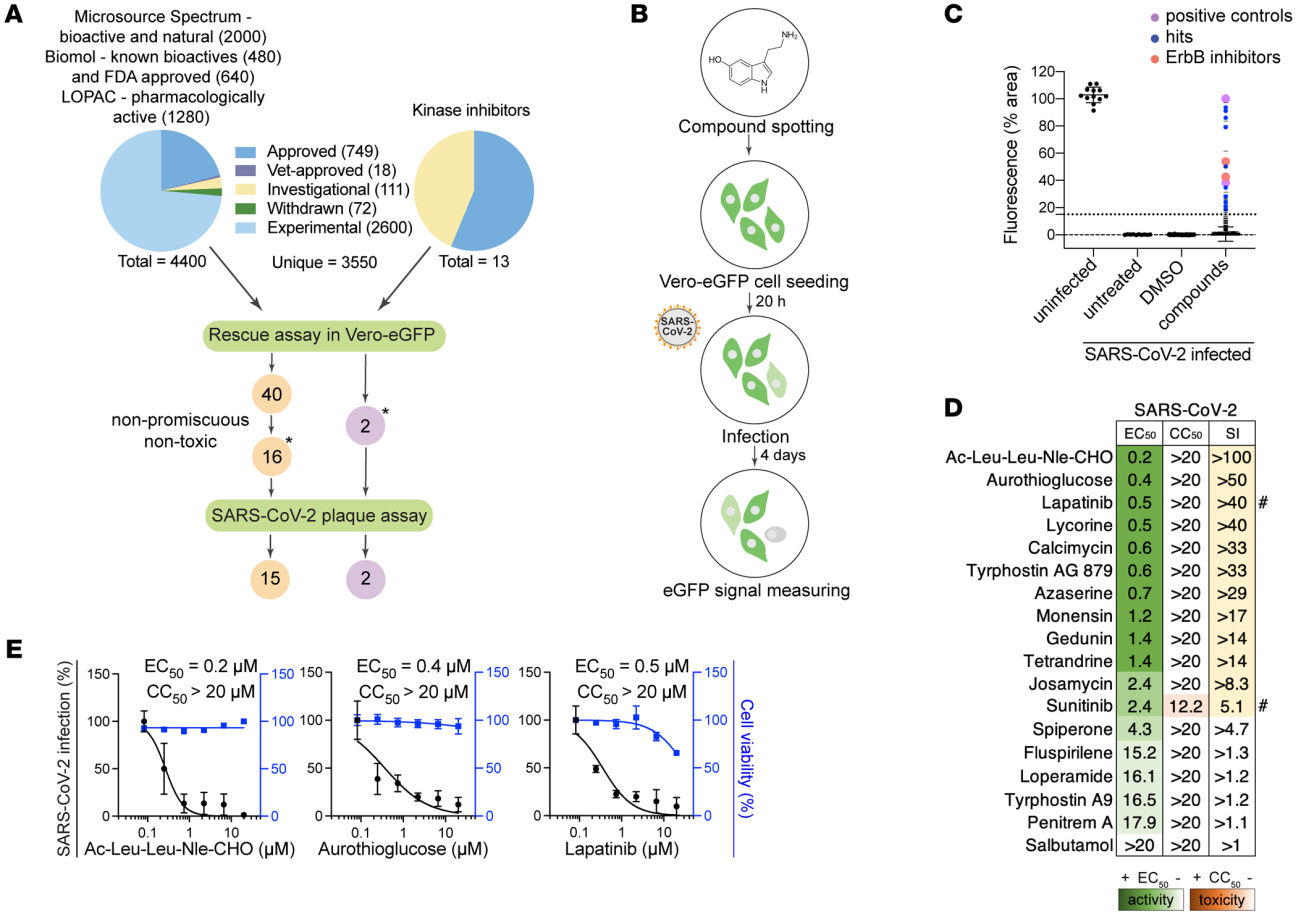
High-throughput screening for compounds that counteract SARS-CoV-2–induced lethality and validation by plaque assays. (**A**) Schematic of the composition of the screened libraries and screening and hit selection pipeline. LOPAC, Library of Pharmacologically Active Compounds (Sigma-Aldrich). (**B**) High-throughput screening (HTS) assay schematic. Compounds were pre-spotted in 384-well plates at a final concentration of 10 μM and incubated with Vero E6 cells constitutively expressing eGFP for 20 hours, followed by SARS-CoV-2 infection (Belgium-GHB-03021, MOI = 0.001). eGFP signal measured at 4 days after infection was used as an indicator for survival from virus-induced lethality. (**C**) Box plots of the percentage of fluorescence area values combining the entire HTS data set (2 independent experiments) split into the 4 indicated categories. The box horizontal lines indicate the first, second (median), and third quartiles. Outliers above a cutoff of 15% were defined as positive hits. Dots represent individual compounds, and colors denote positive controls (purple), new hits (blue), and ErbB inhibitors (peach). (**D**) Heatmap of the EC_50_ and CC_50_ values of hits emerging in the HTS, color-coded based on the antiviral activity measured by plaque assays (green) and toxicity measured by alamarBlue assays (orange), 24 hours after infection of Vero cells with SARS-CoV-2 (USA-WA1/2020 strain, MOI = 0.05). Selectivity indices (SI) greater than 5 are depicted in yellow. “#” indicates compounds from the 13-kinase set. (**E**) Dose-response curves of representative hits depicting SARS-CoV-2 infection (black) and cell viability (blue). Data are relative to DMSO. Data in **E** are combined from 2 independent experiments, each with 2–3 biological replicates. Means ± SD are shown. Asterisks in **A** denote 18 hits screened for SARS-CoV-2, VEEV (TC-83), and DENV2.

We assembled a collection of 4,413 bioactive investigational and FDA-approved compounds derived from 4 libraries and a self-assembled set of 13 kinase inhibitors (Figure 1A and Supplemental Figure 1A; supplemental material available online with this article; https://doi.org/10.1172/JCI169510DS1). This collection was screened in 2 independent experiments for inhibition of lethality induced by SARS-CoV-2 (isolate: Belgium-GHB-03021) infection in Vero E6 cells constitutively expressing enhanced green fluorescent protein (eGFP) via a high-throughput assay (8) (Figure 1B) that demonstrated robustness and specificity (Supplemental Text 1 and Supplemental Figure 1, B–D). We set a percentage fluorescent area of greater than 15 in at least one of the screens as the cutoff for positive hits (Figure 1C and Supplemental Figure 1, B–E). Forty-two compounds met this criterion, including nelfinavir and salinomycin, which have previously demonstrated anti–SARS-CoV-2 activity (9), met this criterion. Eighteen of the 42 hits were prioritized based on PubChem data documenting lower promiscuity and toxicity or activity against other viruses (Figure 1A) and then assessed for their effect on SARS-CoV-2 infection and cellular viability in Vero cells infected with a distinct viral isolate (2019-nCoV/USA-WA1/2020) via plaque and alamarBlue assays, respectively. Seventeen hits demonstrated antiviral effect beyond toxicity, of which 7 showed potent dose-dependent antiviral activity with half-maximal effective concentration (EC_50_) less than 0.7 μM, half-maximal cellular cytotoxicity (CC_50_) greater than 20 μM, and selectivity index (CC_50_ to EC_50_ ratio) greater than 20. These compounds target diverse cellular factors and functions (Figure 1, D and E, Table 1, and Supplemental Figure 2A). Two of these hits were reported to target ErbBs: lapatinib and tyrphostin AG879 (10). Inhibitors of numb-associated kinases (NAKs), heat shock protein 90 (HSP90), and ion transport were also among the hits.

### Lapatinib inhibits SARS-CoV-2 infection in vitro and ex vivo in human adult lung organoid–derived monolayers and is highly synergistic with nirmatrelvir.

**Figure 2:**
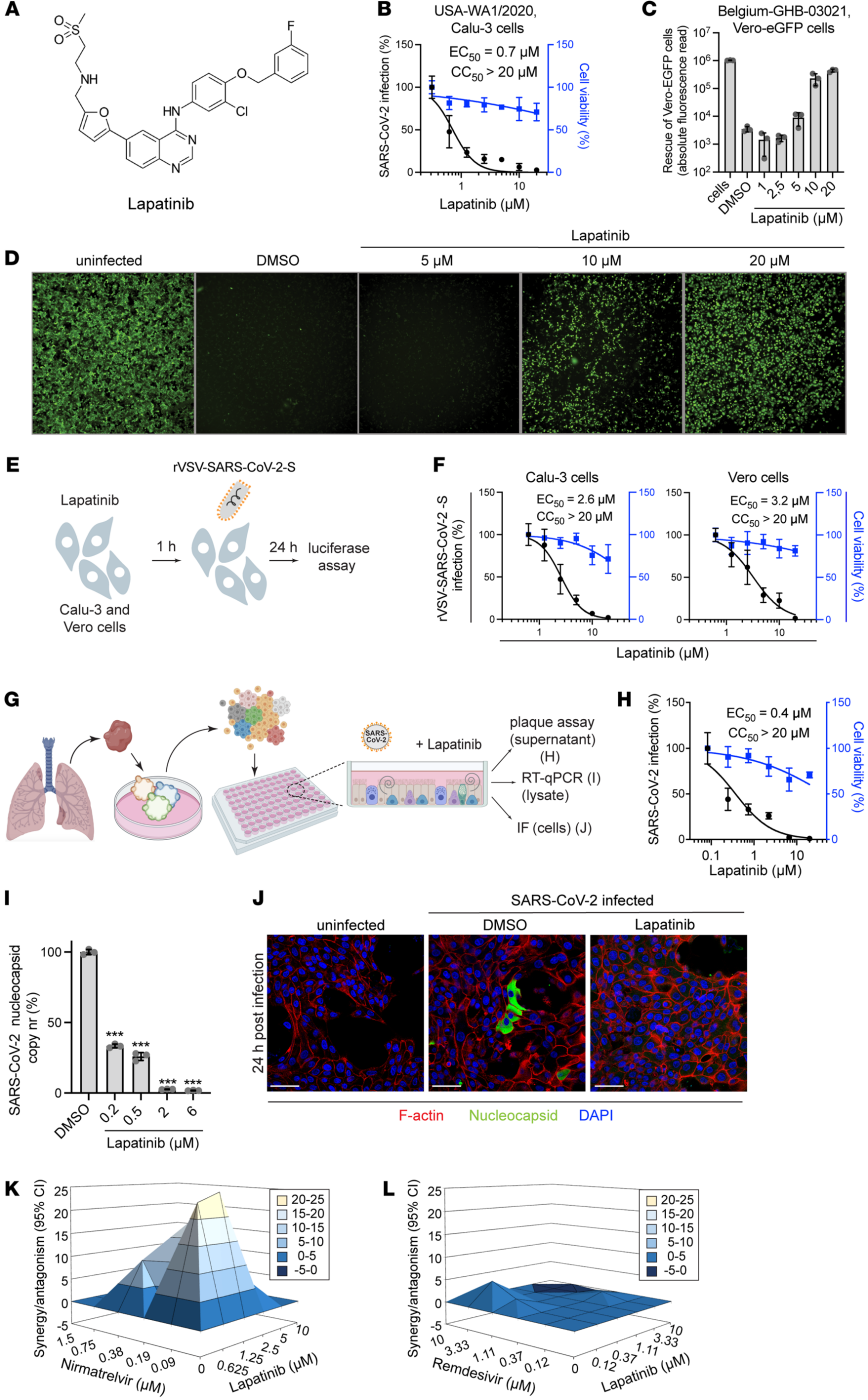
Lapatinib inhibits SARS-CoV-2 infection in vitro and ex vivo and is synergistic with nirmatrelvir. (**A**) Chemical structure of lapatinib. (**B**) Dose response to lapatinib of SARS-CoV-2 infection (black, USA-WA1/2020 strain, MOI = 0.05) and cell viability (blue) in Calu-3 cells via plaque and alamarBlue assays at 24 hpi, respectively. (**C** and **D**) Dose-dependent graph (**C**) and corresponding fluorescence images (**D**) of Vero-eGFP cells rescued from SARS-CoV-2–induced lethality by lapatinib at 96 hpi (Belgium-GHB-03021 strain, MOI = 0.05). Original magnification, ×5. (**E**) Schematic of the experiment shown in **F**. (**F**) Dose response to lapatinib of rVSV-SARS-CoV-2-S infection (black) and cell viability (blue) in Calu-3 and Vero cells via luciferase and alamarBlue assays at 24 hpi. (**G**) Schematic of ALO model and experimental procedures. ALO-derived monolayers were infected with SARS-CoV-2 (USA-WA1/2020 strain, MOI = 1). (**H**) Dose response to lapatinib of SARS-CoV-2 infection (black) and cell viability (blue) in ALO-derived monolayer supernatants via plaque and alamarBlue assays at 48 hpi. (**I**) Dose response to lapatinib of SARS-CoV-2 nucleocapsid copy number in ALO-derived monolayer lysates measured by RT-qPCR assays at 48 hpi. (**J**) Confocal IF microscopy images of F-actin (red), SARS-CoV-2 nucleocapsid (green), and DAPI (blue) in naive and SARS-CoV-2–infected ALO-derived monolayers pretreated with DMSO or 10 μM lapatinib 24 hpi. Representative merged images at ×40 magnification are shown. Scale bars: 50 μm. (**K** and **L**) Synergy/antagonism of combination treatment with lapatinib and nirmatrelvir (**K**) or remdesivir (**L**) on antiviral effect measured in Calu-3 cells infected with rSARS-CoV-2/Nluc (USA-WA1/2020 strain, MOI = 0.05) at 24 hpi via Nluc assays. Data represent differential surface analysis at the 95% confidence interval (MacSynergy II program). Data are representative (**C**, **H**, **I**, **K**, and **L**) or a combination (**B** and **F**) of 2 independent experiments with 2–3 replicates each. Data in **B**, **F**, **H**, and **I** are relative to DMSO. Means ± SD are shown. ***P < 0.001 by 1-way ANOVA followed by Dunnett’s multiple-comparison test.

Since lapatinib is an approved, oral pan-ErbB inhibitor, we focused on defining its antiviral potential. Similarly to its effect in Vero cells (EC_50_ = 0.5 μM, CC_50_ > 20 μM) (Figure 1E), in Calu-3 cells (human lung epithelial cells), lapatinib dose-dependently inhibited replication of SARS-CoV-2 (USA-WA1/2020 strain) as measured via plaque assay (EC_50_ = 0.7 μM), without apparent effect on cellular viability at the concentrations used (CC_50_ > 20 μM) (Figure 1E and Figure 2, A and B). Moreover, lapatinib demonstrated a dose-dependent rescue of Vero-eGFP cells from SARS-CoV-2–induced lethality (Figure 2, C and D). Likewise, lapatinib treatment dose-dependently suppressed infection of Calu-3 and Vero cells with replication-restricted pseudovirus bearing SARS-CoV-2 spike (S) protein (rVSV-SARS-CoV-2-S) as measured by luciferase assays (EC_50_ = 2.6–3.2 μM, CC_50_ > 20 μM) (Figure 2, E and F), suggesting that lapatinib inhibits viral entry.

To study the effect of lapatinib treatment on SARS-CoV-2 infection in a more biologically relevant model, we used a validated human adult lung organoid–derived (ALO-derived) monolayer model. Generated from adult stem cells isolated from lung tissue, these organoid-derived monolayers contain airway cells, critical for sustained viral infection, and alveolar cells, required for mounting the overzealous immune response in fatal COVID-19 (11) (Figure 2G). Viral replication measured by plaque assays in culture supernatant and nucleocapsid transcript expression measured by quantitative reverse transcription PCR (RT-qPCR) in ALO-derived monolayer lysates both peaked at 48 hours after SARS-CoV-2 infection (Supplemental Figure 2, B and C) and were dose-dependently suppressed by lapatinib, with EC_50_ values of 0.4 μM and <0.2 μM, respectively, and CC_50_ greater than 20 μM (Figure 2, H and I). Confocal immunofluorescence (IF) analysis revealed a near-complete disappearance of SARS-CoV-2 nucleocapsid staining in ALO-derived monolayers treated with lapatinib relative to DMSO (Figure 2J and Supplemental Figure 2D).

To determine the utility of lapatinib in combination treatment, we measured the anti–SARS-CoV-2 activity of combinations of lapatinib with clinically used DAAs. Lapatinib-nirmatrelvir combinations exhibited synergistic inhibition of SARS-CoV-2 infection as measured via luciferase assay with a synergy volume of 91.42 μM^2^% (within a range of moderate to important in vivo; ref. 12) and antagonism volume of 0 μM^2^% at the 95% confidence interval (MacSynergy; ref. 12) (Figure 2K). In contrast, lapatinib-remdesivir combinations were additive (Figure 2L). No synergistic toxicity was measured with these combinations (Supplemental Figure 2, E and F).

These results point to lapatinib as a potent anti–SARS-CoV-2 inhibitor with potential utility in combination drug treatment with Paxlovid.

### Lapatinib demonstrates broad-spectrum antiviral activity and high genetic barrier to resistance.

**Figure 3:**
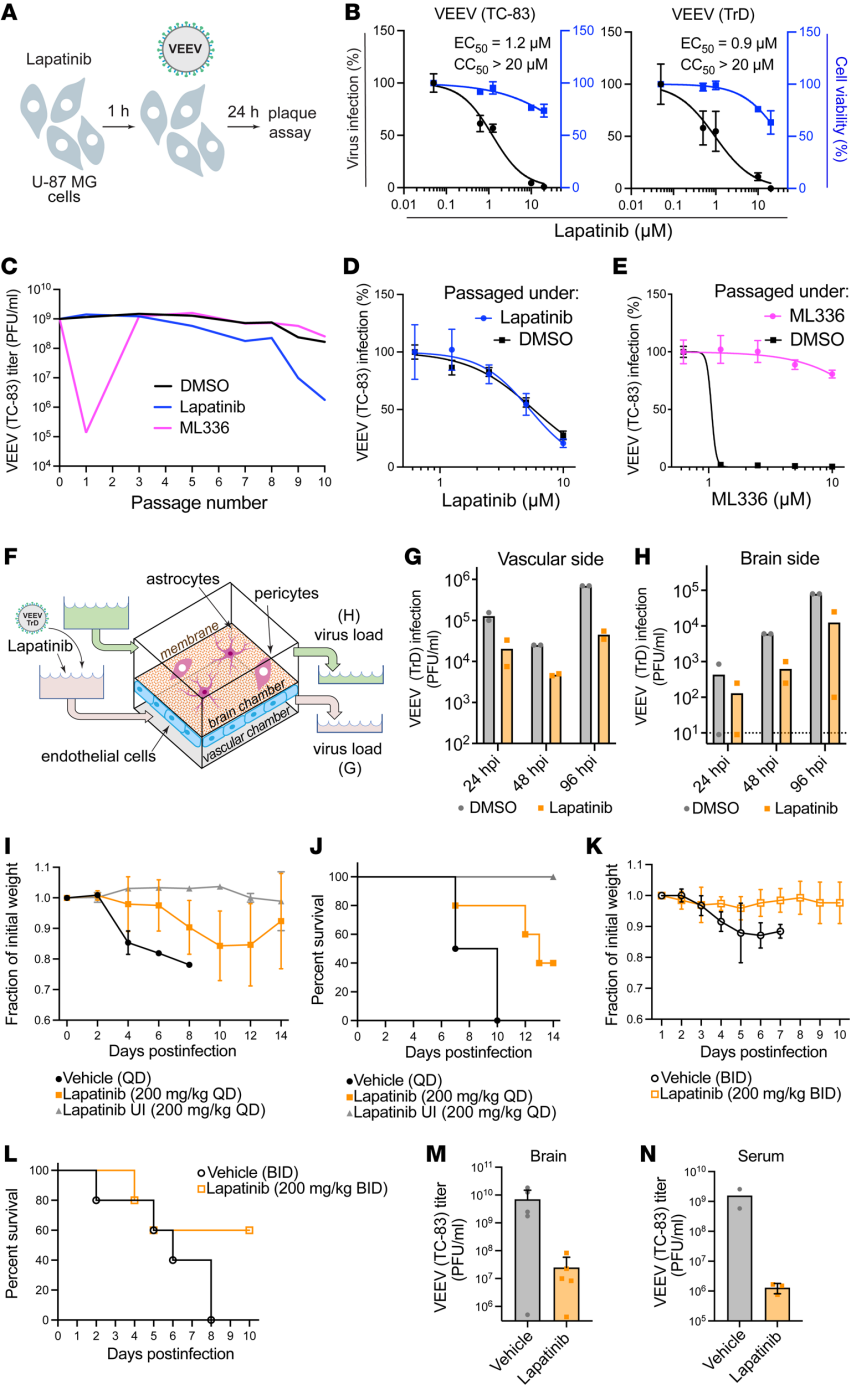
Lapatinib is a potent broad-spectrum antiviral with a high genetic barrier to resistance and is protective in human gNVU and murine models of VEEV. (**A**) Schematic of the experiment shown in **B**. (**B**) Dose response to lapatinib of infection with vaccine (TC-83) and WT (TrD) VEEV strains (MOI = 0.1) in U-87 MG cells via plaque and alamarBlue assays at 24 hpi, respectively. (**C**) VEEV (TC-83) was used to infect U-87 MG cells (MOI = 0.1) and then passaged every 24 hours by inoculation of naive cells with equal volumes of supernatants under DMSO treatment or selection with lapatinib or ML336 increasing from 2.5 to 15 μM over 10 passages. Viral titers were measured by plaque assays. (**D** and **E**) Dose response to lapatinib (**D**) and ML336 (**E**) of VEEV (TC-83) harvested after 10 passages in the presence of lapatinib (**D**) and ML336 (**E**) via luciferase assays. (**F**) Schematic of gNVU. (**G** and **H**) Viral load in longitudinal samples collected from the vascular (**G**) and brain (**H**) sides of the gNVU after infection with VEEV (TrD) and treatment with lapatinib or DMSO. (**I**–**L**) Weight loss (**I** and **K**) and mortality (**J** and **L**) of VEEV (TC-83)–infected C3H/HeN mice treated once (**I** and **J**) or twice (**K** and **L**) daily for 14 (**I** and **J**) or 10 (**K** and **L**) days with vehicle or lapatinib (200 mg/kg) (*n* = 2–5 per group). (**M** and **N**) Viral titers in brain (**M**) and serum (**N**) samples obtained upon euthanasia for morbidity or at the end of the experiment from mice treated twice daily (*n* = 2–5 per group). In **N**, day 8 (vehicle) and day 10 (lapatinib) titers were compared. Data in **B**, **D**, and **E** are relative to DMSO. Data are representative (**C**, **D**, **E**, **G**, and **H**) or a combination (**B**) of 2 independent experiments with 2–3 replicates each. See another independent experiment associated with **G** and **H** in Supplemental Figure 4E. Means ± SD are shown. QD, once daily; BID, twice daily; UI, uninfected.

We studied the broad-spectrum potential of lapatinib and the other 17 high-throughput screening (HTS) hits (Supplemental Text 2 and Supplemental Figure 3, A and B). Lapatinib dose-dependently inhibited alphavirus replication of both vaccine (TC-83) and wild-type (WT) (Trinidad donkey [TrD]) VEEV by plaque assays in human astrocyte (U-87 MG) cells (EC_50_ = 1.2 μM and 0.9 μM, respectively, and CC_50_ > 20 μM) (Figure 3, A and B). Similarly, lapatinib dose-dependently inhibited the replication of the flavivirus DENV2 (EC_50_ = 2.0 μM) via plaque assays, and the filoviruses EBOV (EC_50_ = 2.5 μM) and MARV (EC_50_ = 2.0 μM) via microneutralization assays, in human hepatoma (Huh7) cells, albeit lower CC_50_ values were measured in infected Huh7 cells (10–11.5 μM) than in other cell lines. Lapatinib dose-dependently inhibited the replication of 2 strains of monkeypox, a dsDNA virus, in A549 cells (EC_50_ = 6.2–7.6 μM) (Supplemental Figure 4, A–D).

To determine whether viruses can escape treatment with lapatinib, VEEV (TC-83) was passaged in U-87 MG cells in the presence of lapatinib or the VEEV nonstructural protein 2 (nsP2) inhibitor ML336 (13) at increasing concentrations corresponding to values between the EC_50_ and EC_90_, and viral titers were measured in culture supernatants by plaque assays. By passage 3, VEEV overcame inhibition by ML336. In contrast, VEEV remained suppressed for 10 passages under lapatinib treatment without phenotypic resistance (Figure 3C). Moreover, virus obtained from culture supernatants at passage 10 under lapatinib or DMSO treatment remained susceptible to lapatinib (Figure 3D). Conversely, virus obtained at passage 10 under ML336 treatment lost its susceptibility to ML336, with the emergence of a characterized resistance mutation in nsP2 (Y102C in VEEV TC-83), whereas virus obtained at the same passage under DMSO treatment retained ML336 susceptibility (Figure 3E).

These results support that lapatinib suppresses viral infection by targeting a cellular function and point to lapatinib as a potential broad-spectrum antiviral agent with a higher relative barrier to resistance than a DAA.

### Lapatinib suppresses VEEV replication in an organ-on-a-chip human neurovascular unit model and protects mice from VEEV challenge.

Since blood-brain barrier (BBB) disruption contributes to encephalitic outcomes in VEEV infection (14), we studied the effect of lapatinib treatment on BBB integrity following VEEV infection in a validated gravity-flow neurovascular unit (gNVU) model (15, 16). The gNVU — composed of human primary brain endothelial cells on one side of a membrane and astrocytes and pericytes on the other so as to establish brain and vascular chambers — recreates the dynamic of multicellular BBB microenvironment (16). Lapatinib- or DMSO-containing culture medium was perfused an hour before introduction of TrD-containing medium into the vascular inlet of the gNVU (Figure 3F). Lapatinib treatment (5 μM) suppressed VEEV (TrD) replication in both the vascular and brain sides of the gNVU, as measured by plaque assays in perfused media at various time points after infection (Figure 3, G and H, and Supplemental Figure 4E).

To further address the therapeutic potential of lapatinib as an antiviral agent, we tested its application in a murine model of VEEV (TC-83). C3H/HeN mice were infected intranasally with a lethal infectious dose of VEEV (TC-83) inoculum (5 × 10^6^ PFU). Once-daily treatment with 200 mg/kg lapatinib or vehicle alone via oral gavage was initiated at 12 hours before inoculation. The dose tested was lower than the approved human dose, as calculated based on the body surface area (17), and the maximum tolerated dose in mice (18, 19) and confirmed to be nontoxic in our VEEV model. The animals were monitored twice daily and euthanized when moribund. During a 14-day drug treatment, we observed reduced morbidity and mortality of infected animals relative to vehicle controls (Figure 3, I and J). Whereas 100% of vehicle-treated mice succumbed to infection by day 10 after infection, lapatinib treatment protected 80% of the mice by day 10, and 40% of the mice by day 14.

Since lapatinib’s half-life is shorter in mice than in humans, we administered lapatinib twice daily (200 mg/kg) for 8 days in uninfected C57BL/6 mice, observing good tolerability and plasma concentrations exceeding the EC_50_ deduced from our in vitro data by 6- to 43-fold (Supplemental Figure 4F). Despite the earlier mortality of vehicle-treated mice observed in this independent experiment (100% succumbed to infection by day 8 after infection), treatment with 200 mg/kg lapatinib twice daily for 10 days in the VEEV (TC-83) murine model protected 60% of the mice (Figure 3, K and L). Measurement of viral burden in serum and brain by plaque assays revealed a 2.5- to 3-log reduction of the infectious virus load in mice treated twice daily with lapatinib relative to vehicle controls (Figure 3, M and N).

These results demonstrate therapeutic potential of lapatinib in biologically relevant models against infections with at least 2 unrelated emerging RNA viruses.

### ErbBs are essential for SARS-CoV-2 and VEEV infections.

**Figure 4:**
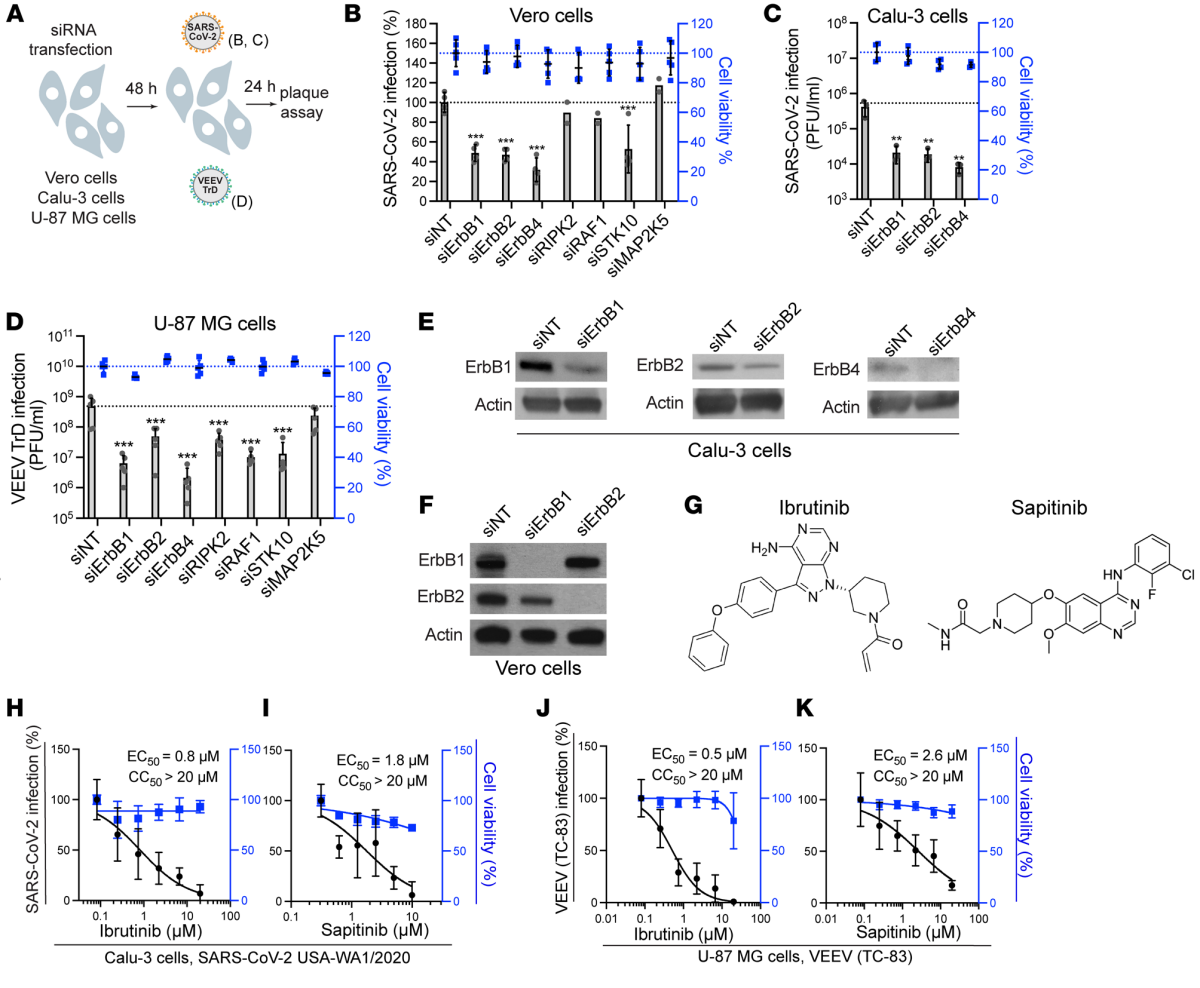
ErbBs are essential for SARS-CoV-2 and VEEV infections. (**A**) Schematic of the experiment shown in **B**–**D**. (**B**) Percentage of infection by plaque assays (gray) and cell viability by alamarBlue assays (blue) in Vero cells transfected with the indicated siRNA pools measured at 24 hours after infection with WT SARS-CoV-2. (**C** and **D**) Viral titers (gray) and cell viability (blue) in Calu-3 (**C**) and U-87 MG (**D**) cells transfected with the indicated siRNA pools measured at 24 hours after infection with WT SARS-CoV-2 (**C**) or VEEV (TrD) (**D**). (**E** and **F**) Confirmation of siRNA-mediated gene expression knockdown in Calu-3 (**E**) and Vero (**F**) cells at 48 hours after transfection by Western blot. Notably, 2 anti-ErbB4 antibodies detected no signal of endogenous protein in Vero cells. (**G**) Chemical structures of ibrutinib and sapitinib. (**H**–**K**) Dose response to ibrutinib (**H** and **J**) and sapitinib (**I** and **K**) of SARS-CoV-2 (black, USA-WA1/2020 strain, MOI = 0.05) (**H** and **I**) and VEEV (TC-83) (**J** and **K**) infection by plaque assays and cell viability (blue) by alamarBlue assays at 24 hours after infection of Calu-3 (**H** and **I**) or U-87 MG (**J** and **K**) cells. Data are representative (**C**) or a combination (**B**, **D**, and **H**–**K**) of 2 independent experiments with 2–3 replicates each. Means ± SD are shown. Data are relative to DMSO (**H**–**K**) or siNT (**B**–**D**). **P = 0.003, ***P < 0.001 by 1-way ANOVA followed by Dunnett’s multiple-comparison test.

Three of the 4 ErbB family members — ErbB1, ErbB2, and ErbB4 — are catalytically active (20). Lapatinib’s cancer targets are ErbB1 (EGFR) (IC_50_ = 5.3 nM) and ErbB2 (HER2) (IC_50_ = 35 nM) (21), yet it was shown to comparably bind the ATP binding site of ErbB4 (22). Indeed, we measured an IC_50_ of 28 nM of lapatinib on ErbB4 in a cell-free assay and confirmed its anti-ErbB2 activity (Supplemental Figure 5A). Beyond ErbBs, lapatinib’s kinome (HMS LINCS database ID: 20107; https://lincs.hms.harvard.edu/db/) reveals binding to RAF1, STK10, RIPK2, and MAP2K5, with an overall excellent selectivity for ErbBs by *K_D_* measurements (HMS LINCS database ID: 20155). To define the molecular targets mediating the observed antiviral effect of lapatinib, we studied the effects of siRNA-mediated depletion of these 7 kinases in Vero E6 cells infected with WT SARS-CoV-2 via plaque assay (Figure 4, A and B). ErbB depletion suppressed SARS-CoV-2 replication by approximately 50% relative to a non-targeting (siNT) control. A similar phenotype was observed in Vero cells infected with pseudovirus (rVSV-SARS-CoV-2-S), revealing a role of ErbBs in viral entry, a stage of the SARS-CoV-2 life cycle that is inhibited by lapatinib (Supplemental Figure 5, B and C). In Calu-3 cells, depletion of ErbBs by these siRNA pools suppressed WT SARS-CoV-2 infection by 1.5 to 1.7 logs relative to siNT as measured via plaque assays (Figure 4, A and C). Silencing of ErbB expression in U-87 MG cells by these siRNAs suppressed TrD infection by 1 to 2 logs and VEEV (TC-83) infection by 30%–70% relative to siNT, as measured via plaque assays (Figure 4, A and D, and Supplemental Figure 5, D and E). ErbB depletion by the siRNA pools did not impact cell viability (Figure 4, B–D, and Supplemental Figure 5, C and E), and its efficiency was confirmed by Western blot and RT-qPCR analysis (Figure 4, E and F, and Supplemental Figure 5F). STK10 depletion inhibited both SARS-CoV-2 and TrD infections, whereas RIPK2 and RAF1 depletion suppressed only TrD infection (Figure 4, B and D).

To further probe the requirement for ErbBs in SARS-CoV-2 and VEEV infections, we evaluated the antiviral effect of 2 chemically distinct compounds: ibrutinib, an approved anticancer Bruton’s tyrosine kinase (BTK) inhibitor, and sapitinib (investigational), both with potent pan-ErbB activity (21, 23) (Figure 4G, Supplemental Figure 5A, and Table 2). These compounds suppressed SARS-CoV-2 and VEEV (TC-83) infections, with EC_50_ values at sub- to low micromolar range and CC_50_ greater than 20 μM (Figure 4, H–K).

These findings provide genetic and pharmacological evidence that ErbBs are required for SARS-CoV-2 and VEEV infections, thereby validating them as druggable antiviral targets.

### ErbBs bind the viral spike S1 subunit, and their inhibition suppresses SARS-CoV-2 internalization.

**Figure 5:**
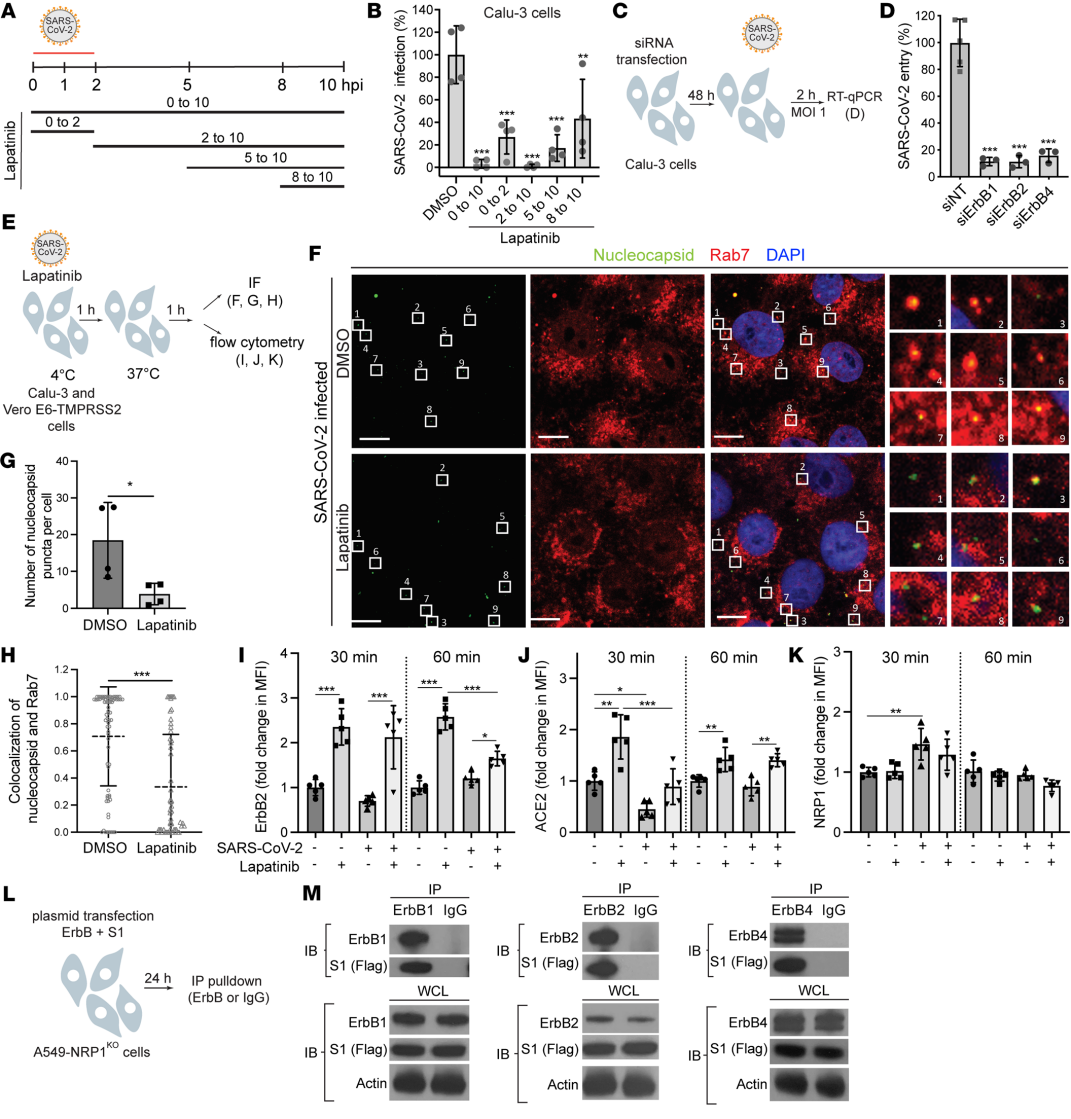
ErbBs bind the viral spike S1 subunit, and their inhibition suppresses SARS-CoV-2 and ACE2 internalization. (**A**) Schematic of the time-of-addition experiment shown in **B**. (**B**) Calu-3 cells were infected with SARS-CoV-2 (MOI = 1). At the indicated times, 10 μM lapatinib or DMSO was added. Supernatants were collected at 10 hpi, and viral titers were measured by plaque assay. Values are shown relative to DMSO control. (**C**) Schematic of the experiment shown in **D**. (**D**) WT SARS-CoV-2 entry at 2 hpi in Calu-3 cells (MOI = 1) depleted of the indicated ErbBs measured by RT-qPCR. (**E**) Schematic of the experiments shown in **F**–**K**. (**F**–**H**) Quantitative IF analysis of SARS-CoV-2 internalization. Vero-TMPRSS2 cells were pretreated with lapatinib (10 μM) or DMSO and infected with SARS-CoV-2 (MOI = 1) at 4°C for 1 hour followed by temperature shift to 37°C. At 1 hpi, cells were fixed and labeled with nucleocapsid (green) and Rab7 (red) antibodies. The right panel shows the numbered areas magnified 6-fold. Scale bars: 10 μm. (**G**) Number of nucleocapsid puncta per cell after DMSO and lapatinib treatment. (**H**) Scatter plots of colocalization of nucleocapsid and Rab7 quantified by Manders’ coefficient. Dots represent individual viral particles; horizontal lines indicate means ± SD (DMSO: *n* = 71; lapatinib: *n* = 53). (**I**–**K**) Flow cytometry data of cell surface expression levels of ErbB2 (**I**), ACE2 (**J**), and NRP1 (**K**) at 30 and 60 minutes after temperature shift to 37°C in uninfected and SARS-CoV-2–infected Calu-3 cells treated with lapatinib or DMSO. Fold change in MFI is relative to 30-minutes uninfected DMSO-treated cells. (**L**) Schematic of the experiment shown in **M**. (**M**) A549-NRP1^KO^ cells were cotransfected with plasmids expressing S1-FLAG and ErbBs, followed by immunoprecipitation using anti-ErbB or IgG antibodies and protein G Dynabeads. Representative Western blots of eluates and whole-cell lysates (WCL) are shown. Data are representative (**D**, **F**–**H**, and **M**) or a combination (**B** and **I**–**K**) of 2 independent experiments with 2–5 replicates each. Means ± SD are shown (**B**, **D**, and **G**–**K**). Data are relative to DMSO (**B** and **G**–**K**) or siNT (**D**). *P ≤ 0.05, **P < 0.01, ***P < 0.001 by 1-way ANOVA followed by Dunnett’s (**B** and **D**) or Tukey’s (**I**–**K**) multiple-comparison test or by unpaired, 2-tailed Student’s *t* test (**G** and **H**).

To better understand the mechanism of action underlying the anti–SARS-CoV-2 activity of lapatinib, we probed the steps of the viral life cycle inhibited by lapatinib via time-of-addition experiments. Lapatinib was added to Calu-3 cells upon infection or at 2, 5, or 8 hours after infection with WT SARS-CoV-2 (Figure 5A). Cell culture supernatants were harvested at 10 hours post-infection (hpi), which represented a single cycle of viral replication in Calu-3 cells, and infectious viral titers were quantified by plaque assays. Lapatinib treatment initiated upon infection onset and maintained throughout the 10-hour experiment (0 to 10) suppressed viral infection by 98% (Figure 5B). Lapatinib treatment during the first 2 hours of infection only (0 to 2) suppressed viral infection by 75%, confirming an effect on entry of WT SARS-CoV-2 (beyond rVSV-SARS-CoV-2-S; Figure 2F). Moreover, after extensive washing at 2 hpi (to remove viral inoculum), the addition of lapatinib at 2, 5, and 8 hpi suppressed viral infection by 99%, 84%, and 57%, respectively, indicating inhibition also at post-entry stages (Figure 5B).

To genetically confirm the role of ErbBs in the entry of WT SARS-CoV-2 (beyond rVSV-SARS-CoV-2-S; Supplemental Figure 5, B and C), Calu-3 cells depleted for the individual ErbBs by the corresponding siRNAs were infected with high-inoculum virus followed by quantification of intracellular viral RNA at 2 hpi by RT-qPCR. siErbB1, siErbB2, and siErbB4 suppressed SARS-CoV-2 entry by 84%–89% relative to siNT (Figure 5, C and D). The effect of single, double, and triple ErbB depletion on viral entry was comparable (Supplemental Figure 6, A and B), suggesting that the heterodimeric ErbB receptors act non-redundantly in mediating SARS-CoV-2 infection.

To distinguish between viral binding and post-binding events, rVSV-SARS-CoV-2-S was incubated with Vero cells for 2 hours at 4°C in the presence or absence of lapatinib or DMSO before infection initiation by temperature shift to 37°C (Supplemental Figure 6C). Lapatinib had comparable effects on rVSV-SARS-CoV-2-S infection when added upon or after virus binding to cells with no cellular toxicity (Supplemental Figure 6D), providing evidence for suppression at a post-binding step.

We next monitored the effect of lapatinib on single SARS-CoV-2 particle internalization in TMPRSS2-expressing Vero E6 cells fixed at 1 hpi and temperature shift to 37**°**C, labeled for viral particles and late endosomes with antibodies targeting the nucleocapsid and Rab7, respectively, and imaged by confocal microscopy. Quantitative IF analysis revealed that lapatinib significantly reduced the number of nucleocapsid puncta per cell and the colocalization of over 50 randomly chosen SARS-CoV-2 particles in each category to late endosomes, a cellular compartment into which SARS-CoV-2 internalizes (24), with mean Manders’ coefficients of 0.33 versus 0.71 in DMSO-treated cells (Figure 5, E–H). The majority of viral particles had an equivalent size based on fluorescence emissions, suggesting that single viral particles were imaged.

We tested the hypothesis that lapatinib alters the internalization of the SARS-CoV-2 receptor ACE2 and coreceptor neuropilin-1 (NRP1) (25, 26). The cell surface expression levels of ErbB2 and these receptors were measured by flow cytometry analysis of Calu-3 cells pretreated with lapatinib or DMSO, infected with SARS-CoV-2 (or mock) at 4**°**C, and extracellularly stained at 30 and 60 minutes after a temperature shift to 37°C (Figure 5, E and I–K, and Supplemental Figure 6, E and F). In uninfected cells, lapatinib caused a more than 2-fold increase in the cell surface level of ErbB2 at both time points (Figure 5I), in agreement with its reported effect on dimerization and internalization of ErbB complexes (beyond phosphorylation) (27). Interestingly, lapatinib had a similar effect, most prominently at 30 minutes after infection, on the surface level of ACE2, but not NRP1 (Figure 5, J and K). SARS-CoV-2 infection decreased ACE2 surface level at 30 minutes after temperature shift, suggesting that ACE2 internalization, previously shown to be caused by S1-ACE2 binding (28, 29), is induced during SARS-CoV-2 entry (Figure 5J). A trend toward reduced ErbB2 levels, albeit statistically nonsignificant, was also measured at the early time point after infection, whereas the level of cell surface NRP1 was increased upon infection (Figure 5, I and K). Lapatinib treatment reversed the effect of SARS-CoV-2 infection on the surface level of ACE2 (Figure 5J) and increased the surface level of ErB2, but not NRP1, in infected cells relative to DMSO (Figure 5, I and K), suggesting that the internalization of ACE2 but not NRP1 may be regulated by ErbBs.

To determine whether ErbBs interact with the receptor-binding domain of SARS-CoV-2 spike protein S1, we performed coimmunoprecipitation assays. Since S1 was shown to bind ACE2 and NRP1, we studied potential interactions of S1 with ErbBs in A549-NRP1^KO^ cells with intrinsic ACE2 deficiency and deletion of NRP1 by CRISPR/Cas9. A549-NRP1^KO^ cells were cotransfected with plasmids expressing FLAG-tagged S1 and the individual ErbBs (Figure 5L). Anti-ErbB1, -ErbB2, and -ErbB4 antibodies effectively pulled down the respective ErbB (–180 kDa), with which a –100 kDa protein corresponding to S1 was coimmunoprecipitated (Figure 5M). No background signal was demonstrated with control IgG, indicating specificity of the observed coimmunoprecipitation.

These results provide evidence that ErbBs regulate SARS-CoV-2 internalization and virus-induced ACE2 internalization, and that their direct, ACE2- and NRP1-independent, binding to the viral S1 subunit may be implicated in this process.

### ErbBs are the molecular targets mediating the antiviral effect of lapatinib.

**Figure 6:**
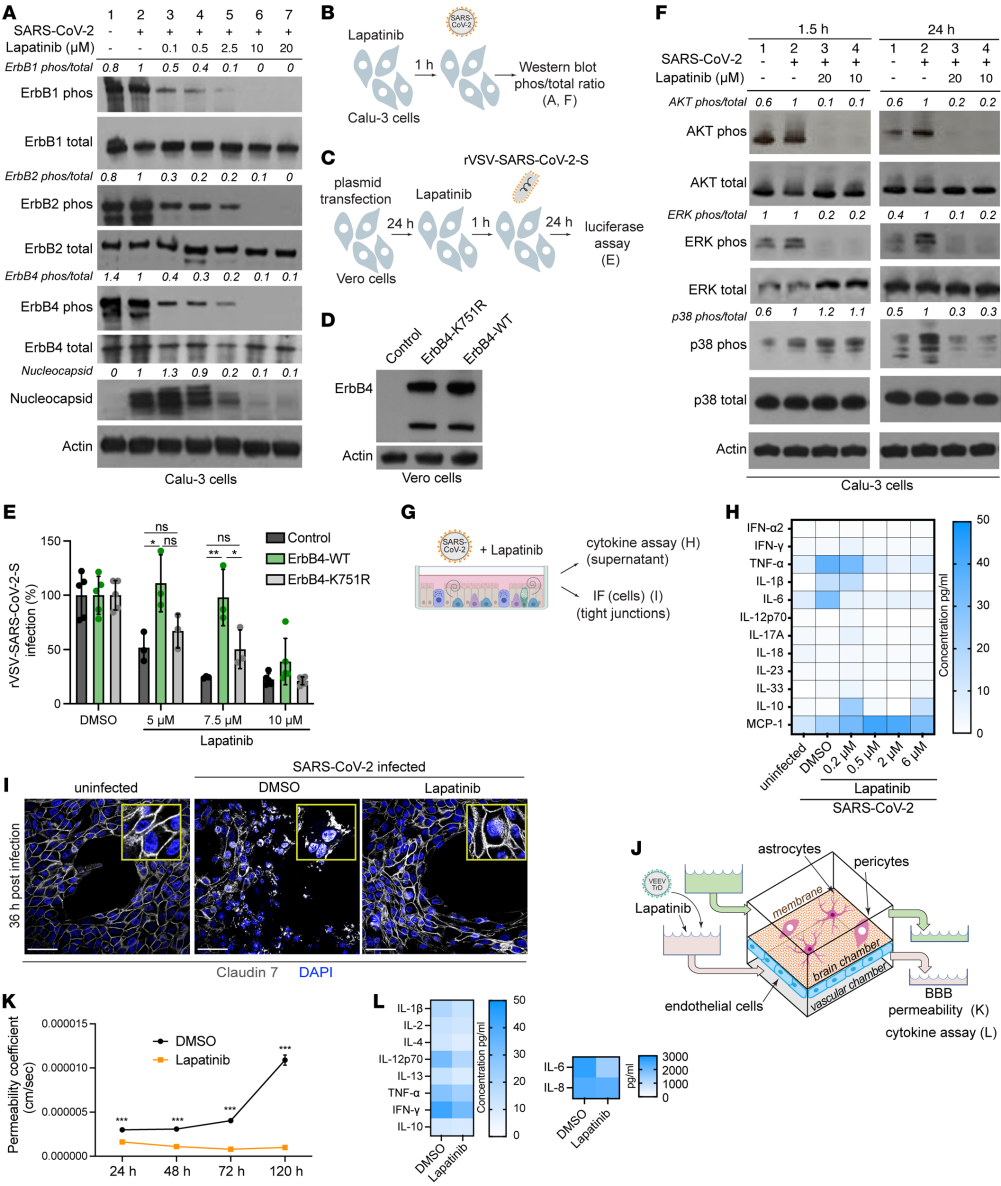
ErbBs are the molecular targets mediating the antiviral effect of lapatinib, and they regulate virus-induced inflammation and tissue injury. (**A** and **F**) ErbB (**A**), AKT, ERK, and p38 MAPK (**F**) phosphorylation and nucleocapsid expression (**A**) in Calu-3 cells that were uninfected (lane 1), infected and DMSO-treated (lane 2), or infected and lapatinib-treated (lanes 3–7) measured via Western blotting at 1.5 (**F**) and 24 (**A** and **F**) hours after infection with SARS-CoV-2 (USA-WA1/2020 strain, MOI = 1). Shown are representative membranes blotted for phospho- and total proteins and quantitative phospho- to total protein ratio data relative to infected cells treated with DMSO (lane 2). (**B**) Schematic of the experiment shown in **A** and **F**. (**C**) Schematic of the experiments shown in **D** and **E**. (**D**) Level of ErbB4 and actin expression via Western blot after transfection of Vero cells with control or ErbB4-expressing plasmids. (**E**) Rescue of rVSV-SARS-CoV-2-S infection in the presence of lapatinib upon ectopic expression of the indicated plasmids measured by luciferase assays at 24 hpi in Vero cells. (**G**) Schematic of the experiments shown in **H** and **I**. (**H**) Cytokine concentration (pg/mL) in ALOs’ supernatants at 48 hours after infection with SARS-CoV-2 by LEGENDplex kit. (**I**) Confocal IF microscopy images of claudin-7 (gray) and DAPI (blue) in naive or SARS-CoV-2–infected ALOs treated with DMSO or lapatinib (10 μM) and imaged at 36 hpi. Representative merged images at ×40 magnification are shown. Scale bars: 50 μm. (**J**) Schematic of the experiments shown in **K** and **L**. (**K**) Permeability of the endothelial layer of gNVUs infected with VEEV (TrD) and treated with lapatinib (5 μM) or DMSO assessed by FITC-dextran quantification in samples collected from brain and vascular chambers. (**L**) Cytokine concentration (pg/mL) in the brain side of gNVUs treated with lapatinib (5 μM) or DMSO at 120 hours after infection with VEEV (TrD) by LEGENDplex kit. Data are a combination (**E**) or representative (**A**, **D**, **F**, **H**, **I**, **K**, and **L**) of 2 independent experiments each with 2–4 replicates. Means ± SD are shown (**E** and **K**). *P < 0.05, **P < 0.01, ***P < 0.001 relative to DMSO by 1-way ANOVA followed by Tukey’s multiple-comparison test at each lapatinib concentration (**E**) or unpaired, 2-tailed Student’s *t* test (**K**).

To determine whether lapatinib exerts its antiviral effect by inhibiting phosphorylation of ErbBs, lysates from SARS-CoV-2–infected Calu-3 cells treated with lapatinib or DMSO were subjected to quantitative Western blot analysis of phospho-ErbB to total ErbB ratios. SARS-CoV-2 infection induced mild ErbB1 and ErbB2 phosphorylation in these cells. Lapatinib treatment dose-dependently suppressed the ratio of phosphorylated to total ErbB1, ErbB2, and ErbB4 at 24 hpi, with EC_50_ values lower than 0.1 μM, which correlated with reduced expression of the SARS-CoV-2 nucleocapsid protein (Figure 6, A and B). Analogous suppression of ErbB phosphorylation was measured in Calu-3 cells at 1.5 hpi and in ALO monolayers at 1.5 and 24 hpi (Supplemental Figure 7, A–C). These results provide evidence that drug exposure and the antiviral effect of lapatinib are correlated with functional inhibition of ErbBs’ activity.

To confirm that inhibition of ErbBs is a mechanism underlying the antiviral effect of lapatinib, we conducted gain-of-function “rescue” experiments in Vero cells infected with rVSV-SARS-CoV-2-S and U-87 MG cells infected with VEEV (TC-83). Ectopic expression of WT ErbB4, whose depletion suppressed both infections most prominently (Supplemental Figure 5, C and E), either completely or partially reversed the antiviral effect of various concentrations of lapatinib on rVSV-SARS-CoV-2-S and VEEV (TC-83) infections (Figure 6, C–E, and Supplemental Figure 7, D–H). In contrast, ectopic expression of a catalytically inactive ErbB4 mutant harboring a lysine to arginine substitution in position 751 (K751R) or control plasmid did not reverse the antiviral effect of lapatinib (Figure 6E and Supplemental Figure 7F). These findings validate ErbB4 as a mediator of lapatinib’s antiviral effect and indicate that its enzymatic activity is required for viral infection.

### ErbB inhibition suppresses virus-induced inflammation and tissue injury ex vivo in human ALO-derived monolayers and gNVUs.

Data from animal and human models of non-infectious acute lung injury and acute respiratory distress syndrome indicate that ErbB1 and ErbB2 are key regulators of inflammation and tissue injury via activation of the p38 MAPK, AKT/mTOR, and Ras/RAF/MEK/ERK pathways (20, 30–33). To test the hypothesis that these pathways are activated in SARS-CoV-2 infection and suppressed by lapatinib’s pan-ErbB inhibition, we measured their activation in Calu-3 cells upon SARS-CoV-2 infection and/or lapatinib treatment by Western blot analysis. At 1.5 and 24 hpi, SARS-CoV-2 increased the ratio of phosphorylated to total protein level of AKT, ERK, and/or p38 MAPK more than 1.5- to 2.5-fold (Figure 6, B and F), in agreement with reports in other cell lines (34, 35). Lapatinib treatment dramatically inhibited SARS-CoV-2–induced activation of AKT and ERK at both 1.5 and 24 hpi and of p38 MAPK at 24 hpi (Figure 6F). In the more complex ALO-derived monolayer model, lapatinib treatment inhibited SARS-CoV-2–induced phosphorylation of AKT and ERK, albeit not p38 MAPK (Supplemental Figure 7I).

To further test the hypothesis that ErbB-regulated signaling mediates the inflammatory response to SARS-CoV-2 infection, we measured cytokine levels in ALO-derived monolayer supernatants upon SARS-CoV-2 infection and treatment with lapatinib or DMSO. SARS-CoV-2 infection increased the production of TNF-α, IL-1β, and IL-6, in agreement with former reports (36). Lapatinib treatment dose-dependently reduced the expression level of these proinflammatory cytokines, with levels at or lower than those measured in uninfected ALO-derived monolayers achieved at 0.5 μM (Figure 6, G and H). Concurrently, lapatinib increased the expression level of MCP-1, suggesting that it may augment innate immune responses (37).

To define the role of ErbB signaling in SARS-CoV-2–induced lung injury, we analyzed the effect of lapatinib on the integrity of tight junction formation in ALO-derived monolayers via confocal IF analysis. Claudin-7 staining of uninfected ALO-derived monolayers revealed a continuous membranous pattern (Figure 6, G and I, and Supplemental Figure 7J). Thirty-six hours after SARS-CoV-2 infection and DMSO treatment, claudin-7 stained as speckles or short segments that often appeared in the cytoplasmic region. This finding was accompanied by cell separation and destruction of the alveolar-like architecture. In contrast, ALO-derived monolayers treated with lapatinib (10 μM) exhibited intact claudin-7 morphology and subcellular distribution as well as preserved architecture of the alveolar-like structure, comparable to uninfected controls (Figure 6I and Supplemental Figure 7J).

To determine whether these observations are generalizable to other viral infections, we monitored the effect of lapatinib on BBB integrity in VEEV (TrD)–infected gNVUs by quantifying the permeability of FITC-dextran every 24 hours for the total 120 hours. In TrD-infected, DMSO-treated gNVUs, the permeability dramatically increased starting at 72 hpi. Contrastingly, infected gNVUs treated with lapatinib (5 μM) maintained barrier integrity (Figure 6, J and K). In parallel, lapatinib treatment reduced the production of the proinflammatory cytokines IL-2, IL-12p70, IL-13, and IL-6, but not IFN-γ (antiviral) and IL-10 (immune suppressant), as measured via multiplexed ELISA in perfusion media from the brain compartment at 120 hpi (Figure 6, J and L).

**Figure 7:**
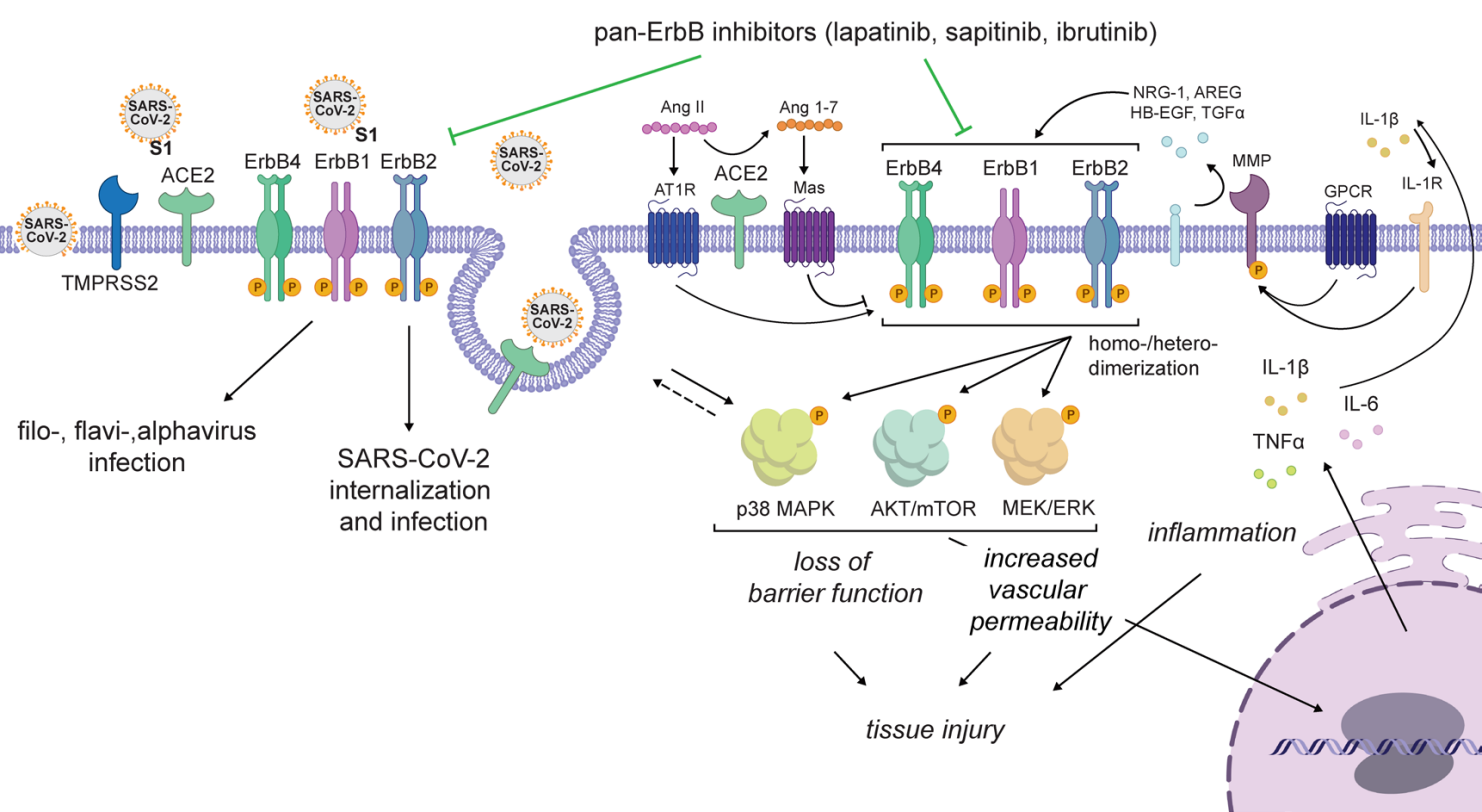
Proposed model for the roles of ErbBs in the regulation of viral infection and pathogenesis and the mechanism of action of pan-ErbB inhibitors. ErbBs regulate SARS-CoV-2 internalization and other stages of the viral life cycle and are required for effective replication of other emerging RNA viruses. Moreover, pan-ErbB activation promotes signaling in pathways implicated in inflammation and tissue injury in severe pandemic coronaviral infections and other disease models. By inhibiting ErbBs, lapatinib and other pan-ErbB inhibitors not only suppress viral infection but also protect from the resulting inflammation and tissue injury.

Based on our data and the cumulative published data, we propose a model wherein ErbBs are required for the life cycle of SARS-CoV-2 and VEEV, while pan-ErbB activation of downstream signaling pathways by these and other viruses mediates inflammation and tissue injury. By suppressing both processes, pan-ErbB inhibitors not only inhibit viral infection, but also protect from the resulting inflammation and the disruption of lung epithelium and BBB integrity (Figure 7). Whereas lapatinib’s antiinflammatory and antiviral effects cannot be decoupled based on our experiments, others have shown that lapatinib reverses increased epithelium permeability in a non-infectious model in vitro (30) and investigational ErbB inhibitors protect from acute and chronic lung injury in non-infectious models in vivo (31, 38–40). We therefore propose that lapatinib’s protective effect from inflammation and tissue injury is only partly driven by its antiviral effect; however, further validation is required.

## Discussion

While ErbB1 has been implicated in the life cycle of multiple RNA and DNA viruses (41), its precise role in coronavirus infections, its role in alphavirus infections, and the roles of ErB2 and ErbB4 in any viral infection remained unknown. Moreover, the relevance of ErbBs in virus-induced inflammation and acute tissue injury has not been reported. Here, we addressed this knowledge gap and studied the therapeutic potential of ErbB inhibition as a broad-spectrum antiviral strategy. Integrating virology, biochemical, genetic, immunological, and pharmacological approaches with ex vivo and in vivo models that recapitulate COVID-19 and VEEV pathology, we reveal regulation of SARS-CoV-2 and VEEV infections and subsequent inflammation and epithelial barrier or BBB injury by ErbBs, validating these kinases as attractive targets for antiviral therapy. Moreover, our findings provide a proof of concept for the utility of approved pan-ErbB inhibitors as broad-spectrum antiviral agents and reveal the mechanism of antiviral action and antiinflammatory and tissue-protective effects.

Using pan-ErbB inhibitors as pharmacological tools, we discover that ErbB1, ErbB2, and ErbB4 are required for effective SARS-CoV-2 and VEEV infections. Beyond pharmacologically, we genetically validate ErbBs as anti–SARS-CoV-2 and VEEV targets. We provide multiple lines of evidence to support modulation of ErbB activity as an important mechanism of lapatinib’s antiviral action. First, we show that lapatinib inhibits SARS-CoV-2 entry, analogous to the phenotype we reveal with RNAi-mediated suppression of ErbBs. Second, lapatinib’s antiviral activity correlates with reduced levels of phospho-ErbBs both in vitro and in ALO-derived monolayers. Third, WT, but not a kinase-dead ErbB4 mutant, reverses the anti–SARS-CoV-2 and anti-VEEV effect of lapatinib.

Our findings establish an effect of lapatinib on SARS-CoV-2 entry and provide insight into the roles of ErbBs in this stage of the viral life cycle. Specifically, lapatinib reduced the colocalization of SARS-CoV-2 particles with late endosomes at early time points after infection, revealing a role for ErbBs in viral internalization. Moreover, flow cytometry analysis revealed that lapatinib suppressed ACE2 internalization in uninfected cells and reversed the effect of SARS-CoV-2 infection on ACE2 but not NRP1 internalization. It is thus tempting to speculate that ErbBs regulate internalization of SARS-CoV-2 with ACE2. Indeed, there was a trend toward reduction of surface level expression of ErbB2 upon SARS-CoV-2 infection, suggesting that ErbB internalization may play a role in viral entry. Induction of ErbB signaling by host ligands promoting uptake events may be one mechanism regulating internalization of multiple viruses with their respective receptors. Our discovery that the SARS-CoV-2 spike S1 subunit binds ErbBs in the absence of ACE2 and NRP1 suggests that direct S1-ErbB binding may also play a role in viral replication. The reported ErbB1 binding by a smallpox-encoded protein (42) proposes virus-dependent ErbB engagement beyond SARS-CoV-2. The precise roles of these interactions and of ErbBs in viral entry and replication represent important topics for further studies.

Regulation of additional stages of the viral life cycle by ErbBs was suggested by the time-of-addition experiments. A recent HTS revealed that lapatinib inhibits the SARS-CoV-2 main protease (Mpro) (43), proposing one potential mechanism for its post-entry effect.

We and others provide evidence that ErbBs mediate inflammation and lung injury. In non-infectious human and animal acute lung injury/acute respiratory distress syndrome models, ErbBs are key regulators of inflammation, loss of epithelial barrier function, thrombosis, vasoconstriction, and the resulting fibrosis (20, 30–33) — processes also involved in severe COVID-19 pathogenesis (9). Indeed, transcriptomic and phosphoproteomic studies revealed that activation of ErbBs and/or their downstream pathways is among the strongest detected upon infection of human cells with SARS-CoV-1 (44), SARS-CoV-2 (34, 35), and Middle East respiratory syndrome coronavirus (MERS-CoV) (45), and in mice infected with SARS-CoV-1 (46). However, ErbB signaling has not been directly linked to coronavirus-induced inflammation and lung injury. We demonstrate SARS-CoV-2–induced activation of p38 MAPK, AKT, and ERK in human lung epithelium and ALO-derived monolayers and inhibition of phosphorylation of both ErbBs and these downstream effectors by lapatinib. Moreover, in human ALO-derived monolayers, we show that lapatinib treatment effectively suppresses SARS-CoV-2–induced secretion of proinflammatory cytokines and disruption of the lung epithelial barrier integrity. By inhibiting ErbB activation via multiple ligands implicated in lung injury — such as NRG-1, TGF-α, HB-EGF, and AREG, some of which play a role in coronaviral infections (32, 47) — lapatinib should, at least in theory, achieve a greater antiinflammatory and tissue-protective effect than approaches that target individual components of these pathways (e.g., antibodies targeting IL-1β, TGF-β, and IL-6, and p38 MAPK inhibitors) (Figure 7). It is also intriguing to speculate that by restoring ACE2 levels on the surface of SARS-CoV-2–infected cells, lapatinib may help reverse the unopposed angiotensin II effect shown to activate ErbB pathways and increase pulmonary vascular permeability in animal models of nonviral lung injury (48).

While it remains to be experimentally proven, since ErbB1 has been shown to be required for SARS-CoV-1 infection (49), and the pathways downstream of ErbBs are similarly upregulated in SARS-CoV-1– and MERS-infected cells, we predict that these findings may apply to other pandemic coronaviral infections. Lapatinib effectively suppressed the secretion of proinflammatory cytokines and loss of BBB integrity in the VEEV (TrD)–infected gNVU model, and protected mice from a lethal VEEV (TC-83) challenge, establishing that the proinflammatory and tissue-protective effects of pan-ErbB inhibition are generalizable to other viral infections and tissues. Demonstrating these protective effects in the complex, biologically relevant, human ALO-derived monolayer and gNVU models as well as in a lethal mouse model elucidates the translatability of this approach. While lapatinib has not been studied for the treatment of viral infections to date, ibrutinib, a BTK inhibitor with potent pan-ErbB activity (21) that we show suppresses SARS-CoV-2 and VEEV infections, has shown protection from progression to severe COVID-19, albeit in a small number of patients (50).

There is an urgent need to establish a therapeutic portfolio for future pandemic preparedness. The design of broad-spectrum DAAs is challenged by the genetic diversity of viral species and replication strategies; however, a host-targeted approach could overcome this challenge. Lapatinib inhibits RNA and DNA viruses from 5 viral families. Moreover, lapatinib demonstrates a higher barrier to resistance than a DAA, supporting the hypothesis that targeting host proteins that are not under the genetic control of viruses increases the barrier to resistance, in agreement with our findings with numb-associated kinase inhibitors (7). Simultaneous inhibition of several proviral kinases by a single drug (i.e., “polypharmacology”), in this case the 3 catalytically active ErbBs and possibly STK10, RAF1, and RIPK2, may further increase the effectiveness while minimizing viral resistance, as previously shown in cancer (51).

Remarkably, we provide evidence that lapatinib could achieve a synergistic effect with nirmatrelvir to inhibit SARS-CoV-2 infection. Beyond improving the antiviral effect, such synergy achieved by combination of drugs with distinct mechanisms of action could enable dose reduction and may reduce the emergence of resistant mutations, such as those selected in vitro under Paxlovid treatment and existing in clinical isolates (6), underscoring the risk of broad administration of DAAs as monotherapies.

Repurposing existing drugs requires less capital and time and diminishes the clinical risks, as such drugs have already been tested (for toxicity, pharmacokinetics, dosing, etc.) for their primary indication (9). Lapatinib is an oral drug that is approved globally in combination drug treatments for metastatic, ErbB2-positive breast cancer. Based on the available pharmacokinetics data, the plasma level achieved with the approved dose of lapatinib (1,500 mg once daily) in humans should be therapeutic, as it is 8- to 10-fold higher than the EC_50_s we measured for its antiviral effect in ALO-derived monolayers and gNVUs. Even higher lapatinib lung levels may be achieved, as suggested by the predicted lung to plasma area under the curve ratio of 8.2 to 10 (52) and measured in mouse lungs (Supplemental Figure 4F). Although toxicity is a concern when host functions are targeted, lapatinib has a favorable safety profile, particularly when used as a monotherapy and for short durations, as those required to treat acute infections. A summary of safety considerations and drug-drug interactions is provided in Supplemental Text 3.

The other hits that emerged from our HTS are discussed in Supplemental Text 4.

In summary, our study validates ErbBs as druggable targets for antiviral, antiinflammatory, and tissue-protective approaches and proposes approved drugs with anti-pan-ErbB activity as an attractive class of repurposing candidates for COVID-19 and VEEV that may provide readiness for future outbreaks of other emerging viruses.

## Methods

### Compounds, plasmids, cells, viral stocks, siRNAs, and sources of antibodies.

See Supplemental Methods for a complete list.

### HTS of compound libraries.

Compounds (final concentration 10 μM) were dispensed by Agilent Bravo pipetting system in 384-well plates (Greiner 7810192). Similarly to SARS-CoV-1 screening (8), Vero E6–eGFP cells were plated in columns 1 to 24, 24 hours before infection. Thirty microliters of assay medium was added to columns 23 and 24 (cell controls). After a 20-hour incubation, cells in columns 1–22 were infected with 30 μL SARS-CoV-2 (Belgium-GHB-03021) (MOI = 0.001), using no-contact liquid handler (EVO 100, Tecan) on the Caps-It robotics system (The Caps-It Research Infrastructure, KU Leuven). Plates were imaged after a 4-day incubation via a high-content imager (Arrayscan XTI, Thermo Fisher Scientific). eGFP signal was used as a marker for survival. Cells were excited at 485 to 20 nm, and emission was captured via a CCD camera and a BGRFRN_BGRFRN dichroic mirror and filter set (exposure time 0.023 seconds). Imaging acquisition speed was optimized using a 2 × 2 binning on 1,104 × 1,104 pixel resolution and reducing the number of autofocus focal planes. Cellomics (Thermo Fisher Scientific) software was used for image analysis. A custom-made image analysis protocol was created using the Spot Detector bioapplication (Cellomics, Thermo Fisher Scientific). SpotTotalAreaCh2 (raw value of total amount of surface covered by fluorescent cells in image) was used for further data analysis.

### Human adult lung organoids.

The adult lung organoid (ALO) model was generated from adult stem cells isolated from deep lung biopsy specimens (11). This model is complete with all 6 cell types of proximal and distal airways as validated previously (11). Lung organoid–derived monolayers were prepared (11) and plated in PneumaCult Ex-Plus Medium (StemCell Technologies).

### gNVU model.

gNVUs were prepared as described in refs. 15, 16.

### Infection assays and pharmacological inhibition.

Unless stated otherwise, inhibitors or DMSO were added to the cells 1 hour before viral inoculation and were left for the duration of the experiment. Calu-3 cells, Vero cells, or ALOs were infected with SARS-CoV-2 in 2–3 replicates (MOI = 0.05 or 1) in DMEM containing 2% FCS at 37°C under biosafety level 3 (BSL3) conditions. After 1- to 3-hour incubation, the inoculum was removed, and cells were washed and supplemented with new medium. Culture supernatants were harvested for measurement of viral titer by standard plaque assays, and cells were lysed in TrizolLS (Invitrogen) for RT-qPCR analysis. Huh7 cells were infected with DENV2 in replicates (*n* = 2–4) (MOI = 0.05). At 48 hpi, infection was measured via luciferase or plaque assays. Huh7 cells were infected with EBOV (MOI = 1) or MARV (MOI = 2) under BSL4 conditions. At 48 hpi, cells were formalin-fixed for 24 hours before removal from BSL4. Infected cells were detected using an EBOV or MARV glycoprotein–specific mAb (KZ52 and 7E6, respectively) and quantitated by automated fluorescence microscopy using an Operetta High Content Imaging System (PerkinElmer). U-87 MG cells were infected with VEEV-TC-83-Nluc in 3–4 replicates (MOI = 0.01) or with WT VEEV (TrD) in triplicate. At 24 hpi, infection was measured via nanoluciferase or plaque assays. A549 cells were infected with MPOXV in 4 replicates (MOI = 0.005). At 24 hpi, cells were fixed, and infection was measured using Focus forming reduction assay (FFRA) by immunostaining using anti–VACV A33R antibody. Virus plaques were counted using an ImmunoSpot plate reader, as described previously (53).

gNVUs were perfused with lapatinib- or DMSO-containing culture medium for 1 hour followed by introduction of medium containing VEEV (TrD) (MOI = 0.1) into the vascular inlet and 1-hour incubation at 37°C. Lapatinib-containing medium was reintroduced into the gNVU daily, and the units were maintained at 37°C, 5% CO_2_ conditions for up to 120 hours (study duration).

### Viability assays.

Viability was assessed using alamarBlue reagent (Invitrogen) according to the manufacturer’s protocol. Fluorescence was detected at 560 nm on an InfiniteM1000 plate reader (Tecan). MTT assay (Promega) was used to determine A549 cell viability according to the manufacturer’s protocol at 570 nm using a microplate reader (BioTek Synergy).

### Combination drug treatment.

Calu-3 cells were treated with lapatinib-DAA combinations and infected with rSARS-CoV-2/Nluc (USA-WA1/2020 strain) (MOI = 0.05). At 24 hpi, the antiviral effect was measured via Nluc assay, and cellular viability was measured via alamarBlue assay. The MacSynergy II program was used for data analysis, as described previously (7, 12). Matrix data sets in 3 replicates were assessed at the 95% confidence interval for each experiment.

### siRNA transfection.

siRNAs (10 pmol/well of a 96-well plate) were transfected into cells using Lipofectamine RNAiMAX transfection reagent (Invitrogen) 48 hours before viral infection.

### Time-of-addition assay.

Calu-3 cells were infected with SARS-CoV-2 (MOI = 1). At 2 hpi, virus was removed, and cells were washed twice with PBS. At distinct time points, 10 μM lapatinib or 0.1% DMSO was added. Cell culture supernatants were collected at 10 hpi, and infectious viral titers were measured by plaque assay.

### Temperature shift assay.

Vero cells were either concurrently inoculated with rVSV-SARS-CoV-2-S and treated with lapatinib (10 μM) or DMSO at 4°C for 2 hours and washed, or infected with the virus at 4°C for 2 hours, washed, and then treated with lapatinib (10 μM) or DMSO at 37°C for 2 hours. Entry was measured at 24 hpi.

### Entry assays.

At 48 hours after siRNA transfection, Calu-3 cells were infected with WT SARS-CoV-2 (MOI = 1). At 1 hpi, cells were washed 3 times with PBS and fresh medium added. At 2 hpi, cells were lysed in TRIzolLS (Invitrogen), and intracellular viral RNA levels were measured by RT-qPCR.

### RT-qPCR.

RNA was extracted from cell lysates using Direct-zol RNA Miniprep Plus Kit (Zymo Research) and reverse-transcribed using a High-Capacity cDNA RT kit (Applied Biosystems). Primers and PowerUp SYBR Green Master Mix (Applied Biosystems) were added to the samples, and PCR reactions were performed with QuantStudio3 (Applied Biosystems) in triplicate. Target genes were normalized to GAPDH. Sequences of primers used for RT-qPCR are listed in Supplemental Methods.

### Gain-of-function assays.

Plasmids encoding ErbB4 or control were transfected into cells using Lipofectamine 3000 reagent (Invitrogen) 24 hours before drug treatment and viral infection. Viral infection and cell viability were measured 24 hours later via luciferase and alamarBlue assays, respectively.

### Resistance studies.

VEEV (TC-83) was used to inoculate U-87 MG cells (MOI = 0.1) and passaged daily under increasing drug selection (2.5–5 μM, passages 1–3; 5–10 μM, passages 4–7; 10–15 μM, passages 8–10). After 10 passages, viral titers were measured in culture supernatants by plaque assays. ML336-resistant mutation emerging in nsP2 at passage 10 was confirmed by purification and reverse transcription of viral RNA from cell supernatants using RNeasy Mini Kit (Qiagen) and SuperScript IV First-Strand Synthesis kit (Invitrogen). The nsP2 region was amplified with Platinum Green Hot Start PCR Master Mix (2×) (Invitrogen) using primers aggaaaatgttagaggagcacaag (forward) and gtcaatatacagggtctctacggggtgt (reverse) and sequenced (Sequetech Corp.).

### Signaling pathway analysis.

After 2-hour starvation, Calu-3 cells or ALO-derived monolayers were treated with lapatinib or DMSO and, within an hour, infected with SARS-CoV-2 (MOI = 1). Cell lysates were obtained at 1.5 and/or 24 hpi followed by Western blot analysis with antibodies targeting the phosphorylated and total protein forms (see Supplemental Methods).

Phosphorylated to total protein ratios were quantified with ImageJ software (NIH).

### Cytokine measurements.

Cytokine concentrations in ALO supernatants were quantified using a LEGENDplex Human Inflammation Panel 1 (BioLegend) kit and read on a Quanteon flow cytometer (Agilent). Data were analyzed using LEGENDplex v8.0 software. Cytokine levels in gNVU perfused media were quantified using the MSD V-PLEX Proinflammatory Panel Human Kit (Meso Scale Discovery) and MESO QuickPlex SQ 120 (Meso Scale Discovery) reader.

### BBB permeability assay.

A 3 kDa FITC-dextran assay (Thermo Fisher Scientific) was used, and the effective permeability of the brain microvascular endothelial cell monolayer was calculated, as described previously (16).

### Animal studies.

C3H/HeN female mice (Charles River Laboratories) (6–8 weeks of age; *n* = 2–5 per group) were inoculated intranasally with 5 × 10^6^ PFU of VEEV (TC-83). Animals were pretreated for 12 hours and post-treated once or twice a day with lapatinib (200 mg/kg) in 0.5% hydroxypropyl methylcellulose with 0.1% Tween 80 as vehicle through oral gavage. Survival was monitored for up to 2 weeks. Control mice were TC-83 infected and treated with vehicle only. Viral titers were measured in both serum and brain by plaque assays. Mice were euthanized with CO_2_ upon meeting morbidity criteria or at the end of the experiment, and 500–700 μL of blood was collected with a 23-gauge needle by cardiac puncture. The blood was spun for 5 minutes at 14,000*g*, serum was collected, and plaque assays were performed. Mouse brains were collected from euthanized animals, homogenized in DMEM-supplemented media using IKA ULTRA-TURRAX Tube drive and DT-20 tube system, and used for plaque assays. Multi-dose pharmacokinetics study in C57BL/6 mice was conducted by Sai Life Sciences.

### Immunofluorescence and confocal microscopy.

ALO-derived monolayers were washed with PBS, fixed, blocked, and incubated with mouse mAb SARS-CoV-2 nucleocapsid antibody and rabbit claudin-7 polyclonal antibody overnight at 4°C, followed by incubation with secondary antibodies and counterstaining with DAPI (Thermo Fisher Scientific) and phalloidin (Thermo Fisher Scientific). Images were taken on an SP8 microscope (Leica).

For imaging of viral particles, Vero E6–TMPRSS2 cells were pretreated with lapatinib (10 μM) or DMSO for 1 hour at 37°C and infected with SARS-CoV-2 (MOI = 1) at 4°C. After 1-hour incubation, infected cells treated with lapatinib or DMSO were subjected to temperature shift to 37°C to initiate virus infection. At 1 hpi, cells were washed and fixed as described above. Cells were incubated with SARS-CoV-2 nucleocapsid and Rab7 antibody followed by a secondary antibody. Images were taken using an LSM 880 microscope (Zeiss) with ×63 objective. Colocalization was quantified using ImageJ (JACoP) colocalization software and Manders’ colocalization coefficients.

### Coimmunoprecipitations.

A549-NRP1^KO^ cells were cotransfected with plasmids expressing S1-FLAG and ErbB1, ErbB2, or ErbB4 using Lipofectamine 3000 reagent. At 24 hours after transfection, the cells were lysed with M-PER protein extraction reagent (Thermo Fisher Scientific). Clarified supernatants were precleared with Dynabeads protein G (Invitrogen) for 1 hour at 4°C and incubated with either anti-ErbB1, -ErbB2, or -ErbB4 or IgG antibody overnight at 4°C. The antibodies and bound proteins were captured by protein G Dynabeads for 2 hours at 4°C. Beads were washed and resuspended in SDS sample buffer.

### Flow cytometry.

Calu-3 cells were pretreated with lapatinib (10 μM) or DMSO for 1 hour at 37°C and infected with SARS-CoV-2 (MOI = 1) at 4°C. After 1-hour incubation, the temperature was shifted to 37°C to initiate infection. At 0.5 and 1 hour after temperature shift, cells were washed with PBS and incubated for 15 minutes at room temperature with Zombie Aqua live/dead fixable dye (BioLegend) and FcR Blocking Reagent (Miltenyi Biotec). Cells were stained for 20 minutes at 4°C with anti-NRP1–BV421, anti-ACE2–APC, and anti-ErbB2–Alexa Fluor 488 antibodies, or with their corresponding isotype controls. Unbound antibody was washed, and cells were fixed with 4% PFA for 1 hour at room temperature. Cell acquisition was performed on an Aurora Cytek spectral flow cytometer, and data were analyzed using FlowJo software (Tree Star).

### Statistics.

Data were analyzed with GraphPad Prism software. EC_50_ and CC_50_ values were measured by fitting of data to a 3-parameter logistic curve. *P* values were calculated by 1-way ANOVA with either Dunnett’s or Tukey’s multiple-comparison tests or by 2-tailed Student’s *t* test. A *P* value of 0.05 or less was considered significant.

### Study approval.

SARS-CoV-2, VEEV, and filovirus work was conducted in BSL3 and BSL4 facilities at Stanford University, KU Leuven Rega Institute, George Mason University, and the US Army Medical Research Institute of Infectious Diseases according to CDC and institutional guidelines. Human lung organoid propagation was approved under protocol IRB 190105 at UCSD. Animal experiments were approved by the Institute of Laboratory Animal Resources, National Research Council, NIH Publication 86-23.

### Data availability.

Values for all data points in graphs are reported in the Supporting Data Values file. See complete unedited blots in the supplemental material.

## Author contributions

SS, MK, LG, PTH, WC, VD, CWL, SK, NB, PL, FA, NAB, DHNT, CAC, KEH, MS, AMK, and KC designed and performed the experiments and conducted data analysis. JAB, CT, CY, PG, SD, DESC, JJ, and JPW provided reagents and guidance. SE, SDJ, DJ, DESC, JN, AN, BAP, and LMS provided scientific oversight and guidance. SS, SE, SDJ, MK, WC, JMD, and DJ wrote the first version of the manuscript. SE, DJ, JN, AN, SDJ, and JMD provided funding for the studies.

## Supplementary Material

Supplemental data

Supporting data values

## Figures and Tables

**Table 2:**
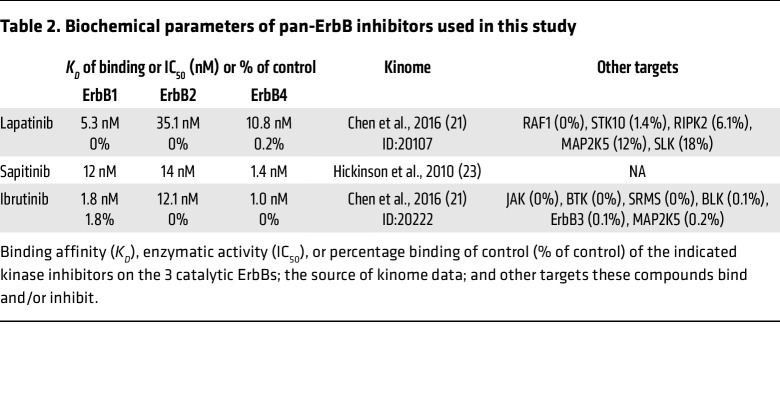
Biochemical parameters of pan-ErbB inhibitors used in this study

**Table 1:**
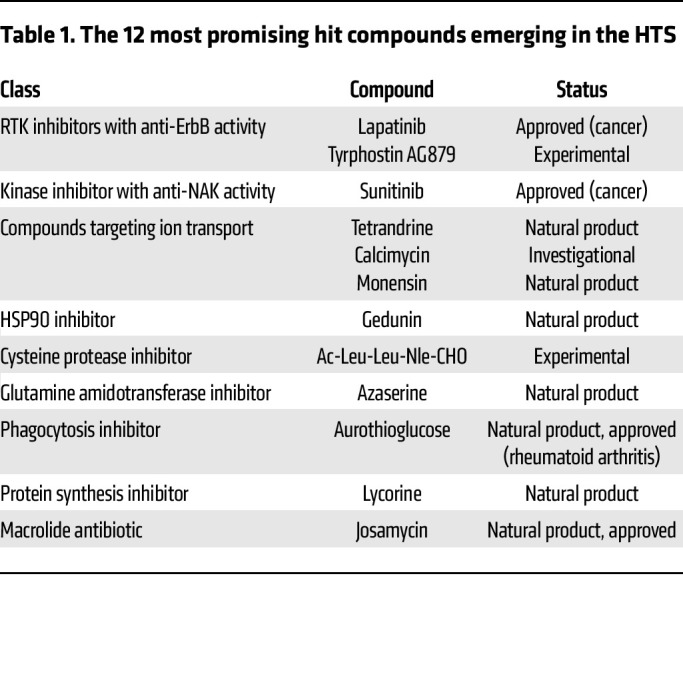
The 12 most promising hit compounds emerging in the HTS

## Version 1 08/15/2023

In-Press Preview

## Version 2 10/02/2023

Electronic publication
