# Supplementary material for: Anticancer pan-ErbB inhibitors reduce inflammation and tissue injury and exert broad-spectrum antiviral effects
Source: J Clin Invest. 2023 Oct 2;133(19):e169510. doi: 10.1172/JCI169510 (PMC10541190; doi:10.1172/JCI169510)

Supplemental data

This document includes:

1. Supplemental figures and figure legends

2. Supplemental texts

3. Supplemental methods

4. Supplemental data references

5. Uncut gels

Supplemental figures

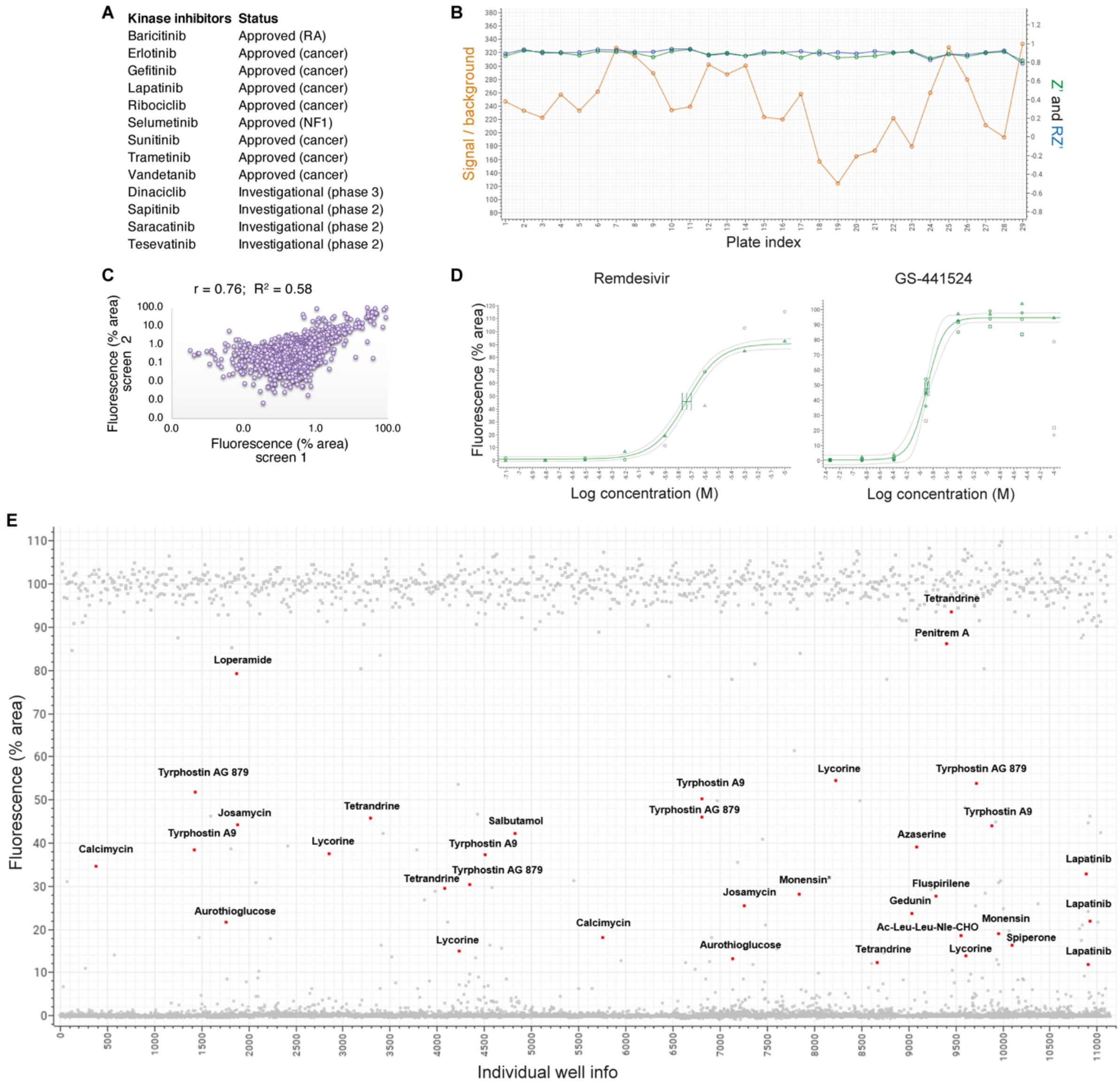

Supplemental Figure 1: Characteristics of the HTS. Related to figure 1.
A, The kinase inhibitors included in the self-assembled set. **B,** Quality control of each individual plate of the 29 screened by determination of the signal-to-background (S/B), and the Z' and RZ' values. All three parameters were measured for each 384-well screening plate using the virus control (infected, DMSO treated) and cell control (uninfected, untreated) wells. S/B values ranged from 124 – 333. Z' and RZ' values were > 0.78. Generally, S/B values >10 and (R)Z' values >0,5 are accepted as qualitative assays. All parameters were calculated using Genedata Screener. **C,** Scatter plot of the two replicate screens with a Pearson's correlation coefficient (*r*) of 0.76 and R^2 0.58. **D**, Dose-dependent rescue of Vero-eGFP cells from SARS-CoV-2-induced lethality by remdesivir and its major active metabolite, GS-441524, used as positive controls, 4 days post-infection with SARS-CoV-2 (Belgium-GHB-03021, MOI=0.001). **E**, Percentage of fluorescence area values from all wells including the virus controls (infected, DMSO treated) and the cell controls (uninfected, untreated) from the 29 384-well plates. The red dots depict hits emerging in the screening. Grey dots represent reference compounds such as nelfinavir, GS-441524 and compounds not prioritized for further analysis.

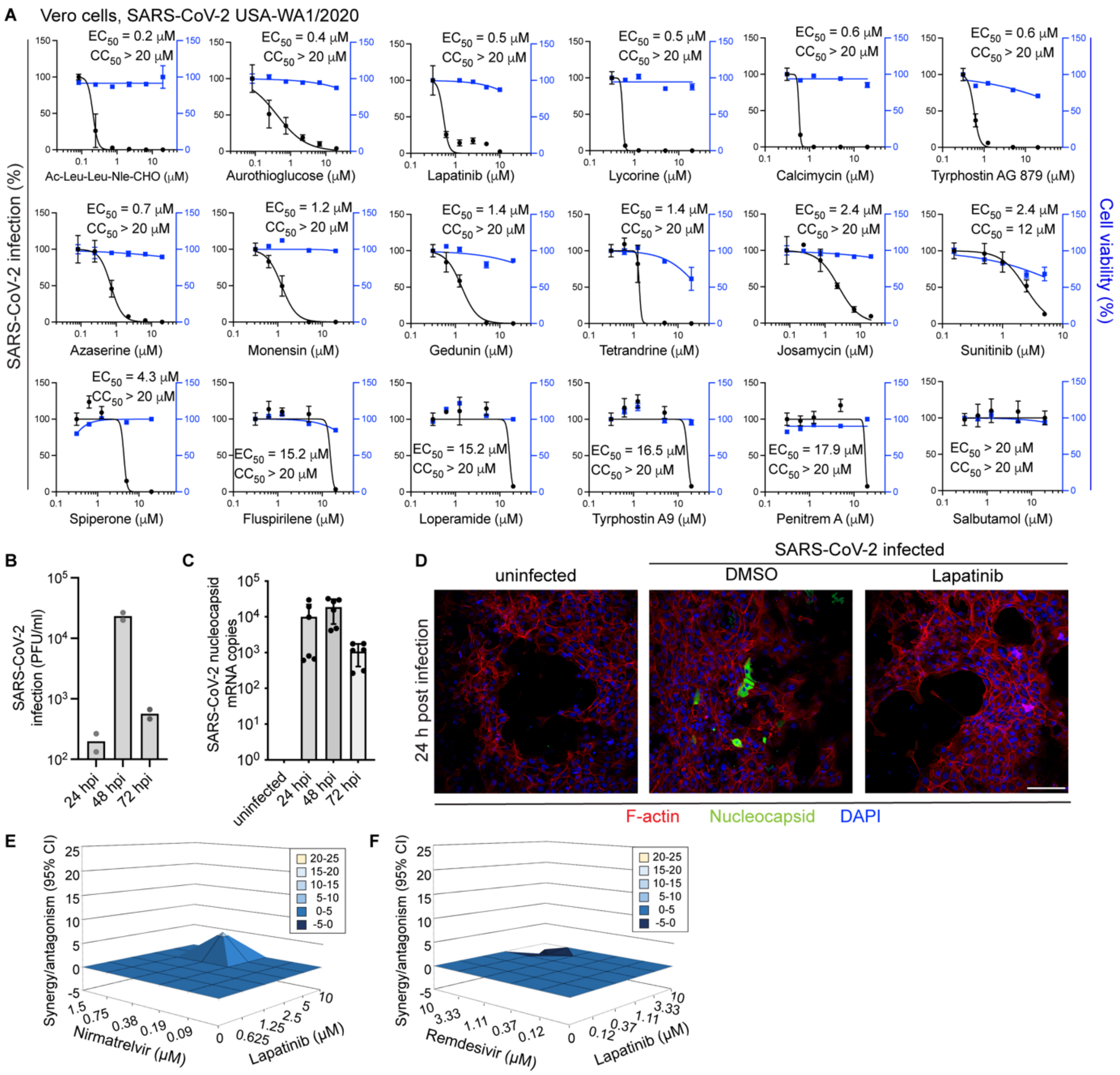

Supplemental Figure 2. Validation of hits emerging from the HTS and characterization of human ALO-derived monolayers for studying the antiviral effect of emerging hits. Related to figures 1 and 2.

A, Dose response curves to the indicated hits emerging from the HTS of SARS-CoV-2 infection (black, USA-WA1/2020 strain, MOI=0.05) and cell viability (blue) in Vero cells measured via plaque and alamarBlue assays at 24 hpi, respectively. **B, C,** Viral titer by plaque assays in culture supernatants (**B**) and viral nucleocapsid (N) copy number analyzed by RT-qPCR in lysates (**C**) from human ALO-derived monolayers at 24, 48 and 72 hpi. **D**, Confocal IF microscopy images of F-actin (red), SARS-CoV-2 nucleocapsid (green) and DAPI (blue) in naïve and SARS-CoV-2-infected ALO-derived monolayers pre-treated with DMSO or 10 µM lapatinib at 24 hpi. 20x magnification of the images in figure 2J are shown. Scale bar is 100 µm. **E, F**, Synergy/antagonism of lapatinib and nirmatrelvir (**E**) or remdesivir (**F**) combination treatment on cellular viability measured in Calu-3 cells infected with rSARS-CoV-2/Nluc (USA-WA1/2020 strain) at 24 hpi via alamarBlue assays. Data represent differential surface analysis at the 95% confidence interval (CI), analyzed via the MacSynergy II program. Synergy and antagonism are indicated by the peaks above and below the theoretical additive plane, respectively. The level of synergy or antagonism is depicted by the color code. Data are representative (A, B, D-F) or combination (A, C) of two

independent experiments with 2-3 replicates each. Means±SD are shown (A, C). Data in A is relative to DMSO control.

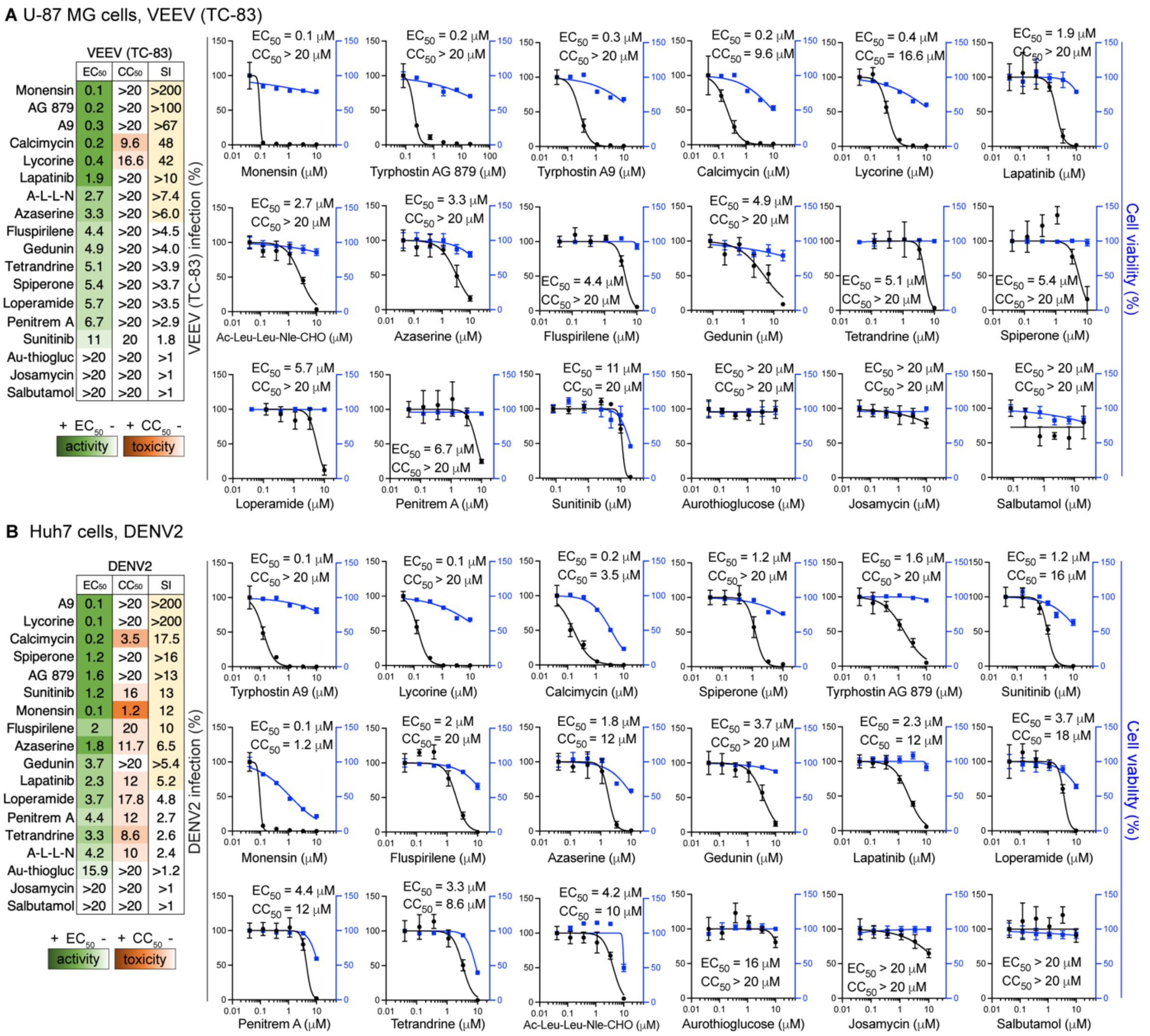

Supplemental Figure 3. Broad-spectrum potential of hits. Related to figures 1 and 2. **A, B,** The 18 compounds emerging from the HTS were tested for their effect on VEEV (TC-83) (**A**) and DENV2 (**B**) infections in U-87 MG and Huh7 cells, respectively, via luciferase assays, and for their effect on cell viability via alamarBlue assays. Left panels: Heat maps of the EC_{50} and CC_{50} values of the indicated compounds color-coded based on the antiviral activity (green) and toxicity (orange). Selectivity indices (SI) greater than 5 are depicted in yellow. Right panels: Dose response curves to the indicated compounds of VEEV (TC-83) (MOI=0.1) or DENV2 (MOI=0.05) infections (black) in U-87 MG and Huh7 cells, respectively, measured via luciferase assays and cell viability (blue) measured by alamarBlue assays at 24 hours post-infection. Data are representative of two independent experiments with 4 replicates each. Means±SD are shown. Data are relative to DMSO control.

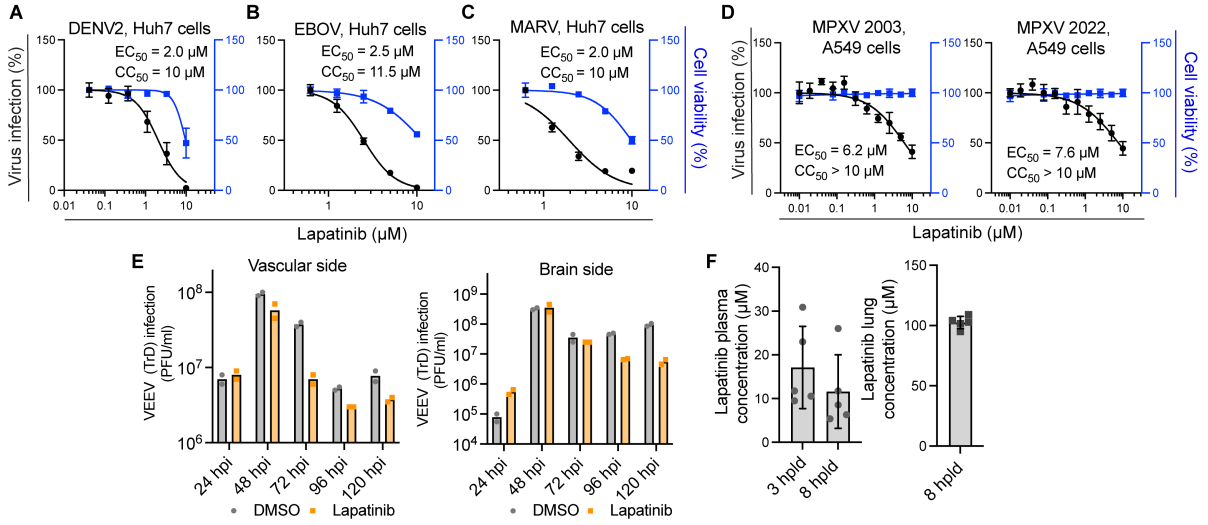

Supplemental Figure 4: Lapatinib is a potent broad-spectrum antiviral. Related to figure 3.

A, Dose response of DENV2 infection (black) and cellular viability (blue) to lapatinib measured in Huh7 cells via plaque and alamarBlue assays at 24 hpi (MOI=0.1), respectively. **B, C,** Dose response of EBOV (Kikwit isolate, MOI=1) (**B**) and MARV (Ci67 strain, MOI=2) (**C**) infections (black) and cellular viability (blue) to lapatinib measured in Huh7 cells 48 hpi via microneutralization assay and CellTiter-Glo luminescent cell viability assay, respectively. **D**, Dose response of MPOXV 2003 and 2022 infection (black) and cellular viability (blue) to lapatinib measured in A549 cells via Focus forming reduction assay (FFRA) and MTT assays at 24 hpi (MOI=0.005), respectively. **E,** Viral load in longitudinal samples collected from the vascular and brain sides of the gNVU following infection with VEEV (TrD) and treatment with lapatinib or DMSO. **F,** lapatinib's plasma and lung concentrations after 8 days of twice daily treatment with 200 mg/kg in C57BL/6 mice measured 3 and 8 hours post last dose (hpld). Data are combination (A) or representative (B, C, D, E) of two independent experiments with 2-5 replicates each. Means±SD are shown (A-D, F). Data in A-D are relative to DMSO control.

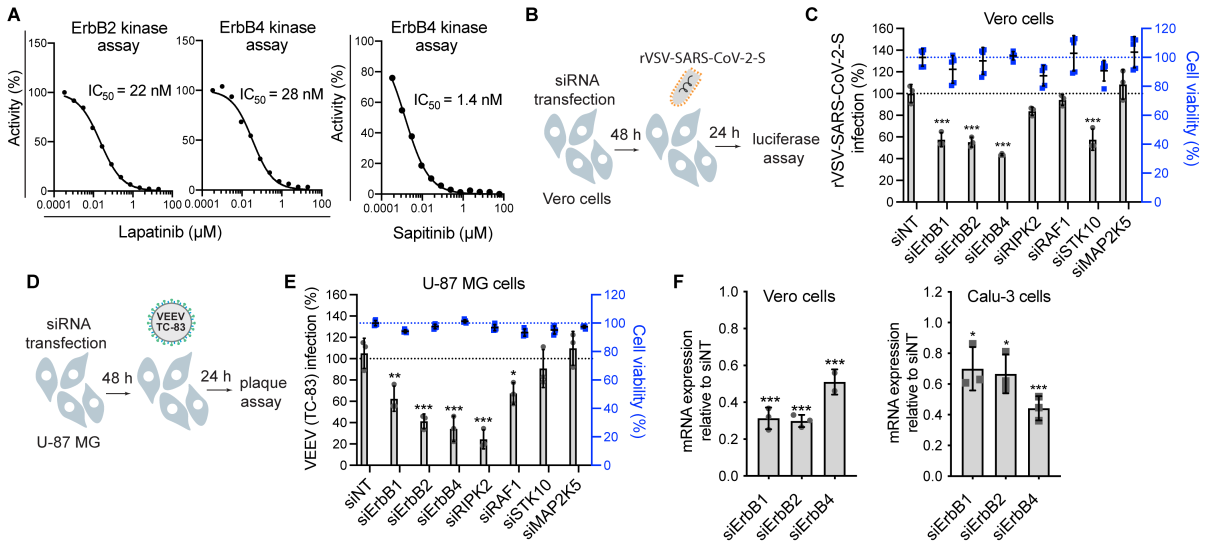

Supplemental Figure 5: Validation of ErbBs as an antiviral target. Related to figure 4. **A**, Dose response to lapatinib and sapitinib of ErbB2 and/or ErbB4 kinase activity in vitro (Nanosyn). **B**, Schematic of the experiment shown in panel C. **C,** Percentage of infection by luciferase assays (grey) and cell viability by alamarBlue assays (blue) measured at 24 hpi of Vero cells transfected with the indicated siRNA pools with rVSV-SARS-CoV-2-S pseudovirus. **D**, Schematic of the experiment shown in panel E. **E**, Percentage of infection by plaque assays (grey) and cell viability by alamarBlue assays (blue) measured at 24 hpi of U-87 MG cells transfected with the indicated siRNA pools with VEEV (TC-83). **F**, Confirmation of siRNA-mediated gene expression knockdown by RT-qPCR in Vero and Calu-3 cells. Shown is gene expression normalized to GAPDH and expressed relative to the respective gene level in the siNT control at 48 hours post-transfection. Data are representative (C, E, F) of two independent experiments with 3-5 replicates each. Means±SD are shown (C, E, F). Data are relative to DMSO (A) or siNT (C, E, F) controls. **P* < 0.05, ****P* < 0.001 relative to siNT by one-way ANOVA followed by Dunnett's multiple comparisons test.

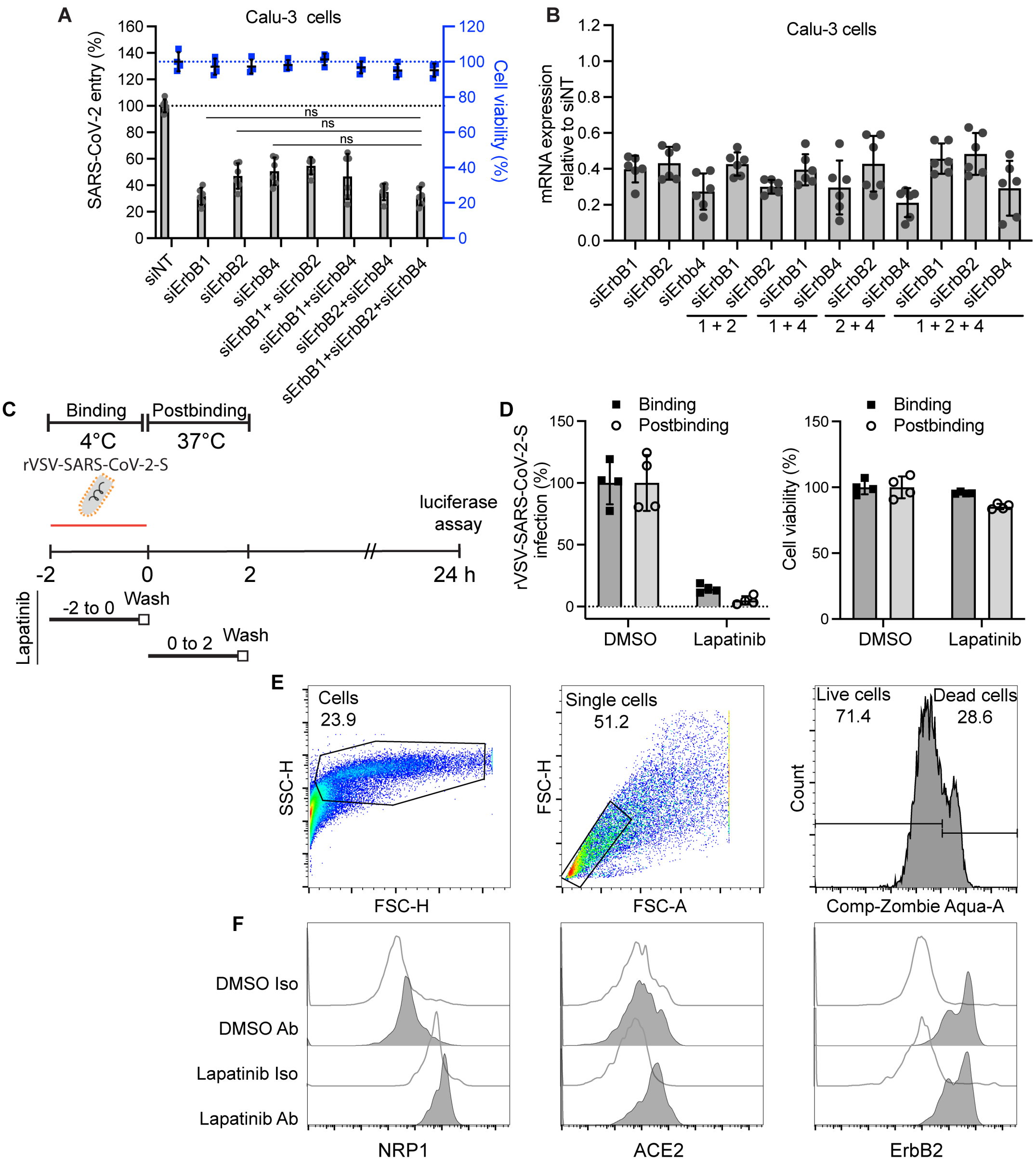

Supplemental Figure 6: Lapatinib treatment suppresses viral entry at a postbinding stage. Related to figure 5.

A, WT SARS-CoV-2 entry at 2 hpi of Calu-3 cells (MOI=1) depleted of the indicated ErbBs individually or in double and triple combinations measured by RT-qPCR. **B**, Confirmation of siRNA-mediated gene expression knockdown by RT-qPCR in Calu-3 cells transfected with the indicated siRNAs. Shown is gene expression normalized to GAPDH and expressed relative to the respective gene level in the siNT control at 48 hours post-transfection. 1+2, 1+4, 2+4 refer to ErbB combinations in double knockdown. 1+2+4 depicts simultaneous knockdown of ErbB1, ErbB2 and Erbb4. **C**, Schematic of the temperature-shift experiments shown in panel D**. D,** Vero cells were infected with VSV-SARS-CoV-2-S for 2 hours at 4°C in the presence or absence of 10 µM lapatinib or DMSO before the temperature was shifted to 37°C to initiate infection. 24 hpi virus infection was measured via luciferase assay and cell viability by alamarBlue assay. **E**, Representative dot plots showing gating strategy. Cell debris were excluded by size, and dead cells were excluded using Zombie Aqua live/dead staining. **F**, Representative histograms depicting surface expression of NRP1, ACE2 and ErbB2 in SARS-CoV-2-infected and DMSO- or lapatinib-treated cells. Antibody isotype

control histograms are also shown. Data are combination (A, B) or representative (D) of two independent experiments with 2-4 replicates each. Means±SD are shown (A, B, D). Data are relative to siNT (A, B) or DMSO (D) controls.

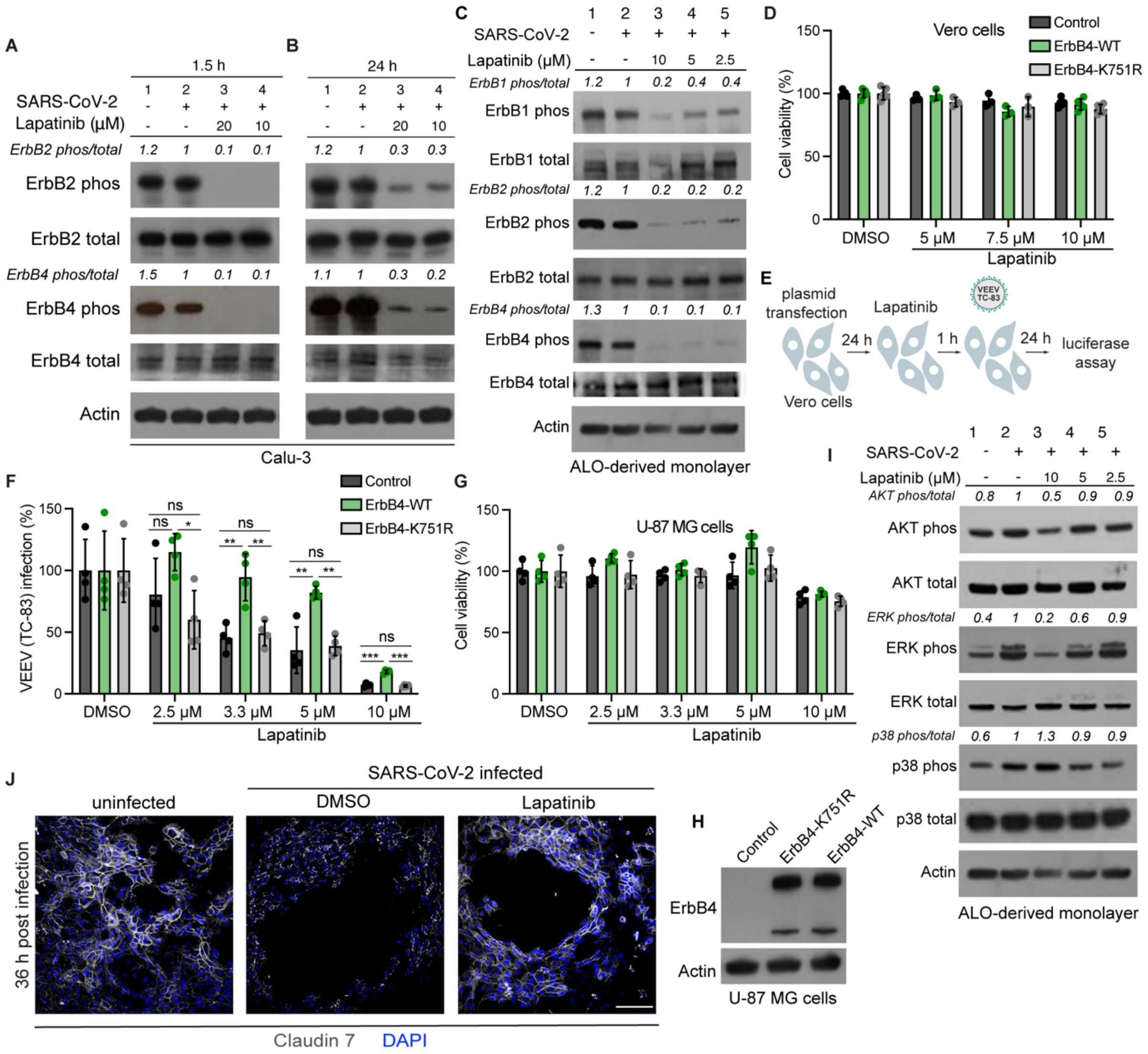

Supplemental Figure 7: Lapatinib treatment modulates ErbBs and suppresses activation of downstream tissue injury signals. Related to figure 6.
A, B, ErbB2 and ErbB4 phosphorylation in Calu-3 cells that are uninfected (lane 1), infected and treated with DMSO (lane 2) or infected and treated with lapatinib (lanes 3 and 4) measured by Western blotting 1.5 (**A**) and 24 (**B**) hpi with SARS-CoV-2 (USA-WA1/2020 strain, MOI=1). **C,** Dose-dependent effect of lapatinib treatment on ErbB1, 2 and 4 phosphorylation in ALO-derived monolayers that are uninfected (lane 1), SARS-CoV-2-infected and treated with DMSO (lane 2) or infected and treated with lapatinib (lanes 3-5) measured by Western blotting 48 hpi. Shown are representative membranes blotted for phospho- and total ErbB2, ErbB4, and actin and quantitative phospho- to total ErbB ratio data relative to infected cells treated with DMSO (lane 2). **D,** Vero cell viability measured by alamarBlue assays 48 hours post-transfection of the indicated plasmids. **E,** Schematic of the experiments shown in F-H. **F,** Rescue of VEEV (TC-83) infection in the presence of lapatinib upon ectopic expression of the indicated plasmids measured by luciferase assays 24 hpi in U-87 MG cells. **G,** U-87 MG cell viability measured by alamarBlue assays 48 hours post-transfection of the indicated plasmids. **H**, Level of ErbB4 and actin expression measured via Western blot following transfection of U-87 MG cells with control or ErbB4-expressing plasmids. **I**, Dose-dependent effect of lapatinib treatment on AKT, ERK and p38 MAPK

phosphorylation in ALO-derived monolayers that are uninfected (lane 1), SARS-CoV-2-infected and treated with DMSO (lane 2) or infected and treated with lapatinib (lanes 3-5) measured by Western blotting 48 hpi. Membranes shown in C and I were cut from the same membrane prior to blotting with the indicated antibodies based on the molecular weight marker and size of proteins of interest. Therefore, the actin membrane is the same for both insets. **J,** Confocal IF microscopy images of Claudin 7 (grey) and DAPI (blue) in naïve or SARS-CoV-2- infected ALO-derived monolayers treated at 4 hpi either with DMSO or 10 μM lapatinib and imaged at 36 hpi. 20x magnification of the images in figure 6I are shown. Scale bar is 100 μm. Data are combination (D) or representative (F, G) of two independent experiments with 2-4 replicates each. Means±SD are shown (D, F, G). **P* < 0.05, **P < 0.005, ****P* < 0.001 relative to DMSO control by one-way ANOVA followed by Tukey's multiple comparisons test at each lapatinib concentration.

Supplemental text 1. The HTS of compound libraries for SARS-CoV-2 inhibitors is robust and specific.

The libraries were screened in two independent experiments. Data were normalized to the median of each plate. The average percent fluorescent area for control wells included in each plate was 102.9±5% for uninfected cells (cell control), 0.1±0.2% for infected untreated cells (virus control), and 0.0±0.164% for infected cells treated with DMSO (Figure 1C). The *Z*-score was calculated on the basis of the log2(fold change) (log2FC) with the average and standard deviation of each plate. The Z' and RZ' values of each of the 29 screen plates, calculated based on the virus control and cell control wells, were greater than 0.78 and the signal-to-background (S/B) value, representing the ratio of the median value of the raw data between the virus control and the cell control, was greater than 120 (Supplemental Figure 1B). The two replicate screens demonstrated good correlation (r= 0.76) (Supplemental Figure 1C). Remdesivir and its major metabolite, GS-441524, used as positive controls, demonstrated dose-dependent anti-SARS-CoV-2 activity in this assay (Supplemental Figure 1D). Overall, these data indicate that this antiviral assay is robust for HTS and is specific. 40 compounds from the screen were selected according to the cutoff of fluorescence % area greater than 15 in at least one of the two screens, which is 15 times greater than the values obtained with untreated or DMSO treated cells.

Supplemental text 2. Broad-spectrum antiviral activity of hits.

The effect of the 18 emerging hit compounds on replication of two unrelated RNA viruses, TC-83, the vaccine strain of the alphavirus VEEV, and the flavivirus, dengue (DENV2) was measured in human astrocytes (U-87 MG) and human hepatoma (Huh7) cells, respectively, both via luciferase assays. Lycorine, calcimycin, monensin, azaserine, gedunin, and the kinase inhibitors lapatinib and AG 879 dose-dependently inhibited replication of TC-83 and DENV2 in addition to SARS-CoV-2 (Supplemental Figure 3A, B). Several compounds, such as tyrphostin A9, an investigational inhibitor of PDGFR (platelet-derived growth factor receptor), and fluspirilene, a neuroleptic agent, demonstrated more potent anti-TC-83 and DENV2 activity than anti-SARS-CoV-2 activity, and others showed variable activity against one or two of these viruses. Salbutamol demonstrated minimal to no activity against all three viruses (Supplemental Figure 3A, B).

Supplemental text 3. Safety considerations and drug-drug interactions of lapatinib. Related to discussion.

Although toxicity is a concern when targeting host functions, lapatinib has a favorable safety profile, particularly when used as a monotherapy and for short durations, as those required to treat acute infections.

Notably, lapatinib's safety profile in the package insert was based on data from over 12,000 patients with advanced cancer who received lapatinib in combination with capecitabine or trastuzumab plus an aromatase inhibitor and for long durations (1-3). As monotherapy, lapatinib was tested in several open-label studies with a median duration of 7-28 weeks in patients with advanced cancer (4-11). The most common adverse events attributed to lapatinib were diarrhea, rash, nausea, pruritus, and fatigue, with diarrhea being the most common adverse event resulting in drug discontinuation. The most common laboratory abnormalities with combination therapy were increased liver function tests, which were infrequently severe (1-3, 12). More severe adverse events including transient, reversible decreases in left ventricular ejection fraction, prolongation of QT interval, and hepatotoxicity, were also documented, yet infrequently, and with the exception of cardiac toxicity, primarily in patients receiving lapatinib in combination treatment (1, 10, 13, 14).

Notably, unlike erlotinib and gefitinib, lapatinib monotherapy has not been associated with pneumonitis, interstitial lung disease or lung fibrosis (4-11). The estimated incidence of 0.2% for these adverse effects is based on patients receiving lapatinib in combination with other drugs (15-20) known to cause pneumonitis and/or lung fibrosis (21-23), and sometimes also with radiation, for a median duration of 24-45 weeks. We predict that lapatinib's distinct off-target profile accounts for this difference in the occurrence of these adverse events. Indeed, cyclin G-associated kinase (GAK), an off-target of erlotinib (K_D=3.1 nM, IC_{50}=0.88 μM) and gefitinib (K_D=6.5 nM, IC_{50}=0.41 μM), but not of lapatinib (K_D=980 nM, IC_{50}>5 μM)(24), has been implicated in pulmonary alveolar function and stem cell regeneration, and its inhibition is thought to be the mechanism underlying gefitinib- and erlotinib- induced lung toxicity(25, 26).

An important consideration with lapatinib is, however, its potential for drug-drug interactions. Since metabolized by CYP3A4, concurrent use of suppressors of CYP3A4 should be avoided to reduce risk of QT prolongation. Concurrent treatment with CYP3A4 inducers should also be avoided, as this can reduce lapatinib's levels to sub-therapeutic. Of particular relevance is the CYP3A4 inducer dexamethasone used as standard of care for moderate COVID-19 patients. Since other steroids do not induce CYP3A4, lapatinib could be studied in combination with hydrocortisone or prednisone, which have been shown to comparably protect COVID-19 patients (27-29).

Supplemental text 4. Broad-spectrum potential of other hits emerging in the screen. Related to discussion.

Another approved anticancer drug that emerged in the HTS was sunitinib, a multi-kinase inhibitor that we have shown to protect mice from DENV and EBOV challenges when given in combination with erlotinib by inhibiting NAK-mediated intracellular viral trafficking(30-32).

Sunitinib was recently shown by others to suppress pan-corona pseudotyped viral infections (33) and by us to suppress WT SARS-CoV-2 infection (34). AG 879, another kinase inhibitor demonstrating anti-SARS-CoV-2 activity, was reported to suppress replication of multiple viruses including a mouse hepatitis virus (Coronaviridae) in cultured cells and to protect mice from influenza A virus (IAV) challenge (35-37). Nevertheless, since we could not confirm its anti-ErbB activity, the precise target(s) mediating the antiviral effect remain to be elucidated.

Ion transport across cell membranes is another function that emerged in our HTS as a candidate target for anti-SARS-CoV-2 approaches. Among the hits was tetrandrine, a calcium channel blocker with anti-inflammatory and anti-fibrogenic properties used as a medicinal herb for the treatment of lung silicosis, liver cirrhosis, and rheumatoid arthritis (38). Tetrandrine was previously shown to inhibit EBOV entry in cultured cells and protect EBOV-infected mice by inhibiting endosomal calcium channels (39). Monensin, an antiprotozoal agent, and calcimycin, shown to inhibit VSV and IAV infections (40, 41), are both ionophores that facilitate the transport of sodium/potassium and calcium across the membrane, respectively. Spiperone, an activator of chloride channels licensed in Japan for the treatment of schizophrenia, was another hit.

The emergence of gedunin, a natural product that inhibits HSP90 and has anti-inflammatory properties, suggests a potential role for HSP90 in SARS-CoV-2 infection, as in other viral infections (42, 43). Lycorine, a protein synthesis inhibitor (44) was also shown to suppress replication of multiple viruses including SARS-CoV in cultured cells (45-48) and mortality of mice infected with human enterovirus 71 (49). The underlying mechanism of action in influenza was thought to be inhibition of export of viral ribonucleoprotein complexes from the nucleus (45), yet lycorine also exhibits anti-inflammatory effects (50). Azaserine is a natural serine derivative that irreversibly inhibits γ-glutamyltransferase in the metabolic hexosamine pathway. Independently of this target, it was shown to protect from endothelial cell inflammation and injury (51).

Aurothioglucose has been used for the treatment of rheumatoid arthritis and is thought to inhibit the activity of adenylyl cyclase in inflammatory pathways (52). Ac-Leu-Leu-Nle-CHO is used as a research tool to inhibit calpain 1 and 2 (CAPN1 and 2) (53), cysteine proteases required for SARS-CoV (54), echovirus 1 (55) and herpes simplex virus (56) infections. Targeting calpain proteases was shown to inhibit SARS-CoV-2 (57), SARS-CoV (58) and IAV replication (59) and to exert anti-inflammatory and tissue protective effects (60, 61) including in a reovirus-induced myocarditis mouse model (62). Beyond their host-targeted effects, Ac-Leu-Leu-Nle-CHO and aurothioglucose may have direct antiviral effects against

the SARS-CoV-2 M^{pro} or 3C-like proteases, respectively (57, 63). Lastly, josamycin is a natural macrolide antibiotic with an anti-inflammatory activity used in humans in Europe and Japan. Other macrolides have shown anti-IAV and anti-inflammatory activities (64). These findings reveal candidate targets for anti-SARS-CoV-2 approaches. Moreover, they underscore the potential utility of natural products as broad-spectrum antivirals, yet limited scalability typically challenges the use of these products.

Supplemental methods:

Compounds. The Microsource Spectrum, two Biomol and LOPAC libraries were available at the Stanford High-Throughput Bioscience Center. Small molecule inhibitors were purchased from MedchemExpress or Cayman Chemical. Dinaciclib and ribociclib were a gift from Dr. Mardo Koivomagi (Department of Biology, Stanford University, Stanford).

Plasmids. Plasmids used for production of SARS-CoV-2 pseudovirus were a gift from Jing Lin (Vitalant, San Francisco). The rSARS-CoV-2/WT and rSARS-CoV-2/Nluc (rSARS-CoV-2 expressing Nluc-reporter gene) plasmids were generated as previously described (65, 66). Flag-tagged SARS-CoV-2 (2019-nCoV) Spike S1 expression plasmid was purchased from Sino Biological (#VG40591-CF). Plasmid encoding VEEV TC-83 with a nanoluciferase reporter (VEEV TC-83-Cap-nLuc-Tav) was a gift from Dr. William B. Klimstra (Department of Immunology, University of Pittsburgh, Pittsburgh) (67). DENV2 (New Guinea C strain) TSV01 Renilla reporter plasmid (pACYC NGC FL) was a gift from Pei-Yong Shi (Institute for Drug Discovery, University of Texas Medical Branch, Galveston) (68). pDONR223-EGFR, pDONR223-ERBB2, pDONR223-ERBB4 were a gift from William Hahn & David Root (Addgene plasmid #23935, #23888, # 23875) (Broad Institute of Harvard and Massachusetts Institute of Technology, Cambridge) (69). ORFs were recombined into a gateway-compatible pGluc destination vector using Gateway technology (Invitrogen). Mutations were introduced by site-directed mutagenesis using the QuikChange Lightning Site-Directed Mutagenesis Kit (Agilent).

Cells. The African green monkey kidney cell line (Vero E6) constitutively expressing enhanced green fluorescent protein (eGFP) was provided by Dr. Marnix Van Loock (Janssen Pharmaceutica, Beerse, Belgium) (70). Cells were maintained in Dulbecco's modified Eagle's medium (DMEM, Gibco) supplemented with 10% v/v fetal calf serum (Biowest), 0.075% sodium bicarbonate and 1x Pen-strep (Gibco). Vero E6, Vero, Calu-3, HEK-293T, U-87 MG, A549 and BHK-21 cells (ATCC, CRL-1586, CCL-81, HTB-55, CRL-3216, HTB-14, CCL-185, CCL-10) were maintained in DMEM (Corning) supplemented with 10% fetal bovine serum (FBS, Omega Scientific, Inc), 1% L-glutamine 200mM, 1% penicillin-streptomycin, 1% nonessential amino acids, 1% HEPES (Gibco), 1% Sodium pyruvate (Thermofisher scientific). A549-NRP1^{KO} cells (abcam, ab269507) were grown in DMEM:Hams F12 (Cytiva) supplemented with 5% FBS. TMPRSS2-expressing Vero E6 cells (XenoTech, JCRB1819) were maintained in DMEM supplemented with 10% FBS and G418 (1 mg/mL) (Thermofisher, Gibco). All cells were maintained in a humidified incubator with 5% CO_2 at 37°C and tested negative for mycoplasma by MycoAlert (Lonza, Morristown, NJ).

Viral stocks preparation and sequencing. Belgium-GHB-03021 SARS-CoV-2 strain was recovered from a nasopharyngeal swab taken from a patient returning from China early February 2020 (54) and passaged 6 times on Huh7 and Vero E6 cells. 2019-nCoV/USA-WA1/2020 SARS-CoV-2 isolate (NR-52281) (BEI Resources) was passaged 3-6 times in Vero E6-TMPRSS2 cells. The rSARS-CoV-2/WT and rSARS-CoV-2/Nluc (rSARS-CoV-2 expressing Nluc-reporter gene) viral stocks were generated as previously described (55). USA-WA1/2020 from passage 3 used for the majority of the experiments was subject to SARS-CoV-2 whole-genome amplicon-based sequencing on a MiSeq platform (Illumina) by adapting an existing pipeline as described in (56), showing no deletion or point mutations in the multi-basic cleavage (MBC) domain. Belgium/GHB-03021/2020 SARS-CoV-2 from passage 6 was sequenced following a metagenomics pipeline (57) showing 100% deletion of the MBC domain. VEEV-TC-83-nLuc RNA was transcribed in vitro from cDNA plasmid templates linearized with MluI via MEGAscript SP6 kit (Invitrogen #AM1330) and electroporated into BHK-21 cells. DENV RNA was transcribed in vitro from pACYC-DENV2-NGC plasmid by mMessage/mMachine (Ambion) kits and electroporated into BHK-21 cells. WT Trinidad Donkey (TrD) VEEV strain, EBOV (Kikwit isolate) and MARV (Ci67 strain) (BEI Resources) were grown in Vero E6 cells. Supernatants were collected, clarified and stored at -80 °C. Viral titers were determined via plaque assays on BHK-21 (DENV, VEEV) or Vero E6 cells (SARS-CoV-2, EBOV, MARV). MPOXV 2003 (clade II) (NR-2500) and MPOXV 2022 (Lineage B.1, Clade IIb) (NR-58622) were obtained from BEI Resources.

For rVSV-SARS-CoV-2-S production, HEK-293T cells were transfected with spike expression plasmid followed by infection with VSV-G pseudotyped ΔG-luciferase VSV virus and harvesting of culture supernatant, as described (56).

Antibodies. *Antibodies targeting the phosphorylated and total protein forms*: anti-ErbB4 (Santa Cruz, sc-8050), ErbB1, ErbB2, AKT, ERK, p38 (Cell Signaling, #4267, #2242, #4691, #4695, #8690), P-ErbB1 (Tyr1173), P-ErbB2 (Tyr1248), P-ErbB4 (Tyr1284), P-AKT (Ser473), P-ERK (Thr202/Tyr204), P-p38 (Thr180/Tyr182) (Cell Signaling, #4407, #2247, #4757, #4060, #4370, #4511), and β-actin (Sigma-Aldrich, catalog A3854) antibodies.
Co-immunoprecipitation: anti-ErbB1, ErbB2, ErbB4 (Cell Signaling, #4267, #2165, #4795). β-actin (Sigma-Aldrich, catalog A3854), mouse monoclonal anti-Flag® M2-Peroxidase (HRP) (Sigma-Aldrich, A8592)
Flow cytometry: mouse anti-human NRP1-BV421 (Biolegend, 354513), goat anti-human ACE2-APC (R&D systems, FAB933A) and mouse-anti human ErbB2-Alexa Fluor 488 (R&D systems, FAB9589G),

Infection assays and pharmacological inhibition: anti-VACV A33R antibody (BEI Resources, NR-628)

Immunofluorescence: mouse mAb SARS-CoV-2 nucleocapsid antibody (SinoBiological, #40143-MM05), DAPI (ThermoFisher, D3571) and Alexa Fluor™ 594 Phalloidin (ThermoFisher, #A12381)Claudin 7 polyclonal antibody (ThermoFisher, #34-9100) Rab7 (Origene, #AB0033-200)

siRNAs. ON-TARGETPlus siRNA SMARTpools against 7 genes and non-targeting siRNA (siNT) were purchased from Dharmacon/Horizon Discovery with gene IDs as follows: EGFR (1956), ErbB2 (2064), ErbB4 (2066), RIPK2 (8767), RAF1 (5894), STK10 (6793), MAP2K5 (5607).

In vitro kinase assays. These assays were performed on the LabChip platform (Nanosyn) or radiometric HotSpot™ platform (Reaction Biology).

Sequences of primers used for RT-qPCR.

GAPDH (F- GTCTCCTCTGACTTCAACAGCG; R- ACCACCCTGTTGCTGTAGCCAA), ErbB1 (F- AACACCCTGGTCTGGAAGTACG; R- TCGTTGGACAGCCTTCAAGACC), ErbB2 (F- GGAAGTACACGATGCGGAGACT; R- TACCTTCCTCAGCTCCGTCTCTT), ErbB4 (F- GGAGTATGTCCACGAGCACAAG; R- CGAGTCGTCTTTCTTCCAGGTAC), SARS-CoV2-N (F- AAGCTGGACTTCCCTATGGTG; R- CGATTGCAGCATTGTTAGCAGG).

Supplemental references

1. Novartis. Tykerb (U.S. package insert). 2018.

2. Pivot X, Manikhas A, Żurawski B, Chmielowska E, Karaszewska B, Allerton R, et al. CEREBEL (EGF111438): A Phase III, Randomized, Open-Label Study of Lapatinib Plus Capecitabine Versus Trastuzumab Plus Capecitabine in Patients With Human Epidermal Growth Factor Receptor 2-Positive Metastatic Breast Cancer. *J Clin Oncol.* 2015;33(14):1564-73.

3. Schwartzberg LS, Franco SX, Florance A, O'Rourke L, Maltzman J, and Johnston S. Lapatinib plus letrozole as first-line therapy for HER-2+ hormone receptor-positive metastatic breast cancer. *The oncologist.* 2010;15(2):122-9.

4. Blackwell KL, Pegram MD, Tan-Chiu E, Schwartzberg LS, Arbushites MC, Maltzman JD, et al. Single-agent lapatinib for HER2-overexpressing advanced or metastatic breast cancer that progressed on first- or second-line trastuzumab-containing regimens. *Ann Oncol.* 2009;20(6):1026-31.

5. Blackwell KL, Burstein HJ, Storniolo AM, Rugo H, Sledge G, Koehler M, et al. Randomized study of Lapatinib alone or in combination with trastuzumab in women with ErbB2-positive, trastuzumab-refractory metastatic breast cancer. *J Clin Oncol.* 2010;28(7):1124-30.

6. Burris HA, 3rd, Hurwitz HI, Dees EC, Dowlati A, Blackwell KL, O'Neil B, et al. Phase I safety, pharmacokinetics, and clinical activity study of lapatinib (GW572016), a reversible dual inhibitor of epidermal growth factor receptor tyrosine kinases, in heavily pretreated patients with metastatic carcinomas. *J Clin Oncol.* 2005;23(23):5305-13.

7. Burstein HJ, Storniolo AM, Franco S, Forster J, Stein S, Rubin S, et al. A phase II study of lapatinib monotherapy in chemotherapy-refractory HER2-positive and HER2-negative advanced or metastatic breast cancer. *Ann Oncol.* 2008;19(6):1068-74.

8. Gomez HL, Doval DC, Chavez MA, Ang PC-S, Aziz Z, Nag S, et al. Efficacy and Safety of Lapatinib As First-Line Therapy for ErbB2-Amplified Locally Advanced or Metastatic Breast Cancer. *Journal of Clinical Oncology.* 2008;26(18):2999-3005.

9. Hurvitz SA, and Kakkar R. Role of lapatinib alone or in combination in the treatment of HER2-positive breast cancer. *Breast Cancer (Dove Med Press).* 2012;4:35-51.

10. Perez EA, Koehler M, Byrne J, Preston AJ, Rappold E, and Ewer MS. Cardiac safety of lapatinib: pooled analysis of 3689 patients enrolled in clinical trials. *Mayo Clinic proceedings.* 2008;83(6):679-86.

11. Toi M, Iwata H, Fujiwara Y, Ito Y, Nakamura S, Tokuda Y, et al. Lapatinib monotherapy in patients with relapsed, advanced, or metastatic breast cancer: efficacy, safety, and biomarker results from Japanese patients phase II studies. *British journal of cancer.* 2009;101(10):1676-82.

12. Piccart-Gebhart M, Holmes E, Baselga J, de Azambuja E, Dueck AC, Viale G, et al. Adjuvant Lapatinib and Trastuzumab for Early Human Epidermal Growth Factor Receptor 2-Positive Breast Cancer: Results From the Randomized Phase III Adjuvant Lapatinib and/or Trastuzumab Treatment Optimization Trial. *J Clin Oncol.* 2016;34(10):1034-42.

13. Kloth JSL, Pagani A, Verboom MC, Malovini A, Napolitano C, Kruit WHJ, et al. Incidence and relevance of QTc-interval prolongation caused by tyrosine kinase inhibitors. *British journal of cancer.* 2015;112(6):1011-6.

14. Dogan E, Yorgun H, Petekkaya I, Ozer N, Altundag K, and Ozisik Y. Evaluation of cardiac safety of lapatinib therapy for ErbB2-positive metastatic breast cancer: a single center experience. *Medical oncology (Northwood, London, England).* 2012;29(5):3232-9.

15. Hackshaw MD, Danysh HE, Singh J, Ritchey ME, Ladner A, Taitt C, et al. Incidence of pneumonitis/interstitial lung disease induced by HER2-targeting therapy for HER2-positive metastatic breast cancer. *Breast cancer research and treatment.* 2020;183(1):23-39.

16. Jagiello-Gruszfeld A, Tjulandin S, Dobrovolskaya N, Manikhas A, Pienkowski T, DeSilvio M, et al. A single-arm phase II trial of first-line paclitaxel in combination with lapatinib in HER2-overexpressing metastatic breast cancer. *Oncology.* 2010;79(1-2):129-35.

17. Capri G, Chang J, Chen SC, Conte P, Cwiertka K, Jerusalem G, et al. An open-label expanded access study of lapatinib and capecitabine in patients with HER2-overexpressing locally advanced or metastatic breast cancer. *Ann Oncol.* 2010;21(3):474-80.

18. Xu B-H, Jiang Z-F, Chua D, Shao Z-M, Luo R-C, Wang X-J, et al. Lapatinib plus capecitabine in treating HER2-positive advanced breast cancer: efficacy, safety, and biomarker results from Chinese patients. *Chin J Cancer.* 2011;30(5):327-35.

19. Bates CA, Zhao B, Schlobohm A, Asquith C, Zuercher W, and Barkauskas C. 2019.

20. Brenner T, Motsch J, Werner J, Grenacher L, Martin E, and Hofer S. Rapid-Onset Acute Respiratory Distress Syndrome (ARDS) in a Patient Undergoing Metastatic Liver Resection: A Case Report and Review of the Literature. *Anesthesiol Res Pract.* 2010;2010:586425.

21. Chan AK, Choo BA, and Glaholm J. Pulmonary toxicity with oxaliplatin and capecitabine/5-Fluorouracil chemotherapy: a case report and review of the literature. *Onkologie.* 2011;34(8-9):443-6.

22. Torrisi JM, Schwartz LH, Gollub MJ, Ginsberg MS, Bosl GJ, and Hricak H. CT findings of chemotherapy-induced toxicity: what radiologists need to know about the clinical and radiologic manifestations of chemotherapy toxicity. *Radiology.* 2011;258(1):41-56.

23. Bielopolski D, Evron E, Moreh-Rahav O, Landes M, Stemmer SM, and Salamon F. Paclitaxel-induced pneumonitis in patients with breast cancer: case series and review of the literature. *Journal of chemotherapy (Florence, Italy).* 2017;29(2):113-7.

24. Asquith CRM, Laitinen T, Bennett JM, Wells CI, Elkins JM, Zuercher WJ, et al. Design and Analysis of the 4-Anilinoquin(az)oline Kinase Inhibition Profiles of GAK/SLK/STK10 Using Quantitative Structure-Activity Relationships. *ChemMedChem.* 2020;15(1):26-49.

25. Tabara H, Naito Y, Ito A, Katsuma A, Sakurai MA, Ohno S, et al. Neonatal lethality in knockout mice expressing the kinase-dead form of the gefitinib target GAK is caused by pulmonary dysfunction. *PLoS One.* 2011;6(10):e26034.

26. Bates CA, Zhao B, Schlobohm A, Asquith C, Zuercher W, and Barkauskas CE. *A61 EPITHELIAL BIOLOGY*. American Thoracic Society; 2019:A2125-A.

27. Group TWREAfC-TW. Association Between Administration of Systemic Corticosteroids and Mortality Among Critically Ill Patients With COVID-19: A Meta-analysis. *JAMA.* 2020;324(13):1330-41.

28. Dequin P-F, Heming N, Meziani F, Plantefève G, Voiriot G, Badié J, et al. Effect of Hydrocortisone on 21-Day Mortality or Respiratory Support Among Critically Ill Patients With COVID-19: A Randomized Clinical Trial. *JAMA.* 2020;324(13):1298-306.

29. Investigators TWCftR-C. Effect of Hydrocortisone on Mortality and Organ Support in Patients With Severe COVID-19: The REMAP-CAP COVID-19 Corticosteroid Domain Randomized Clinical Trial. *JAMA.* 2020;324(13):1317-29.

30. Neveu G, Barouch-Bentov R, Ziv-Av A, Gerber D, Jacob Y, and Einav S. Identification and Targeting of an Interaction between a Tyrosine Motif within Hepatitis C Virus Core Protein and AP2M1 Essential for Viral Assembly. *PLoS pathogens.* 2012;8(8):e1002845.

31. Bekerman E, Neveu G, Shulla A, Brannan J, Pu S-Y, Wang S, et al. Anticancer kinase inhibitors impair intracellular viral trafficking and exert broad-spectrum antiviral effects. *The Journal of clinical investigation.* 2017;127(4).

32. Pu S, Schor S, Karim M, Saul S, Robinson M, Kumar S, et al. BIKE regulates dengue virus infection and is a cellular target for broad-spectrum antivirals. *Antiviral Res.* 2020;184:104966.

33. Wang P-G, Tang D-J, Hua Z, Wang Z, and An J. Sunitinib reduces the infection of SARS-CoV, MERS-CoV and SARS-CoV-2 partially by inhibiting AP2M1 phosphorylation. *Cell Discovery.* 2020;6(1):71.

34. Karim M, Saul S, Ghita L, Sahoo MK, Ye C, Bhalla N, et al. Numb-associated kinases are required for SARS-CoV-2 infection and are cellular targets for antiviral strategies. *Antiviral Res.* 2022;204:105367.

35. Kumar N, Liang Y, Parslow TG, and Liang Y. Receptor Tyrosine Kinase Inhibitors Block Multiple Steps of Influenza A Virus Replication. *Journal of virology.* 2011;85(6):2818-27.

36. Kumar N, Sharma NR, Ly H, Parslow TG, and Liang Y. Receptor tyrosine kinase inhibitors that block replication of influenza a and other viruses. *Antimicrobial agents and chemotherapy.* 2011;55(12):5553-9.

37. Zoeller RA, and Geoghegan-Barek K. A cell-based high-throughput screen identifies tyrphostin AG 879 as an inhibitor of animal cell phospholipid and fatty acid biosynthesis. *Biochemistry and Biophysics Reports.* 2019;18:100621.

38. Kwan CY, and Achike FI. Tetrandrine and related bis-benzylisoquinoline alkaloids from medicinal herbs: cardiovascular effects and mechanisms of action. *Acta pharmacologica Sinica.* 2002;23(12):1057-68.

39. Sakurai Y, Kolokoltsov AA, Chen CC, Tidwell MW, Bauta WE, Klugbauer N, et al. Ebola virus. Two-pore channels control Ebola virus host cell entry and are drug targets for disease treatment. *Science.* 2015;347(6225):995-8.

40. Onishi E, Natori K, and Yamazaki S. The antiviral effect of phorbol ester and calcium ionophore A23187 is not mediated by interferons. *Journal of interferon research.* 1991;11(3):171-5.

41. Marois I, Cloutier A, Meunier I, Weingartl HM, Cantin AM, and Richter MV. Inhibition of Influenza Virus Replication by Targeting Broad Host Cell Pathways. *PLOS ONE.* 2014;9(10):e110631.

42. Geller R, Taguwa S, and Frydman J. Broad action of Hsp90 as a host chaperone required for viral replication. *Biochimica et biophysica acta.* 2012;1823(3):698-706.

43. Amraiz D ZN, Fatima M. Antiviral evaluation of an Hsp90 inhibitor, gedunin, against dengue virus. . *Trop J Pharm Res.* 2017;16(5):997-1004.

44. Vrijsen R, Vanden Berghe DA, Vlietinck AJ, and Boeyé A. Lycorine: a eukaryotic termination inhibitor? *The Journal of biological chemistry.* 1986;261(2):505-7.

45. Yang L, Zhang JH, Zhang XL, Lao GJ, Su GM, Wang L, et al. Tandem mass tag-based quantitative proteomic analysis of lycorine treatment in highly pathogenic avian influenza H5N1 virus infection. *PeerJ.* 2019;7:e7697-e.

46. Ieven M, van den Berghe DA, and Vlietinck AJ. Plant antiviral agents. IV. Influence of lycorine on growth pattern of three animal viruses. *Planta medica.* 1983;49(2):109-14.

47. Szlávik L, Gyuris A, Minárovits J, Forgo P, Molnár J, and Hohmann J. Alkaloids from Leucojum vernum and antiretroviral activity of Amaryllidaceae alkaloids. *Planta medica.* 2004;70(9):871-3.

48. Li SY, Chen C, Zhang HQ, Guo HY, Wang H, Wang L, et al. Identification of natural compounds with antiviral activities against SARS-associated coronavirus. *Antiviral Res.* 2005;67(1):18-23.

49. Liu J, Yang Y, Xu Y, Ma C, Qin C, and Zhang L. Lycorine reduces mortality of human enterovirus 71-infected mice by inhibiting virus replication. *Virology journal.* 2011;8:483.

50. Li S, Liu X, Chen X, and Bi L. Research Progress on Anti-Inflammatory Effects and Mechanisms of Alkaloids from Chinese Medical Herbs. *Evidence-Based Complementary and Alternative Medicine.* 2020;2020:1303524.

51. Rajapakse AG, Ming XF, Carvas JM, and Yang Z. The hexosamine biosynthesis inhibitor azaserine prevents endothelial inflammation and dysfunction under hyperglycemic condition through antioxidant effects. *American journal of physiology Heart and circulatory physiology.* 2009;296(3):H815-22.

52. Botz B, Bölcskei K, Kereskai L, Kovács M, Németh T, Szigeti K, et al. Differential regulatory role of pituitary adenylate cyclase-activating polypeptide in the serum-transfer arthritis model. *Arthritis & rheumatology (Hoboken, NJ).* 2014;66(10):2739-50.

53. Sasaki T, Kishi M, Saito M, Tanaka T, Higuchi N, Kominami E, et al. Inhibitory effect of di- and tripeptidyl aldehydes on calpains and cathepsins. *Journal of enzyme inhibition.* 1990;3(3):195-201.

54. Schneider M, Ackermann K, Stuart M, Wex C, Protzer U, Schätzl HM, et al. Severe acute respiratory syndrome coronavirus replication is severely impaired by MG132 due to proteasome-independent inhibition of M-calpain. *J Virol.* 2012;86(18):10112-22.

55. Upla P, Marjomäki V, Nissinen L, Nylund C, Waris M, Hyypiä T, et al. Calpain 1 and 2 are required for RNA replication of echovirus 1. *J Virol.* 2008;82(3):1581-90.

56. Zheng K, Xiang Y, Wang Q, Jin F, Chen M, Ma K, et al. Calcium-signal facilitates herpes simplex virus type 1 nuclear transport through slingshot 1 and calpain-1 activation. *Virus research.* 2014;188:32-7.

57. Ma C, Sacco MD, Hurst B, Townsend JA, Hu Y, Szeto T, et al. Boceprevir, GC-376, and calpain inhibitors II, XII inhibit SARS-CoV-2 viral replication by targeting the viral main protease. *Cell research.* 2020;30(8):678-92.

58. Barnard DL, Hubbard VD, Burton J, Smee DF, Morrey JD, Otto MJ, et al. Inhibition of severe acute respiratory syndrome-associated coronavirus (SARSCoV) by calpain inhibitors and beta-D-N4-hydroxycytidine. *Antiviral chemistry & chemotherapy.* 2004;15(1):15-22.

59. Blanc F, Furio L, Moisy D, Yen HL, Chignard M, Letavernier E, et al. Targeting host calpain proteases decreases influenza A virus infection. *American journal of physiology Lung cellular and molecular physiology.* 2016;310(7):L689-99.

60. Li X, Li Y, Shan L, Shen E, Chen R, and Peng T. Over-expression of calpastatin inhibits calpain activation and attenuates myocardial dysfunction during endotoxaemia. *Cardiovascular research.* 2009;83(1):72-9.

61. Supinski GS, and Callahan LA. Calpain activation contributes to endotoxin-induced diaphragmatic dysfunction. *American journal of respiratory cell and molecular biology.* 2010;42(1):80-7.

62. DeBiasi RL, Edelstein CL, Sherry B, and Tyler KL. Calpain inhibition protects against virus-induced apoptotic myocardial injury. *J Virol.* 2001;75(1):351-61.

63. Baker JD, Uhrich RL, Kraemer GC, Love JE, and Kraemer BC. A drug repurposing screen identifies hepatitis C antivirals as inhibitors of the SARS-CoV2 main protease. *PLOS ONE.* 2021;16(2):e0245962.

64. Sugamata R, Sugawara A, Nagao T, Suzuki K, Hirose T, Yamamoto K, et al. Leucomycin A3, a 16-membered macrolide antibiotic, inhibits influenza A virus infection and disease progression. *The Journal of antibiotics.* 2014;67(3):213-22.

65. Chiem K, Morales Vasquez D, Park J-G, Platt RN, Anderson T, Walter MR, et al. Generation and Characterization of Recombinant SARS-CoV-2 Expressing Reporter Genes. *Journal of virology.* 2021;95(7):e02209-20.

66. Chiem K, Ye C, and Martinez-Sobrido L. Generation of Recombinant SARS-CoV-2 Using a Bacterial Artificial Chromosome. *Current protocols in microbiology.* 2020;59(1):e126.

67. Sun C, Gardner CL, Watson AM, Ryman KD, and Klimstra WB. Stable, high-level expression of reporter proteins from improved alphavirus expression vectors to track replication and dissemination during encephalitic and arthritogenic disease. *Journal of virology.* 2014;88(4):2035-46.

68. Zou G, Xu HY, Qing M, Wang Q-Y, and Shi P-Y. Development and characterization of a stable luciferase dengue virus for high-throughput screening. *Antiviral research.* 2011;91(1):11-9.

69. Johannessen CM, Boehm JS, Kim SY, Thomas SR, Wardwell L, Johnson LA, et al. COT drives resistance to RAF inhibition through MAP kinase pathway reactivation. *Nature.* 2010;468(7326):968-72.

70. Ivens T, Eynde CVd, Acker KV, Nijs E, Dams G, Bettens E, et al. Development of a homogeneous screening assay for automated detection of antiviral agents active against severe acute respiratory syndrome-associated coronavirus. *Journal of virological methods.* 2005;129(1):56-63.

Figure 4E

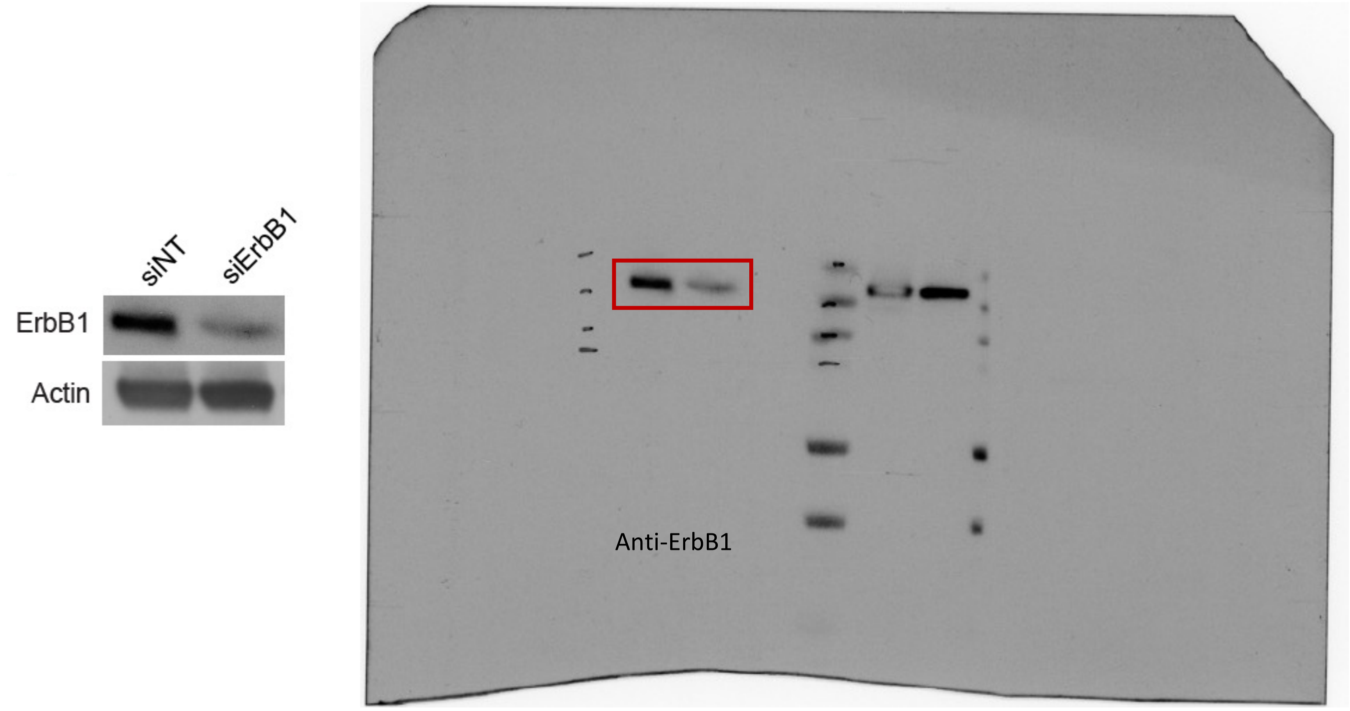
siNT
siErbB1
ErbB1
Actin
Anti-ErbB1

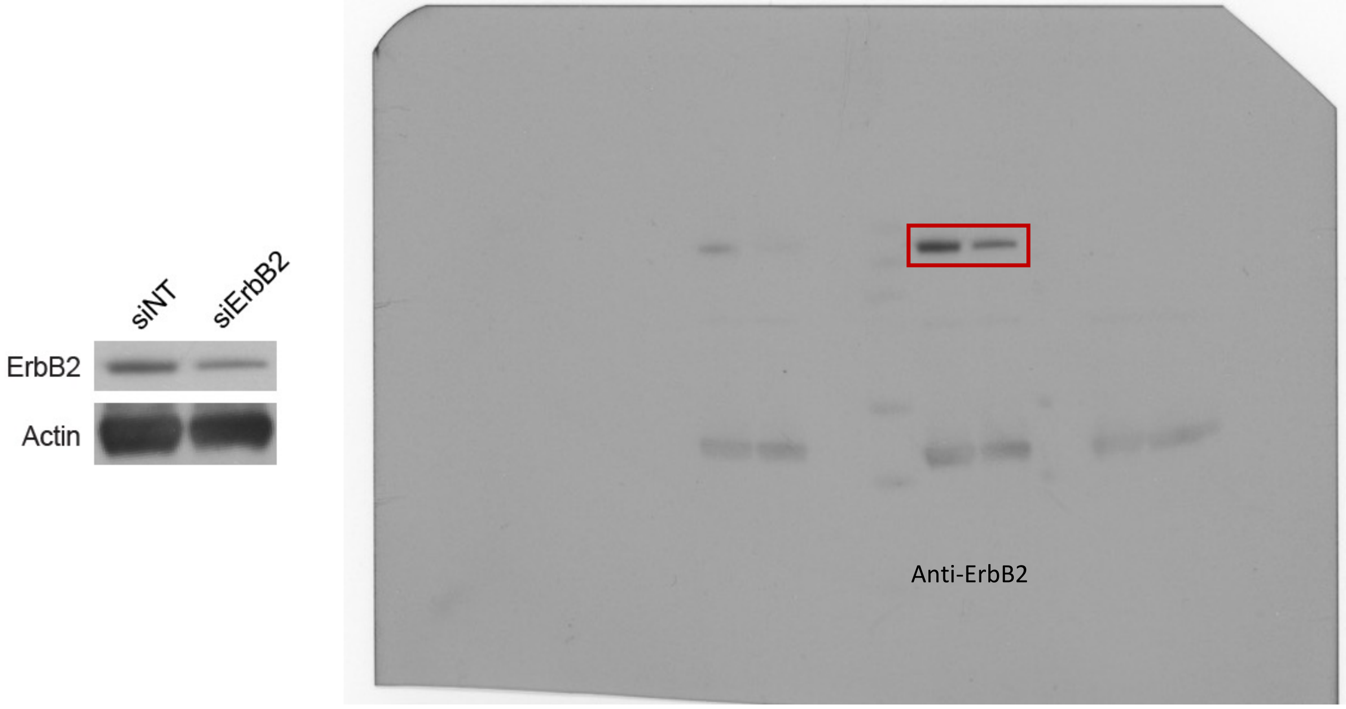
siNT
siErbB2
ErbB2
Actin
Anti-ErbB2

Figure 4E

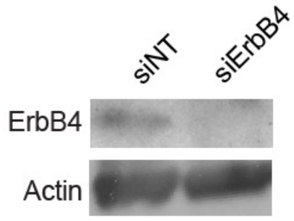

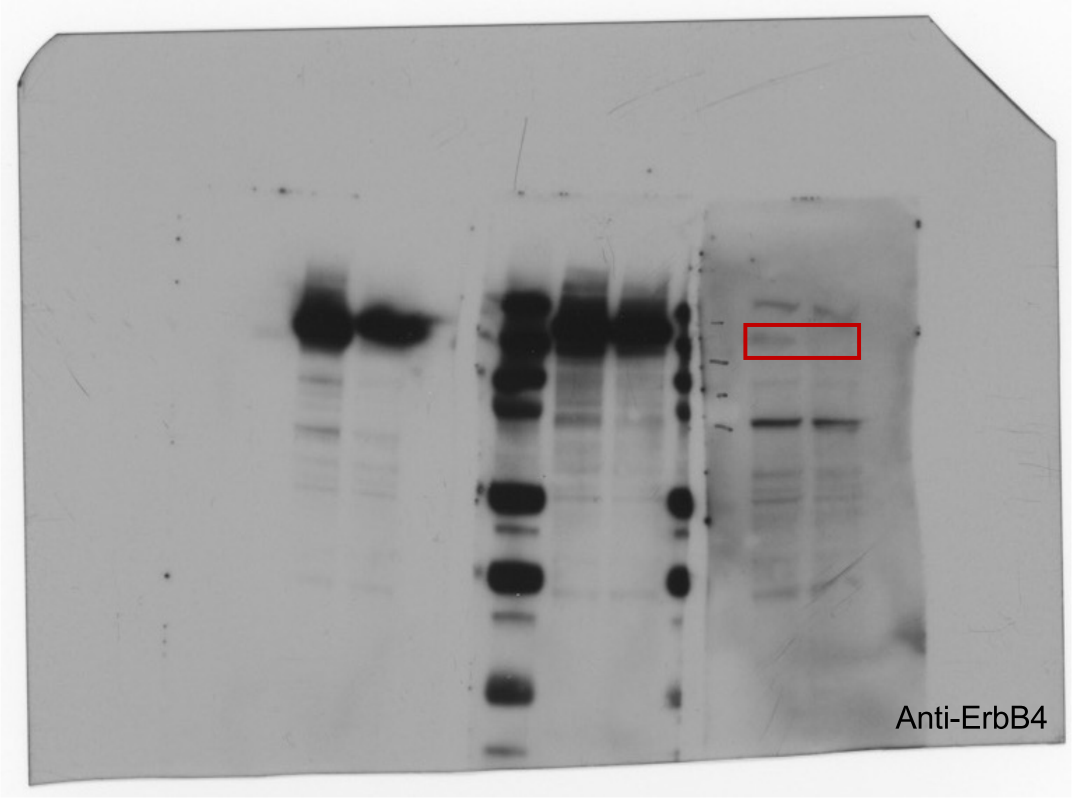

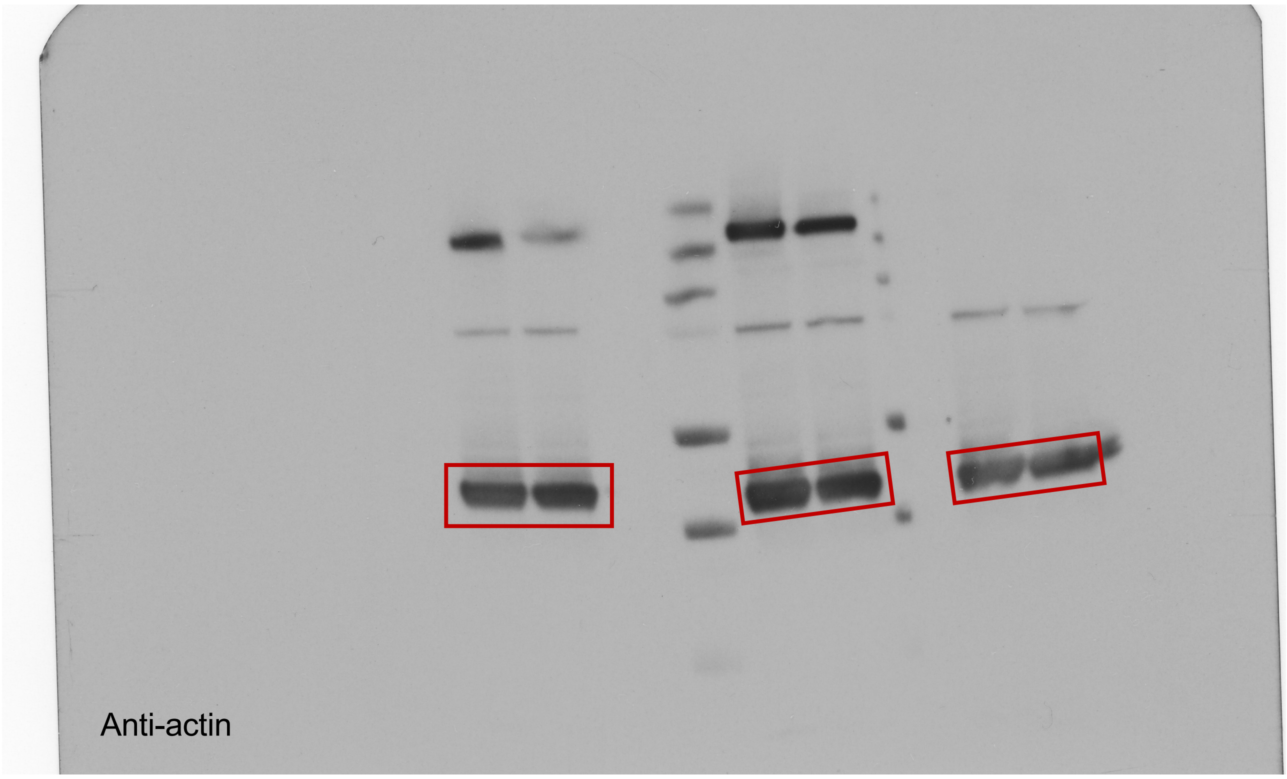

Figure 4F

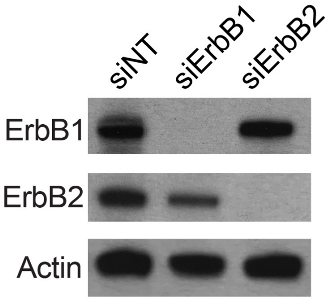
siNT
siErbB1
siErbB2
ErbB1
ErbB2
Actin

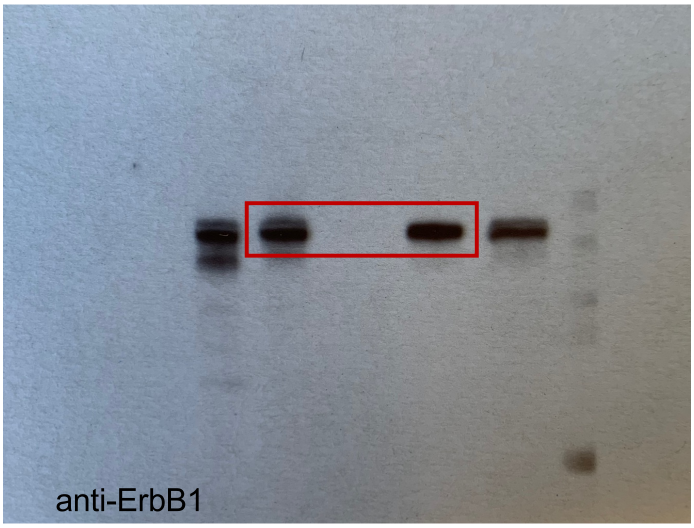
anti-ErbB1

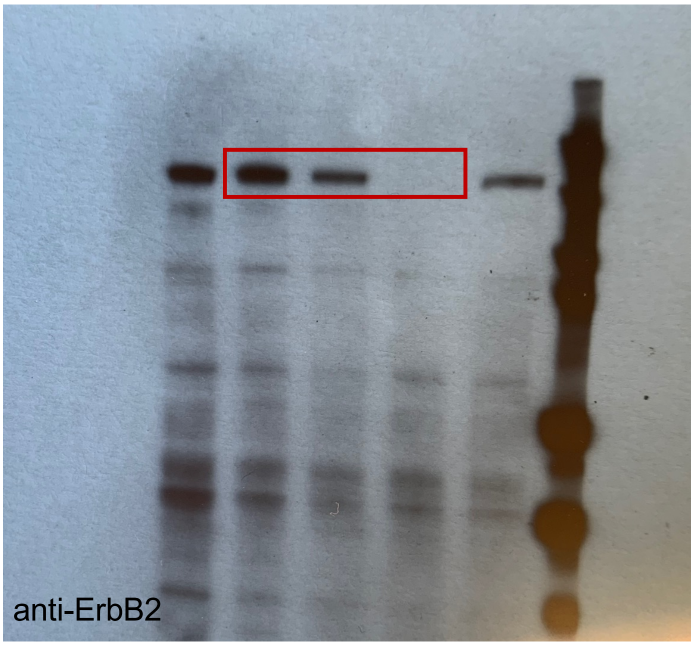
anti-ErbB2

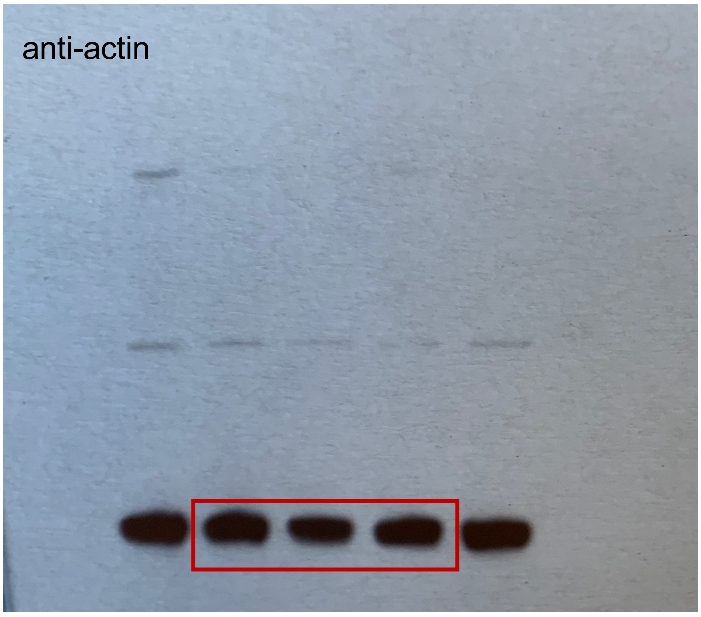
anti-actin

Figure 5M

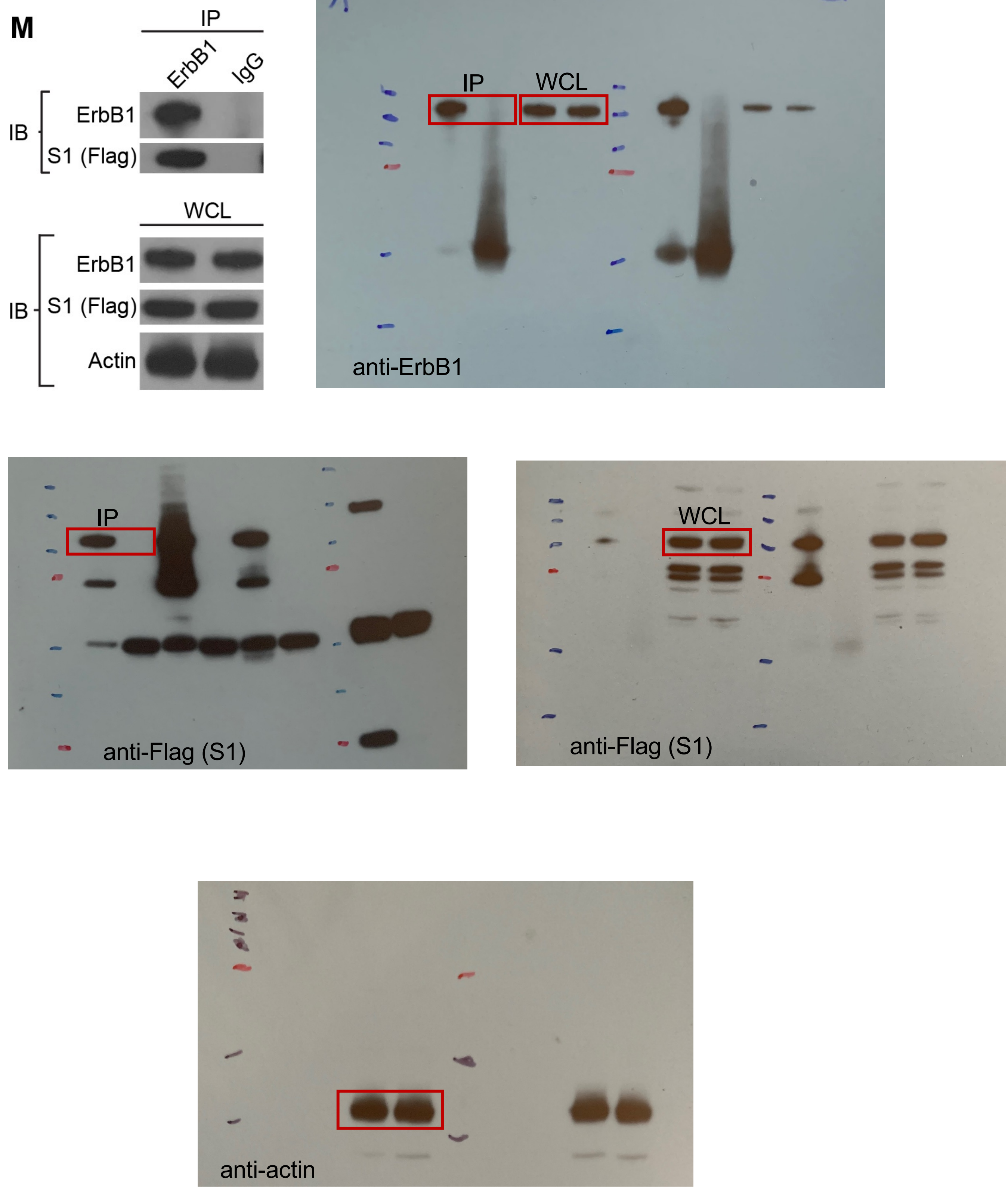
M
IP
ErbB1
IgG
IB
ErbB1
S1 (Flag)
WCL
IB
ErbB1
S1 (Flag)
Actin
1
2.
IP
WCL
anti-ErbB1
IP
anti-Flag (S1)
WCL
anti-Flag (S1)
anti-actin

Figure 5M

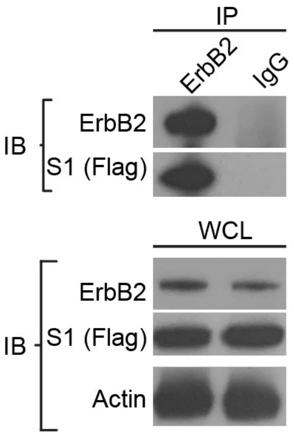
IP
ErbB2
IgG
IB
ErbB2
S1 (Flag)
WCL
IB
ErbB2
S1 (Flag)
Actin

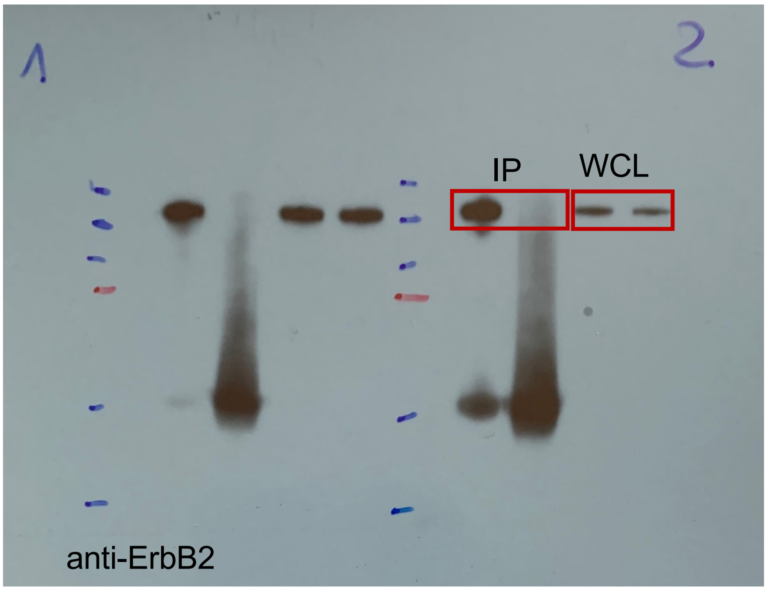
1
2
IP
WCL
anti-ErbB2

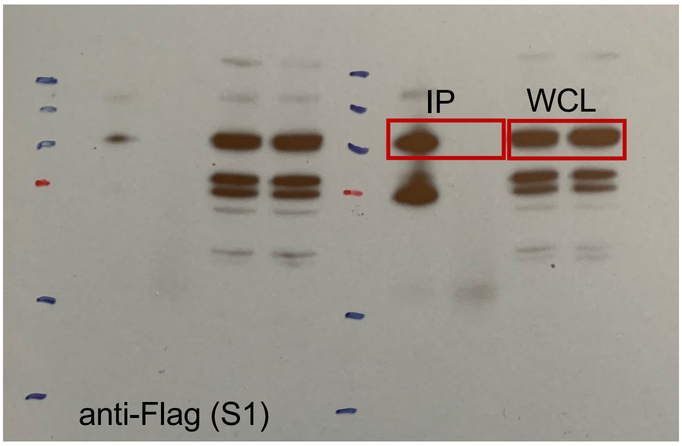
IP
WCL
anti-Flag (S1)

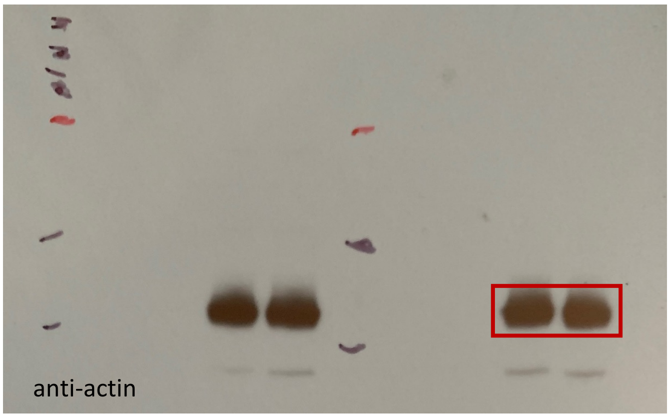
anti-actin

Figure 5M

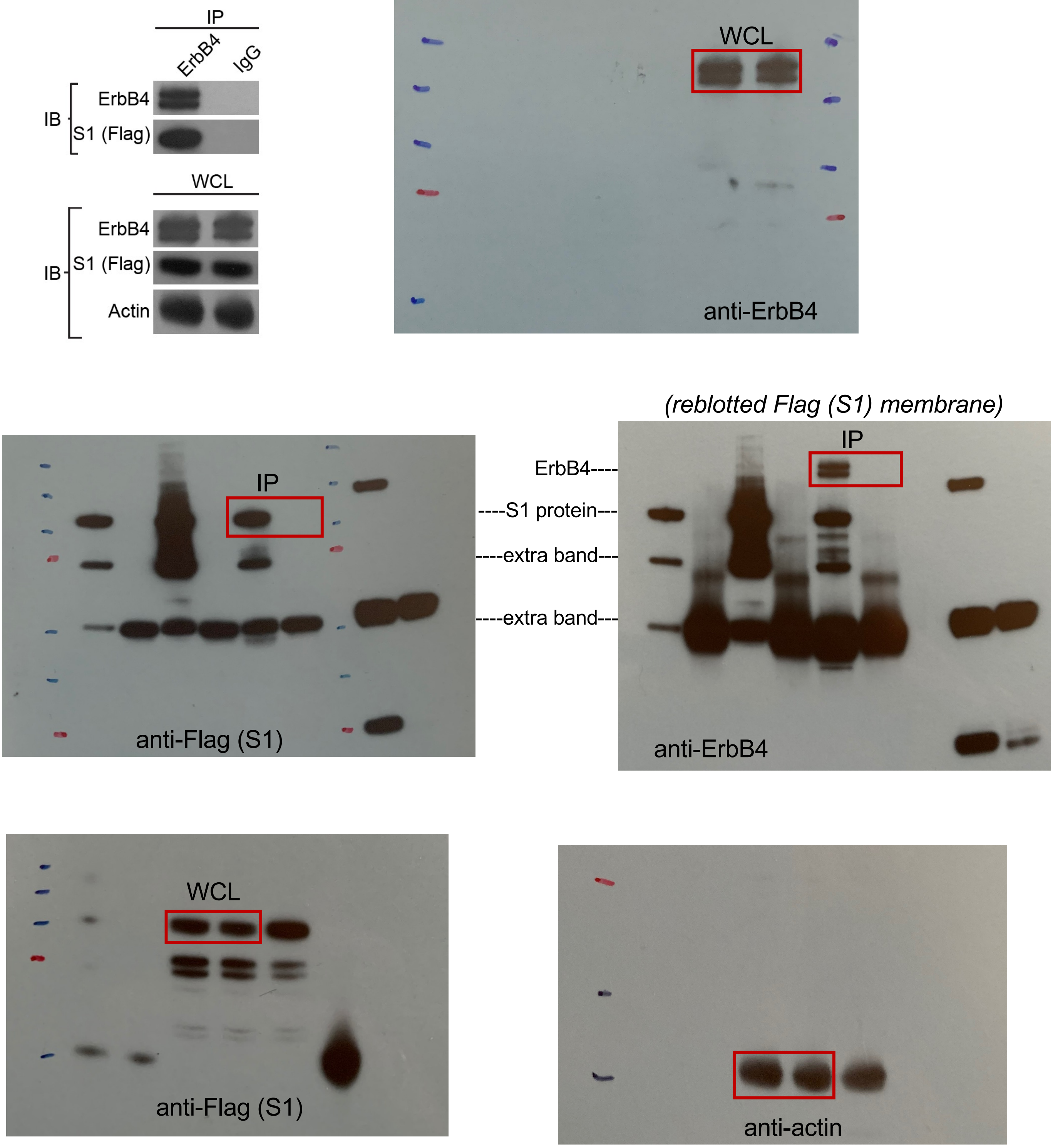

IP
ErbB4
IgG
IB
ErbB4
S1 (Flag)
WCL
IB
ErbB4
S1 (Flag)
Actin
WCL
anti-ErbB4
IP
anti-Flag (S1)
(reblotted Flag (S1) membrane)
IP
ErbB4----
----S1 protein---
----extra band---
----extra band---
anti-ErbB4
WCL
anti-Flag (S1)
anti-actin

All membranes were cropped prior to blotting to facilitate staining with multiple antibodies. Numbers indicate parts originating from the same membrane. The membranes were blotted first for phosphoproteins (or nucleocapsid), stripped and then blotted for the corresponding total protein. Same volume of sample was loaded for all four membranes. A representative actin is shown. The quantification was done using actin corresponding to each membrane.

Figure 6A

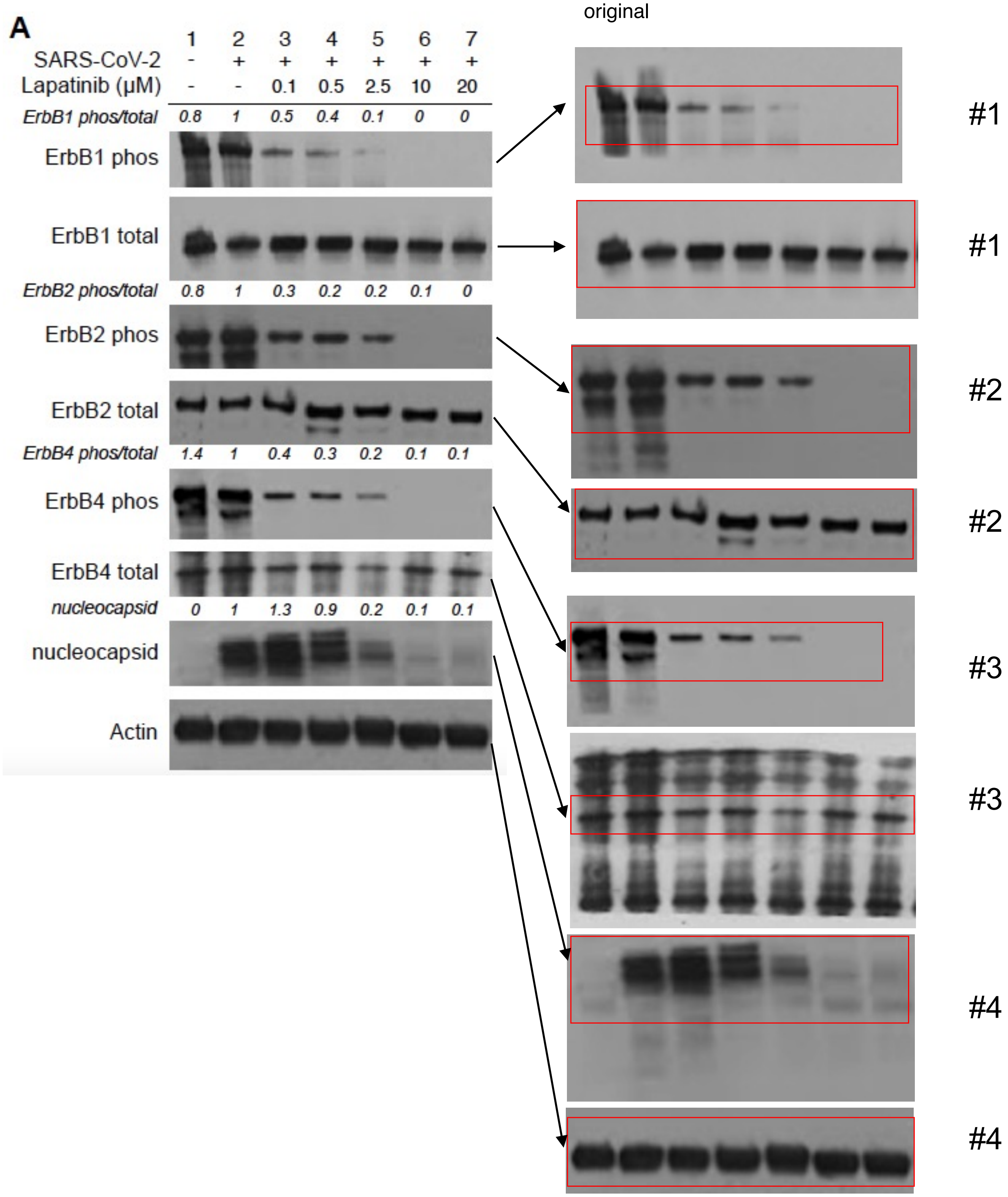

Figure 6D

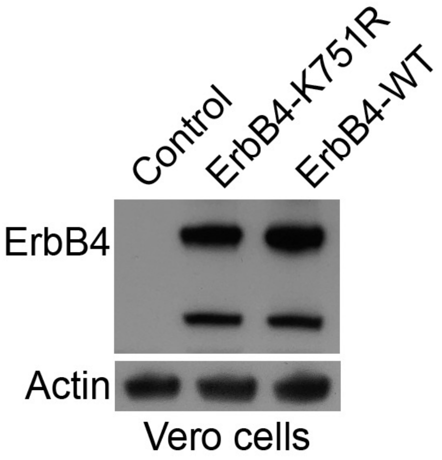

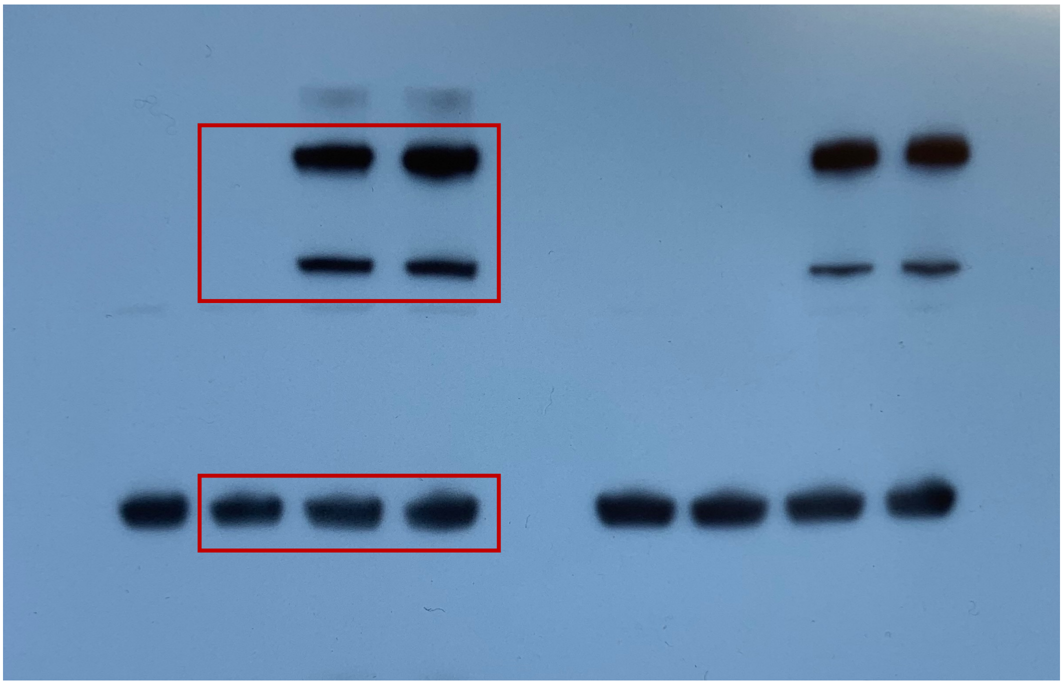

All membranes were cropped prior to blotting to facilitate staining with multiple antibodies. Numbers in Fig. 6F and Fig 7A, B indicate parts originating from the same membrane. The membranes were blotted first for phosphoproteins, stripped and then blotted for the corresponding total protein.

Figure 6F

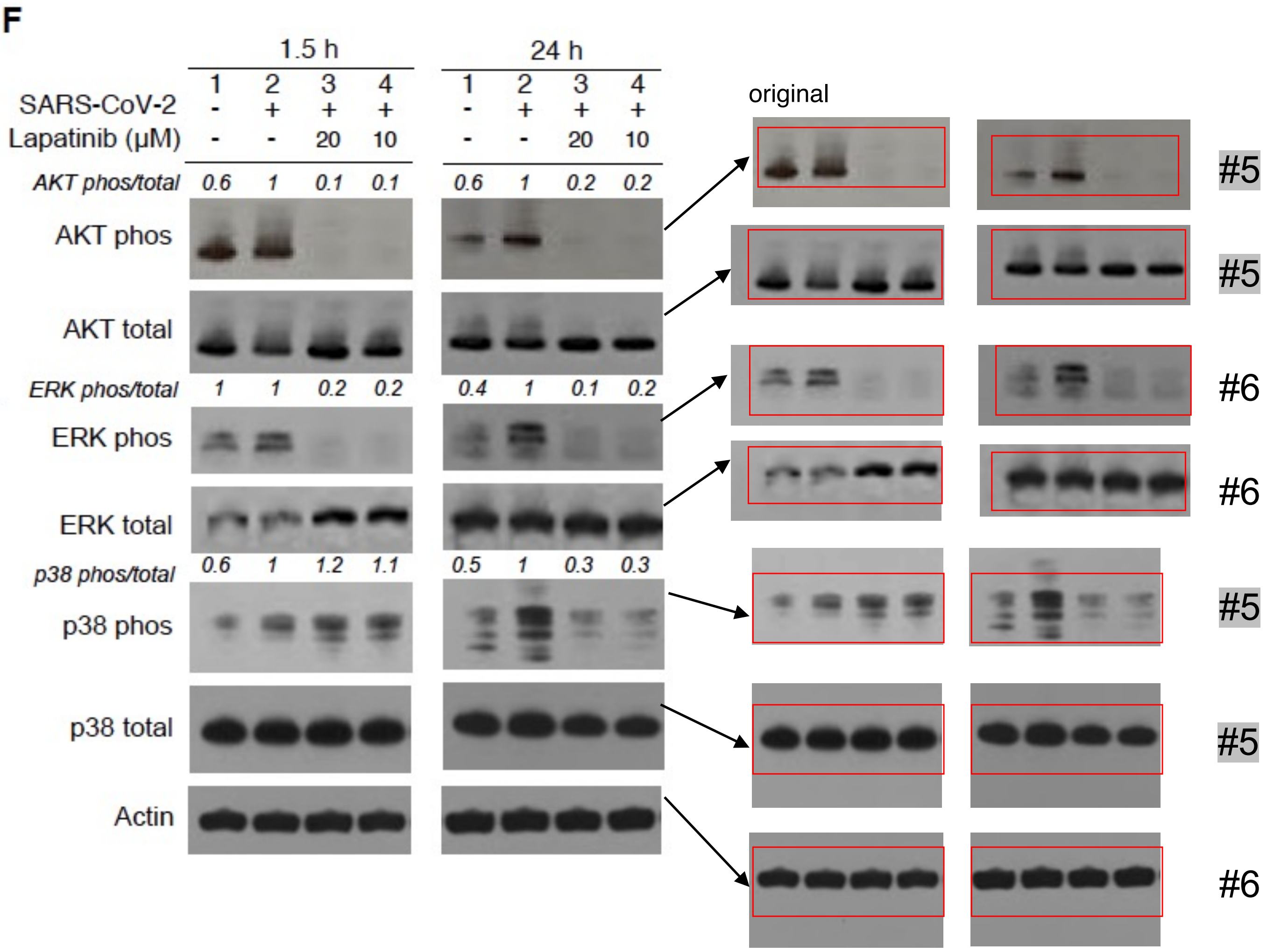

All membranes were cropped prior to blotting to facilitate staining with multiple antibodies. Numbers in Fig. 6F and Fig 7A, B indicate parts originating from the same membrane. The membranes were blotted first for phosphoproteins, stripped and then blotted for the corresponding total protein.

Supplemental Figure 7A, B

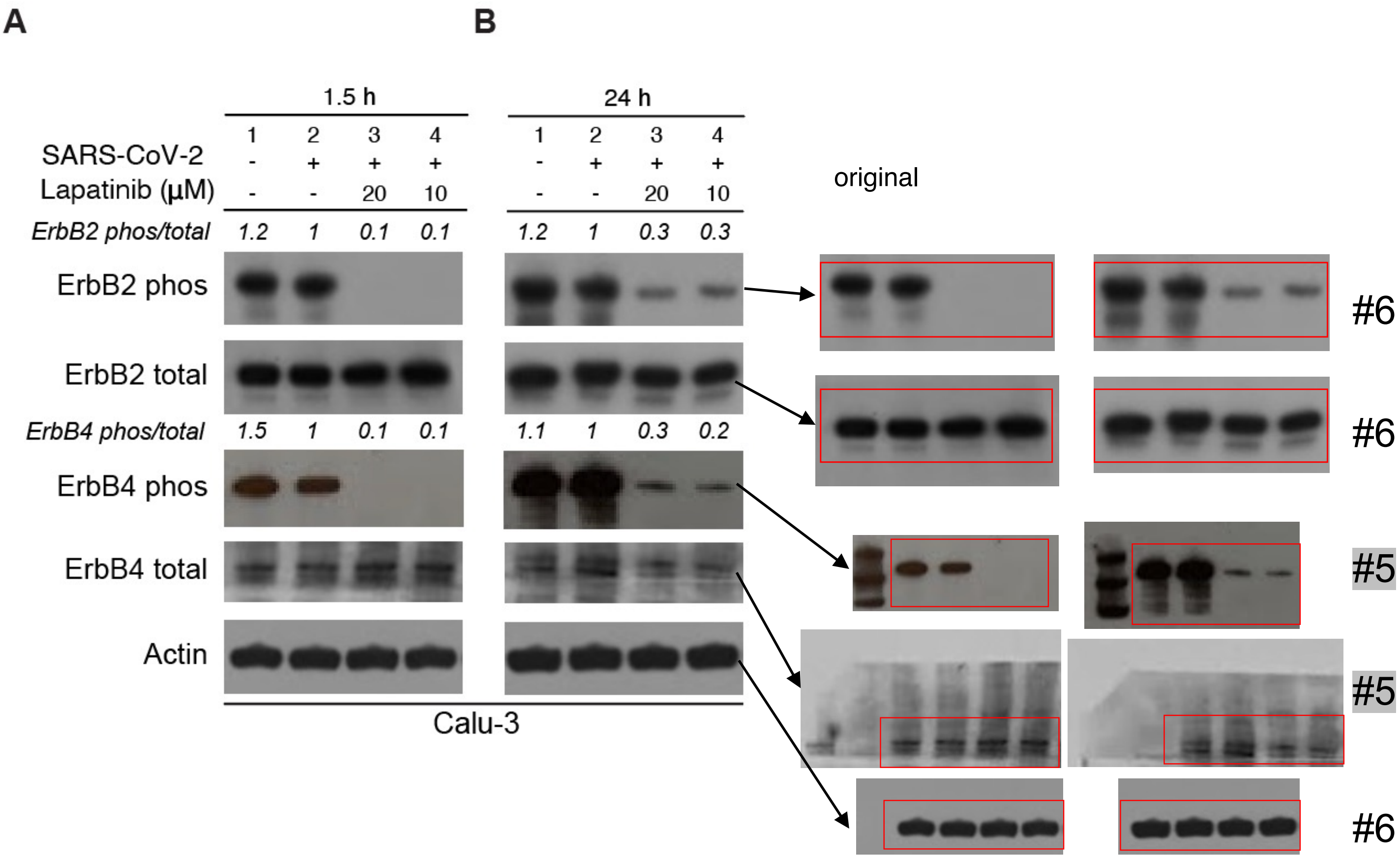

All membranes were cropped prior to blotting to facilitate staining with multiple antibodies. Numbers in Fig. 7C and Fig 7I represent parts originating from the same membrane. The membranes were blotted first for phosphoproteins, stripped and then blotted for the corresponding total protein.

Supplemental Figure 7C

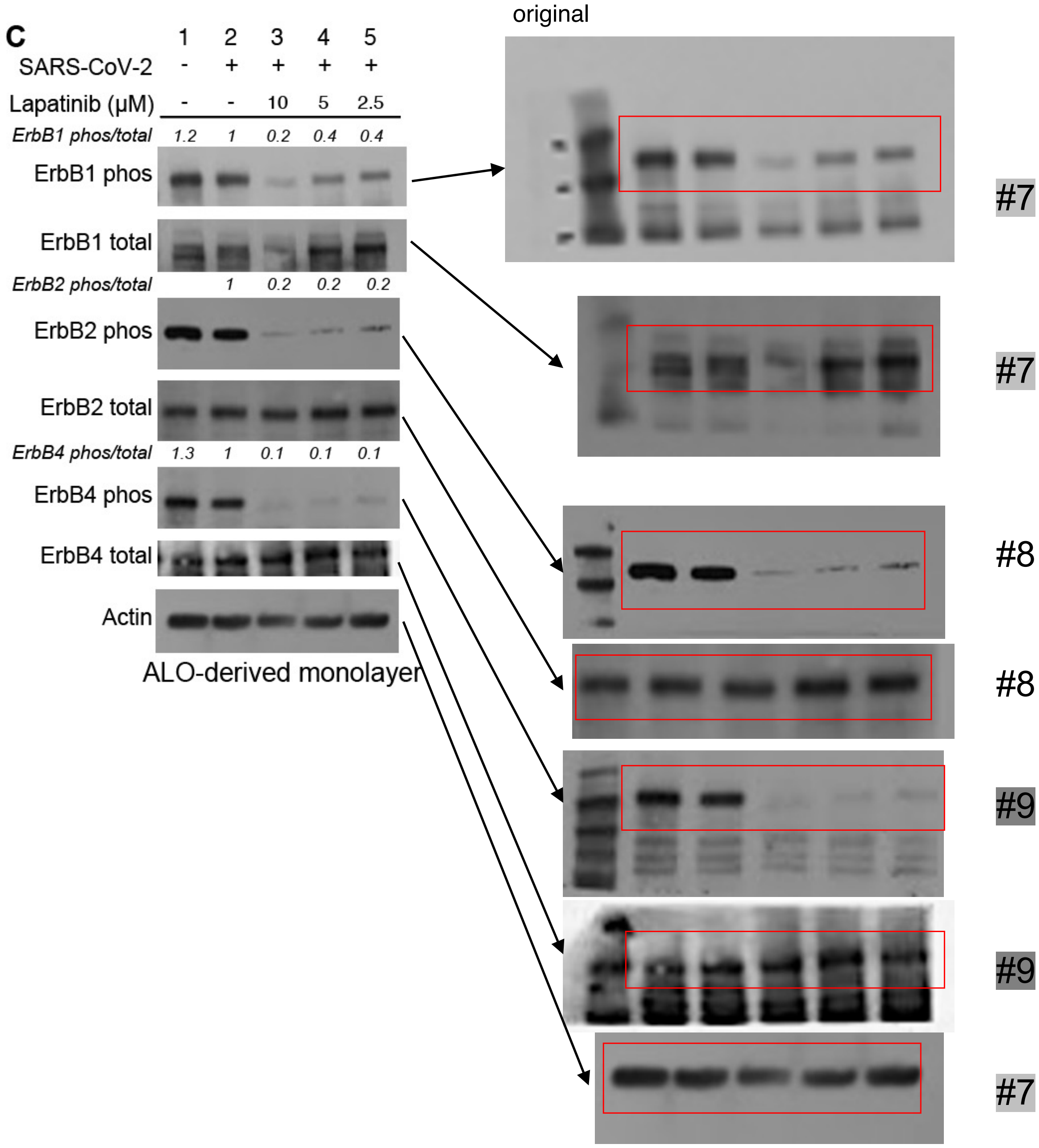

All membranes were cropped prior to blotting to facilitate staining with multiple antibodies. Numbers in Fig. 7C and Fig 7I indicate parts originating from the same membrane. The membranes were blotted first for phosphoproteins, stripped and then blotted for the corresponding total protein.

Supplemental Figure 7I

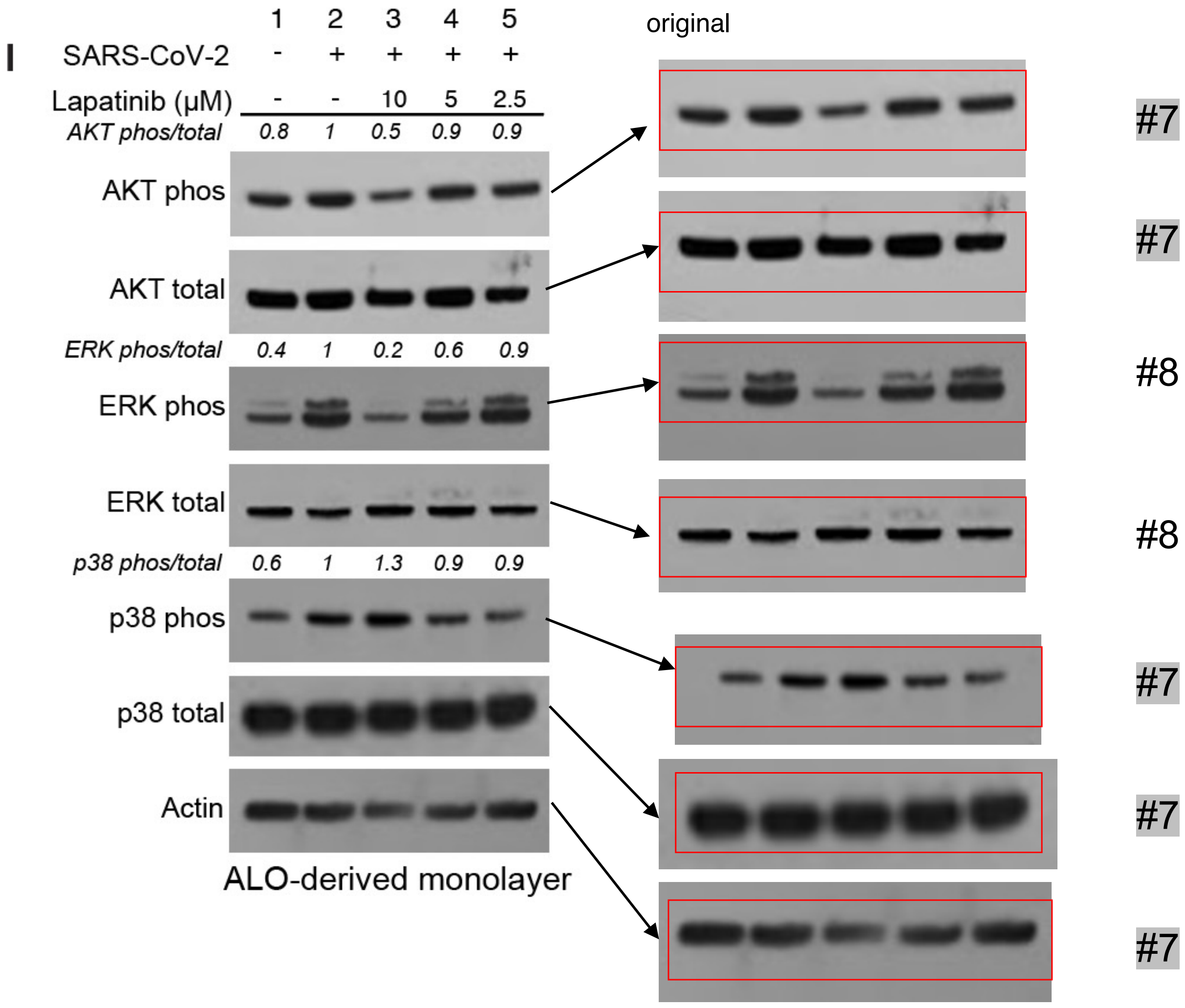

Supplementary Figure 7H

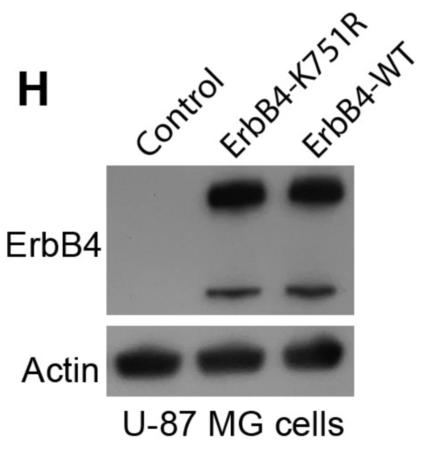

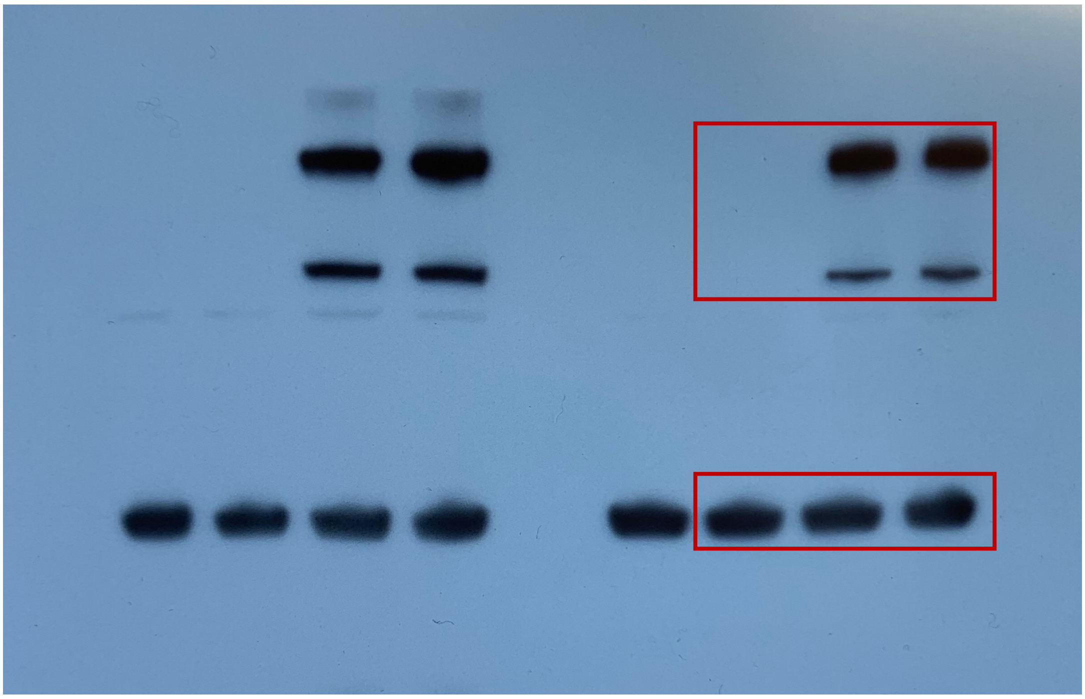

Supplement: Supplemental data [file jci-133-169510-s244.pdf]
